# Identification and characterization of microRNAs and endogenous siRNAs in *Schistosoma japonicum*

**DOI:** 10.1186/1471-2164-11-55

**Published:** 2010-01-21

**Authors:** Lili Hao, Pengfei Cai, Ning Jiang, Heng Wang, Qijun Chen

**Affiliations:** 1 Laboratory of Parasitology, Institute of Pathogen Biology/Institute of Basic Medicine, Chinese Academy of Medical Sciences (CAMS), Beijing 100730, PR China; 2 Key Laboratory of Zoonosis, Ministry of Education, Institute of Zoonosis, Jilin University, Changchun 130062, PR China; 3 Swedish Institute for Infectious Disease Control and Karolinska Institutet, 171 82, Stockholm, Sweden

## Abstract

**Background:**

Small endogenous non-coding RNAs (sncRNAs) such as small interfering RNA (siRNA), microRNA and other small RNA transcripts are derived from distinct loci in the genome and play critical roles in RNA-mediated gene silencing mechanisms in plants and metazoa. They are approximately 22 nucleotides long; regulate mRNA stability through perfect or imperfect match to the targets. The biological activities of sncRNAs have been related to many biological events, from resistance to microbe infections to cellular differentiation. The development of the zoonotic parasite *Schistosoma japonicum *parasite includes multiple steps of morphological alterations and biological differentiations, which provide a unique model for studies on the functions of small RNAs. Characterization of the genome-wide transcription of the sncRNAs will be a major step in understanding of the parasite biology. The objective of this study is to investigate the transcriptional profile and potential function of the small non-coding RNAs in the development of *S. japanicum*.

**Results:**

The endogenous siRNAs were found mainly derived from transposable elements (TE) or transposons and the natural antisense transcripts (NAT). In contrast to other organisms, the TE-derived siRNAs in *S. japonicum *were more predominant than other sncRNAs including microRNAs (miRNAs). Further, there were distinct length and 3'end variations in the sncRNAs, which were associated with the developmental differentiation of the parasite. Among the identified miRNA transcripts, there were 38 unique to *S. japonicum *and 16 that belonged to 13 miRNA families are common to other metazoan lineages. These miRNAs were either ubiquitously expressed, or they exhibited specific expression patterns related to the developmental stages or sex. Genes that encoded miRNAs are mainly located in clusters within the genome of *S. japonicum*. However, genes within one cluster could be differentially transcribed, which suggested that individual genes might be regulated by distinct mechanisms during parasite development.

**Conclusions:**

Many miRNA and endogenous siRNA transcripts were identified in *S. japonicum *and the amount of siRNA was at least 4.4 and 1.6 times more than that of miRNA in both schistosomulum and adult worm stages respectively. SiRNAs are mainly derived from transposable elements (or transposons); while natural antisense transcripts (NAT)-derived siRNAs were much less. A majority of miRNA transcripts identified in the parasite were species-specific and the expression of certain miRNAs was found developmentally regulated. Both miRNA and siRNAs are potentially important regulators in the development of schistosomal parasites.

## Background

Small endogenous non-coding RNA (sncRNA) transcripts including small interfering RNA (siRNA), microRNA (miRNA) and Piwi-interacting RNA (piRNA) are critical regulators in RNA-mediated silencing in plants and metazoa[1,2]. These small RNAs, which are approximately 22 nucleotides long, guide the RNA-induced silencing complex (RISC) to their target sites and exert regulatory functions including chromatin modelling, post-transcriptional repression and mRNA destabilization, which is usually through pairing within the 3'untranslated region of target mRNAs[1].

The biogenesis and regulation of sncRNAs vary among different organisms. SiRNAs are frequently found derived from transposable elements (also called transposons), repeated sequences and antisense strands of protein-coding mRNA templates[2], while miRNAs are encoded by genes either clustered or dispersed in the genome. They can be intergenic or intragenic. MiRNA genes within introns or downstream of the rRNA genes are co-transcribed with the host genes, while the genes independently located are likely controlled by a separated mechanism. Further, the sequences of endogenous siRNA transcripts seem to be more diverse, whereas conservation patterns do exist in certain miRNAs[1,3]. For example, homologues of the temporal miRNAs, lin-4 and let-7 originally identified in *Caenorhabditis elegans*, have been found in a variety of eukaryotes, though they might not exert similar functions in different host cells[2].

While the knowledge regarding sncRNA biology is rapidly expanding, there is little known in schistosomes. These are parasites of at least seven developmental stages that can cause human or zoonotic schistosomiasis, which affect more than 200 million people worldwide[4]. Schistosomes are the lowest group of bilateria that diverged early from the metazoan lineage, and they are among the first animals to develop sexual dimorphism (dioecious lifestyle) and heteromorphic sex chromosomes[5]. They have seven pairs of autosomes and one pair of sex chromosomes, consisting of approximately 270 Mb of genome sequence per schistosomal parasite[6]. The unique developmental features and the availability of the genome sequences for both *S. mansoni *and *S. japonicum *has made it possible to conduct genome-wide transcriptomal and functional characterization of sncRNAs in these parasites.

## Results and Discussion

### Discovery of sncRNAs in *S. japonicum*

We identified and characterized microRNAs and endogenous siRNAs in *S. japonicum *both in the schistosomula (14 days post infection) and in the adult worms by direct sequencing using Solexa sequencing technology, as this technique achieves a more complete coverage of small RNA transcripts than a traditional cDNA cloning approach[7-9]. Sequencing of small RNAs yielded 6,400,876 and 5,323,610 unfiltered sequence reads from adult worms and schistosomula, respectively. After removal of reads containing ambiguous base calls, there were 5,349,115 (mixed-sex adult worms) and 4,273,194 (hepatic schistosomula) clean reads, which contained 1,193,825 and 1,135,641 unique clean reads, respectively. These unique clean reads were mapped to the genome of *S. japonicum *by forcing perfect alignments beginning at the first nucleotide and retaining the longest region of overlap for each read. After elimination of sequences of tRNA, rRNA and mitochondrial DNA-derived RNAs (Additional file 1), potential miRNAs and siRNAs were further analyzed[9,10].

**Figure 1:**
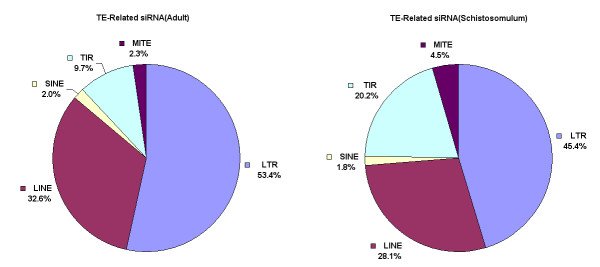
**Percentage of siRNAs derived from different transposable elements (TE) in adult and schistosomulum stages**. Majority of siRNAs were derived from LTR, LINE and TIR.

**Figure 2:**
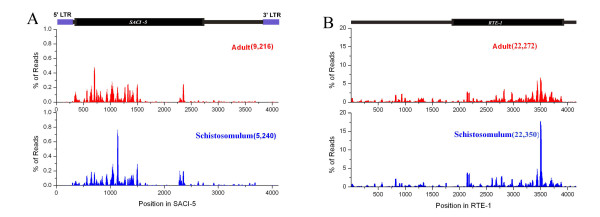
**The abundance of each siRNA sequence generated from the two transposons by scanning each siRNA sequence against the two transposons (A. SACI-5 and B. TRE-1)**. Data represents the percentage of all matched siRNAs identified in adult (red) and schistosomulum (blue) parasites.

Of the small sncRNAs transcripts identified in *S. japonicum*, a large portion was from potential transposable elements (TE) (Additional files 2, 3, 4, 5 and 6). Due to the fact that these sncRNAs showed similar structural signatures to siRNA transcripts reported in *Drosophila*[10], the small RNA transcript sequences mapped to the transposable elements (TE) and the natural antisense transcripts (NAT) were regarded as endogenous siRNAs[11,12]. Since TEs have not been previously identified in *S. japonicum*, we conducted TE prediction by scanning the genome sequence using REPET software. Long terminal repeats (LTR), long interspersed nucleotide elements (LINE), short interspersed elements (SINE), large terminal inverted repeats (TIR) and miniature inverted-repeat transposable elements (MITE) were the main classes of transposons identified in the genome. We found that TE-related sequences accounted for 13 percent of the *S. japonicum *genome (Additional file 7), which is lower than that observed in mammalian DNA that can range between 30 and 50% [13,14]. Further, SiRNAs derived from LTR, TIR and LINE accounted for the majority and transcripts from sense and antisense strands were identified (Fig. 1, Additional files 2, 3, 4, 5 and 6). Sequence scanning of the two transposons SACI-5 (LTR class retrotransposon) [15] and RTE-1 (LINE class) revealed that siRNAs were derived from more concentrated regions in the sequence (Fig. 2a, b), contrast to that found in *Drosophila*, in which siRNAs were generated uniformly from the whole transposon sequence[10].

Furthermore, siRNAs accounted for a major portion of the sncRNAs identified, while miRNAs contributed less. In adult parasites, there were 807,672 TE-derived siRNAs including 120,582 unique clean reads, and there were 495,982 miRNAs including 12,443 unique clean reads (Additional file 7). In schistosomulum, there were 1,495,593 TE-derived siRNAs including 137,439 unique clean reads, and there were 307,984 miRNAs reads including 9,231 unique clean reads (Additional file 7). Thus, more TE-derived siRNAs in schistosomulum were obtained than in the adult, while the number of miRNAs obtained from the two stages was just opposite, i.e., more miRNAs were identified in adult worms than in schistosomulum. On the other hand, miRNAs were more abundant, and siRNAs only accounted for a small portion in *Drosophila *somatic cells[10]. The data indicated that *S. japomicum*, compared to *Drosophila*, may possess a different regulation mechanism in sncRNA transcription.

Until recently,the endogenous siRNAs identified in *Drosophila *and mouse[10,16] were found to be mainly derived from transposable elements, complementary annealed transcripts and long 'fold-back' transcripts (hpRNAs). Natural antisense transcripts (NAT) generated from bidirectional gene transcription are the second source of endogenous siRNAs [17,18]. SiRNAs of approximately 21 nt derived from double-stranded RNA are associate with Ago2 in *Drosophila*[10]. In contrast, piRNAs of 24-32 nt, which are only functional in the germline, appear to be Dicer-independent and are associated with Piwi proteins[2]. We found Ago2 homologues in *S. japonicum *using a BLAST search, but we did not identify a Piwi homologue (data not shown). NAT-derived siRNAs were identified from overlapped regions of mRNAs of 153 predicted genes, and almost all of them were trans-NAT siRNAs. There were 14,427 NAT-derived siRNAs including 2,176 unique clean reads in adult worms and 5,236 NAT-derived siRNAs including 1,241 unique clean reads in schistosomulum (Additional file 7), indicating that the biogenesis of NAT-derived siRNAs in the parasite might be associated with the activation of stage-specific genes. Further analysis found that the overlapped regions of mRNA transcripts where most of the siRNAs were generated from were almost exclusively <450 bp (data not shown) and the number of siRNAs identified from sense and antisense stands were almost equal (data not shown), which indicated that they were not degraded mRNA templates. Collectively, these data revealed that the two main pathways of siRNA biogenesis in *S. japonicum *are processing of TE-derived transcripts and endogenous double strand RNAs (dsRNAs) and the TE-derived siRNAs accounted for the main portion of the siRNA pool, at least in the two development stages of the parasite.

### Length and end variations of sncRNAs in *S. japonicum*

**Figure 3:**
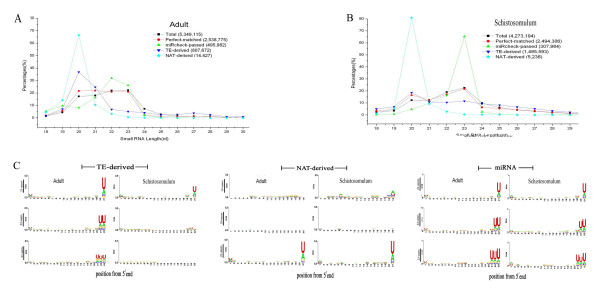
**Small interfering RNAs and microRNAs identified by high-throughput sequencing in *S. japonicum***. **A and B **Length and distribution of siRNAs and miRNAs in adult and schistosomulum stages. **C. **3' end variations in TE and NAT-derived siRNAs and miRNAs.

The variations in the length of sncRNAs in *S. japonicum *were associated with the developmental stages of the parasite. In adult worms, there was an even distribution of the amount of sncRNAs that were between 20 and 23 nt (Fig 3a,b). The TE-derived siRNAs were predominantly 20 nt in adult worms, whereas they exhibited a wider range in length in schistosomulum between 18 and 28 nt (Fig. 3a,b). The NAT-derived siRNAs were predominantly 20 nt in parasites of both schistosomulum and adult stages. Thus TE-derived siRNAs and NAT-derived siRNAs were generated under different regulatory and processing mechanisms. The size of the miRNAs also differed between the two developmental stages. In adult worms, miRNAs ranging from 19 to 23 nt were found, although the miRNAs of 22 and 23 nt accounted for the majority (Fig. 3a). However, miRNAs of 23 nt were predominant in schistosomulum (Fig. 3b) indicating that several miRNA processing pathways might be functional in different developmental stages of the parasite.

In adult worm, all TE-derived siRNAs had the tendency of ending with a uracil residue, while in schistosomulum, only the 21 nt siRNAs were with this composition (Fig. 3c). Of the NAT-derived siRNAs, the 19 nt siRNAs ended with either an adenosine or a uracil in schistosomulum but not in the adult worm, while the 21 nt siRNAs from both stages had a similar sequence at the 3' ends (Fig. 3c). The sequences of both miRNA and siRNA completely match to the genome sequence suggesting that there is no sequence modification in the generation of small RNAs, which is similar to that found in *Drosophila *[10].

Characterization of miRNAs derived from four developmental stages of *S. japonicum *including cercariae, hepatic schistosomulum, adult worm and egg was performed. The miRNAs were between 22 and 23 nt in the adult and predominantly 23 nt in schistosomulum (Fig. 3a,b). Interestingly, miRNAs of all sizes predominantly ended with a uracil, and there were no observed stage-related variations (Fig. 3c3c). This was different from miRNAs derived from other metazoan species, which always begin with a uracil[10]. It remains further investigation whether this is due to the processing of species-specific pre-miRNA or due to functional restriction of the RISC.

**Figure 4:**
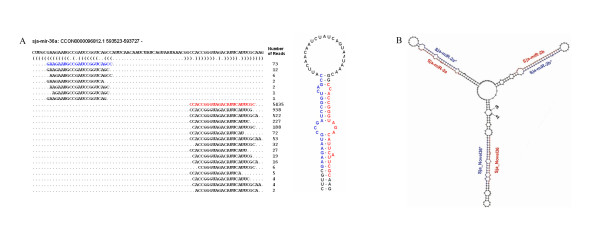
**Repertoire of Sja-mir-36a isomers and a typical miRNA cluster**. **A. **Sequences and the number of reads of the mature miRNA of Sja-mir-36a and the complementary miR* are represented in red and blue respectively. The predicted structure of the pre-miRNA is represented on the right side. **B. **The predicted secondary structure of a pre-miRNA containing a cluster of Sja-mir-2a, Sja-mir-2b, and Sja-novel-36, which form three hairpin stem-loop structures. The sequences of the mature miRNAs are shown in red and the miRNA* strands are shown in blue.

MiRNAs are generated from hairpin structures formed by the complementary sequences within one transcript. After processing by the enzyme Dicer, two complementary sequences (strands) will be released. In this study, there was a diverse variety of miRNA isomers observed. In general, if a dominant sequence was observed, the complementary sequence was also more frequently observed but with significantly different frequency. For example, sja-mir-36a was one of the dominant miRNAs observed (Fig. 4a, Additional files 8,9), and its complementary strand sequence (miR*) was also commonly observed (highlighted in blue), but it was significantly less stable than its counterpart. Furthermore, the number of reads for multiple mature miRNA strands derived from the same precursor differed dramatically, which indicated that the hydrolysis process mediated by Dicer and its associated proteins might not be very precise and the miRNA stability might be determined by the terminal nucleotide residues.

Earlier study has found that expression of miRNA genes can be both monocistronic and polycistronic[19]; however, little is known regarding the genetic regulation of miRNA expression. Certain miRNA-encoding genes are located within the introns of host genes; therefore, they might be transcriptionally regulated through their host-gene promoters[20]. In this study, we found that miRNA genes were predominantly intergenic in *S. japonicum*, thus most miRNA genes have their own control elements ( or promoters) in the genome [21]. Surprisingly, we found that genes within the same cluster exhibited an asymmetrical transcription pattern. For instance, Sja-mir-2a, 2b and Sja-novel-36 were situated within one cluster (Fig. 4b, Table 1 and 2); however, the number of reads of Sja-mir-2a and Sja-novel-36 could be 100 times less than that of Sja-mir-2b. Even though it cannot be ruled out that the differences in reads among the miRNAs encoded in the same cluster were due to experimental factors, it is also possible that several layers of regulation may control miRNA transcription within the same gene cluster.

**Table 1:** Common miRNAs identified in *S. japonicum*

MicroRNA Name	Mature Arm	miR*^a^	Most abundant sequence	Len	Expression^c ^(TPM^b^)		
					AduMix	schistosomulum	P-value^d^
Sja-mir-71	5'	Y	UGAAAGACUUGAGUAGUGAGACG	23	16813	32629	0.
Sja-mir-7	5'	Y	UGGAAGACUGGUGAUAUGUUGUU	23	4256	9969	0
Sja-let-7	5'	Y	GGAGGUAGUUCGUUGUGUGGU	21	2650	2840	0
Sja-mir-2b	3'	Y	UCACAGCCAGUAUUGAUGAACG	22	4004	1104	0
Sja-mir-124	3'	Y	UAAGGCACGCGGUGAAUGUCA	21	1542	386	0
Sja-mir-36a	3'	Y	CCACCGGGUAGACAUUCAUUCGC	23	715	641	0.0263
Sja-mir-10	5'	Y	AACCCUGUAGACCCGAGUUUGG	22	238	440	0
Sja-mir-219	5'	Y	UGAUUGUCCAUUCGCAUUUCUUG	23	494	103	0
Sja-mir-8	3'	N	UAAUACUGUUAGGUAAAGAUGCC	23	159	78	0
Sja-mir-2a	3'	Y	UAUCACAGCCCUGCUUGGGACACA	24	163	10	0
Sja-mir-36b	3'	Y	CCACCGGGUAGACAUUCAU	19	9	11	0.2002
Sja-mir-1810	5'	N	CUAAUAGGGAACGUGAGCU	19	8	6	0.3083
Sja-mir-281	3'	Y	UGUCAUGGAGUUGCUCUCUAU	21	10	1	0
Sja-mir-76	3'	Y	UUCGUUGUUGAUGAAACUGG	20	4	1	0
Sja-mir-307	3'	Y	UCACAACCUACUUGAUUGAGG	21	4	1	0.0001
Sja-mir-923	5'	N	AAGCGGAGGAAAAGAAAU	18	1	1	0.1216

^a^Y indicates that the sequences from both strands of a miRNA* species were found, while N means that only the sequence from one strand of a miRNA* was identified.n

^b^The abundance value of each miRNA was normalized to "transcripts per million (TPM)". If the value after normalization was less than 1, the normalized value was set as 1.

^c^The expression of miRNA was the sum of the total counts of unique reads which was within ±2 nt variations of the mature miRNA on the precursor.

^d^The differentially expressed miRNAs were analyzed using general Chi-square tests.

Len means length of miRNAs. Adumix means miRNA of parasites of mixed adult.

### Identification and characterization of developmental stage-associated miRNAs in *S. japonicum*

**Figure 5:**
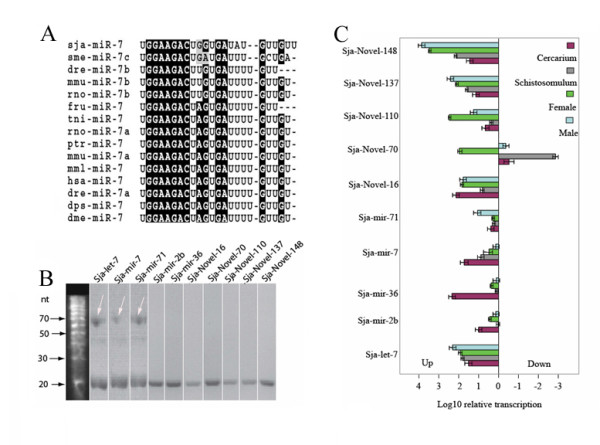
**Sequence and transcriptional analysis of miRNAs dominantly expressed in *S. japonicum***. **A. **Alignment of sja-mir-7 sequences with homologues from other organisms. The seed sequences are shadowed in a dark colour. **B. **Northern-blot hybridization using probes complementary to the dominant miRNAs expressed during the adult worm stage. The 70 nt pre-miRNAs were indicated by arrows. **C. **Quantification analysis, relative to that in the egg stage, of miRNAs dominantly expressed in four developmental stages as indicated in different colours.

For further categorization of the miRNAs, alignments were performed with miRNA sequences derived from *S. japonicum *and miRNAs from other organisms using the ClustalW 1.8 program[22]. Similar to the study on the planarian *Schmidtea mediterranea *[23-25], many miRNAs discovered in the parasite were common to other metazoan lineages as well as miRNAs that were unique to *S. japonicum*. There were 16 miRNAs classified as common miRNAs with evolutionarily conserved characteristics related to 13 metazoan miRNA families including let-7, miR-71, miR-2, and miR-36 (Fig. 5a, Table 1and Additional file 10). Of the commen miRNAs, 13 showed high similirity to that reported in *S. mediterranea *[23-25]. MiRNAs unique to *S. japonicum *(here regarded as novel miRNAs) were also identified (Table 2and Additional files 11,12).

**Table 2:** Top 20 novel abundantly-expressed miRNAs in *S. japonicum*

MicroRNA Name	Mature Arm	miR*^a^	Most abundant sequence	Len	Expression^c ^(TPM^b^)		
					AduMix	schistosomulum	P-value^d^
Sja-Novel-16	5'	Y	UGAUAUGUAUGGGUUACUUGGU	22	16802	1405	0
Sja-Novel-137	5'	Y	AGAGGUAGUGAUUCAUAUGACU	22	8800	3484	0
Sja-Novel-110	3'	Y	UGAGAUCGCGAUUAAAGCU	19	6477	6	0
Sja-Novel-148	5'	Y	UCCCUGAGACUGAUAAUUGCU	21	4888	909	0
Sja-Novel-70	5'	Y	UCAGCUGUGUUCAUGUCUUCGA	22	843	1	0
Sja-Novel-168	3'	Y	UAUUAUGCAACGUUUCACUCU	21	164	439	0
Sja-Novel-166	3'	Y	UGAGAUUCAAUUACUUCAACU	21	345	8	0
Sja-Novel-37	3'	Y	UAUUGCACUUACCUUCGCCUUG	22	169	57	0
Sja-Novel-173	5'	N	CAGACUCACAGAAAUGCUAA	20	31	29	0.159
Sja-Novel-245	5'	Y	UCUUUGGUUAUCAAGCAAUAUGA	23	46	10	0
Sja-Novel-239	3'	N	CUGAGAAUCUGUUGGAUGUU	20	23	27	0.001
Sja-Novel-255	3'	N	GGCGGAUAGGGAGUUGGCGU	20	35	13	0
Sja-Novel-120	3'	N	ACGAGGGCGCUGCAGGGGUUUU	22	24	2	0
Sja-Novel-121	3'	N	GCCAAGACGCGUCACGACAUUU	22	20	4	0
Sja-Novel-35	5'	N	UGGACACAGUAGCCUAGUGGUU	22	16	7	0.0008
Sja-Novel-36	3'	Y	UAUCACAGUCCAAGCUUUGGUAA	23	10	13	0.0054
Sja-Novel-147	5'	N	UGGCAAGAUUACGGCGAAGCU	21	18	4	0
Sja-Novel-128	5'	N	AAGCGUUCGGACGUUGGCAC	20	19	1	0
Sja-Novel-203	5'	N	AGUUAUAUUUAAGUUGGAUUUU	22	8	12	0.0012
Sja-Novel-21	5'	Y	AAGUUCUGAUAGAUGUUGCA	20	16	4	0

^a^Y indicates that the sequences from both strands of a miRNA* species were found, while N means that only the sequence from one strand of a miRNA* was identified.

^b^The abundance value of each miRNA was normalized to "transcripts per million (TPM)". If the value after normalization was less than 1, the normalized value was set as 1.

^c^The expression of miRNA was the sum of the total counts of unique reads which was within ±2 nt variations of the mature miRNA on the precursor.

^d^The differentially expressed miRNAs were analyzed using general Chi-square tests.

Len means length of miRNAs. Adumix means miRNA of parasites of mixed adult.

Real-time PCR was used to relatively quantify the dominantly expressed miRNAs, and the results were confirmed by Northern blotting (Fig. 5b, c). In general, more miRNAs were expressed at stages other than the egg, except Sja-mir-2b and Sja-mir-71, which were constantly expressed during all stages with less than 10-fold variations (Fig. 5c). The expression of Sja-mir-7 and 36 was predominant in the cercarial stage. MiR-7 has been reported to regulate segmentation and sensory organ development in *Drosophila*, and miR-36 has been associated with the transition from the embryo to L1 stage in *C. elegans*[24,25]. Thus it is reasonable to observe significantly increased expression of the homologous miRNAs (Sja-mir-7 and 36) in the cercariae stage of *S. japonicum*, since this is the stage the parasite quickly transforms into schistosomulum after invasion into a mammalian host. The miRNA let-7, which was originally identified to be dominantly expressed during the L3/L4 transition stage in *C. elegans*[24], played a critical role in stage-specific differentiation (timing of cell fate determination). Its homologous miRNA Sja-let-7 was also identified in *S. japonicum*. In contrast to its counterpart in *C. elegans*, Sja-let-7 was expressed in all developmental stages with a similar magnitude except during the egg stage, indicating that this miRNA has evolved to other functions than developmental timing in the schistosomal parasite (Fig. 5c). Mir-71 was previously found to be expressed in the *Drosophila *embryo, and it was implicated in controlling cell differentiation[26]. However, the homolog to mir-71, Sja-mir-71, was predominantly expressed in male parasites. There were also miRNAs identified that were predominantly expressed in female parasites, such as Sja-Novel-70; its expression was approximately 1000 times higher in females, as compared to cercariae, male worms and eggs. The amount of Sja-Novel-70 transcripts expressed during the female stage was one hundred thousand times higher than that in the schistosomulum stage (Fig. 5c). Even though the expression of Sja-Novel-110 was more predominant in female worms, its expression was also detected in males. The discovery of these sex-related miRNAs indicated that miRNAs might regulate parasite sexual differentiation or reproduction machinery. Since schistosomal parasites are among the first animals to develop sexual dimorphism[5], further study on this aspect may result in increased understanding of the molecular control of female and male development. Five miRNAs in *S. japonicum *have been previously identified via a conventional cDNA cloning and sequencing approach[27]. Compared to the previous report, apart from the reported miRNA transcripts, more miRNAs common to other organisms as well as novel miRNA transcripts were identified in this study due to the powerful coverage of the large scale sequencing capacity of Solexa technology. Further, we identified and characterized endogenous siRNAs which were dominantly derived from transposable elements in the genome of the parasite.

### Conclusions

Schistosomiasis is one of the most important human helminth infections[28] that affects more than 200 million people worldwide. Currently, praziquantel is the only drug available for treating schistosomiasis. Investigations focused on parasite biology and identification of novel drug targets have become of great importance. Here we have identified a panel of common as well unique miRNAs and siRNAs in *S. japonicum*. The identification and characterization of siRNAs and miRNAs in the parasite and their possible biological functions have opened a new avenue towards a final dissection of parasite biology and will hopefully facilitate the discovery of potential anti-parasitic drug targets.

## Methods

### Parasites

Adult worms and eggs of *S. japonicum *were isolated from the infected rabbits at 7 weeks post-infection. Male and female adult worms were manually separated under a light microscope. Hepatic schistosomula were isolated from the infected rabbits at 2 weeks post-infection. Cercariae were harvested from the intermediate host, the snail *O. hupensis hupensis*. Parasites of all stages (adult worms, hepatic schistosomula, cercariae and eggs) were stored in RNAlater Solution (Invitrogen, CA, USA) before RNA preparation according the manufacture's protocol.

### RNA isolation

Total RNA of *S. japonicum *(egg, cercariae, schistosomulum and adult worm) was extracted using Trizol reagent (Invitrogen, CA, USA). For complete precipitation of small RNA, after addition of isopropanol, the RNA extract was incubated overnight at -20°**C**.

### Small RNA library construction

Approximately 20 μg of total RNA was size-fractionated on a 15% TBE urea polyacrylamide gel and a 15-30 base-pair fraction was excised and ligated with proprietary adapters to the 5' and 3' termini of the RNA. The RNA was converted to single-stranded cDNA using Superscript II reverse transcriptase (Invitrogen, CA, USA) and Illumina's small RNA RT-Primer following the manufacturer's instructions. The cDNA was PCR-amplified with Hotstart Phusion DNA Polymerase (New England Lab, USA) in 15 cycles using Illumina's small RNA primer set. The PCR products resolved in a 12% TBE urea polyacrylamide gel were eluted with elution buffer, quantified and subjected to Solexa's proprietary sequencing-by-synthesis method.

### Bioinformatic analysis of sequencing data

Individual sequence read with the base quality scores was produced by Solexa. Adapter sequences were removed from both ends of Solexa reads. All identical sequences were counted and eliminated from the initial data set. The resulting set of the unique sequences with associated read counts was referred as sequence tags. The unique reads were mapped onto the *S. japonicum *genome of SGSThttp://lifecenter.sgst.cn using the program SOAP[29].

To identify potential miRNA sequences, the perfectly matched reads were Blast-searched against the metazoan mature miRNA of Sanger miRBase [30] (Release 12) using the program Patscan[31]. Conserved and novel miRNAs were predicted as described[7]. Using similar approach to credibility interval approaches reported for the analysis of SAGE data [32], we employed IDEG6[33] to identify miRNAs showing statistically significant difference in relative abundance (as reflected by total count of individual sequence reads) between the two small RNA libraries from schistolomulum and adult worm stages. We used the general Chi2 method, because it resulted the most efficient tests[33]. Finally, miRNA with a P value <= 0.01 were deemed to be significantly different between the two samples.

Secondary structure predictions of the miRNAs cluster were performed using MFOLD Version 2.38 using the default folding conditions (1 M NaCl, 37°C). To generate graphical output, the mfold predictions were imported into RnaViz9 (Version 2.0). Multiple sequence alignments were produced by ClustalW 1.84 and graphical display of the output was performed using Boxshade 3.21.5.

The transposable elements (TEs) in the *S. japonicum *genome were predicted by using REPET http://urgi.versailles.inra.fr/development/repet/. The reads that perfectly matched *S. japonicum *genome were aligned to unique consensus sequences of TEs using the program SOAP[29]. The reads that perfectly matched TEs were considered as TE-derived siRNAs.

The natural antisense transcripts (NATs) were detected by aligning the predicted sequences by SGST http://lifecenter.sgst.cn to each other. If a pair of overlapping sequences was matched at opposite strand and the length of overlap ≥ 100 bp and the identity of the overlaps was ≥ 95%, this pair of overlapping sequences was defined as NATs pair. The reads that perfectly matched *S. japonicum *genome were aligned to overlapped sequences of NAT pairs using tSOAP[29]. The reads that perfectly matched the overlap region were considered as NAT-derived siRNAs.

### Northern blot analysis of miRNA transcripts

Northern blot was performed according to the method described[34]. Thirty micrograms of total RNA were resolved on a 15% denaturing polyacrylamide gel. The RNA was transferred onto a sheet of nylon membrane (Hybond-NX, Amersham/Pharmacia, Sweden) and EDC-crosslinked. Oligonucleotide probes complementary to the miRNAs were ^32^P-end-labeled with T4 kinase and hybridized in 2 × SSC, 1% SDS buffer containing 100 μg/ml sheared, denatured salmon sperm DNA at temperatures between 40 and 60°C. Post-hybridization washes were carried out in 0.1 × SSC, 0.2% SDS buffer at temperatures ranging from 40 and 60°C. All isotopic images were recorded using a FLA-5000 system (Fuji, Japan) with Aida Image Analyser software. RNA markers were ^32^P-end-labeled Decade RNA markers (Ambion, USA) prepared according to the manufacturer's instructions.

### MiRNA quantification by real-time PCR

Total RNA (1 μg) was polyadenylated with Poly(A) Tailing Kit (Ambion, California, USA) at 37°**C **for 1 h in a 20 μl reaction mixture following the manufacturer's instruction. After phenol-chloroform extraction and ethanol precipitation, the RNAs were dissolved in diethylpyrocarbonate (DEPC)-treated water and reverse-transcribed with 200 U SuperScript™ III Reverse Transcriptase (Invitrogen) and the FirstChoice® RLM-RACE kit (Ambion, USA) according to the manufacturer's instruction. The following primers were designed as forward primers of miRNAs:

Sja-let-7 3'-GGAGGTAGTTCGTTGTGTGGT-5'; Sja-mir-2b 5'-TCACAGCCAGTATTGATGAACG-3'; Sja-mir-36 5-'CCACCGGGTAGACATTCATTCGC-3'; Sja-mir-7 5'-TGGAAGACTGGTGATATGTTGTT-3'; Sja-mir-71 5'-TGAAAGACTTGAGTAGTGAGACG-3'; Sja-novel-16 5'-TGATATGTATGGGTTACTTGGT-3'; Sja-novel-70 5'-TCAGCTGTGTTCATGTCTTCGA-3'; Sja-novel-110 5'-TGAGATCGCGATTAAAGCT-3'; Sja-novel-137 5'-AGAGGTAGTGATTCATATGACT-3' Sja-novel-148 5'-TCCCTGAGACTGATAATTGCT-3';

The sequence of 5'-GCGAGCACAGAATTAATACGAC-3' complementary to the adaptor was used as the common reverse primer.

The primers, Pf α-tubulin 5'-CATGGTAGACAACGAAGCTATTTATGA-3' and Pr α-tubulin 5'-GATTAGTGTAGGTTGGACGCTCTATG-3', were used to amplify the α-tubulin transcript as the endogenous control. PCR reactions were set up by combining 0.4 nM final concentration of each primer pair, cDNA, 12.5 μl of Power SYBR Green PCR Master Mix (ABI, USA) adjusted to a final volume of 25 μl with DEPC-treated water in triplicates. Quantification was performed using the ABI PRISM 7300 sequence detection system (Applied Biosystems, Foster City, USA). The Relative expression was analyzed using the SDS 1.4 software (Applied Biosystems, Foster City, USA). PCR efficiency for the amplicon was calculated using the method described by Ruijter et al.[35].

## Authors' contributions

LH and QC conceived and designed the experiments. LH, PC, NJ performed the experiments. LH, QC analyzed the data. HW contributed reagents/materials/analysis tools. LH, PC and QC wrote the manuscript. All authors read and approved the final manuscript.

## Supplementary Material

Additional file 1 **General information of the small RNA library**. This file contains the reads of all small RNA transcripts identified and their relative portion in the library. Click here for file

Additional file 2 **SiRNAs derived from LINE**. This file contains the information of the identified transposon-LINE in the *S. japonicum *genome and the derived siRNAs. Click here for file

Additional file 3 **SiRNAs derived from LTR**. This file contains the information of the identified transposon-LTR in the *S. japonicum *genome and the derived siRNAs. Click here for file

Additional file 4 **SiRNAs derived from SINE**. This file contains the information of the identified transposon-SINE in the *S. japonicum *genome and the derived siRNAs. Click here for file

Additional file 5 **SiRNAs derived from TIR**. This file contains the information of the identified transposon-TIR in the *S. japonicum *genome and the derived siRNAs. Click here for file

Additional file 6 **SiRNAs derived from MITE**. This file contains the information of the identified transposon-MITE in the *S. japonicum *genome and the derived siRNAs. Click here for file

Additional file 7 **Data Summary of siRNAs and miRNAs**. This file contains the summary data of siRNA and miRNA, from stage-associated differences in length distribution to the number of transcripts. Click here for file

Additional file 8 **Hairpin prediction of common miRNAs in *S. japonicum***. This file contains the predicted hairpin structures of common miRNAs. Click here for file

Additional file 9 **Hairpin prediction of novel miRNAs in *S. japonicum***. This file contains the predicted hairpin structures of novel miRNAs. Click here for file

Additional file 10 **Common miRNAs identified in *S. japonicum***. This file contains the information of common miRNAs, from contig identity to stage-associated variation in transcription. Click here for file

Additional file 11 **Hairpin prediction of novel miRNAs in *S. japonicum***. This file contains the predicted hairpin structures of the novel miRNAs, from contig identity to stage-associated variation in transcription. Click here for file

Additional file 12 **MicroRNAs in *S. japonicum *with high homology to that of *S. mediterranea***. This file contains information of the comparison between miRNAs identified in *S. japonicum *and that found in *S. mediterranea*. Click here for file
