# Supplementary material for: Identification and characterization of microRNAs and endogenous siRNAs in Schistosoma japonicum
Source: BMC Genomics. 2010 Jan 21;11:55. doi: 10.1186/1471-2164-11-55 (PMC2820009; doi:10.1186/1471-2164-11-55)
Supplement: Additional file 1 — General information of the small RNA library. This file contains the reads of all small RNA transcripts identified and their relative portion in the library. [file 1471-2164-11-55-S1.PDF]

General information in the libraries

Tab1. Small RNA Classification

Class	Total			Adult			schistosomula		
	# of Unique	Total sequences	%	# of Unique	Total sequences	%	# of Unique	Total sequences	%
Total Perfect Matched	415226	5033081		230095	2538775		270549	2494306	
microRNAs[a]	18030	803966	15.97	12443	495982	19.54	9231	307984	12.35
Non-Coding RNA[b]	10428	232124	4.61	4464	42209	1.66	9352	189915	7.61
rRNA	6933	169745	3.37	2780	21807	0.86	6646	147938	5.93
tRNA	1900	38819	0.77	1213	19108	0.75	1251	19711	0.79
snoRNA	3	4	0.00	1	1	0.00	2	3	0.00
other	1592	23556	0.47	470	1293	0.05	1453	22263	0.89
NAT-Derived siRNAs	2578	19450	0.39	2148	14239	0.56	1234	5211	0.21
TE-Derived siRNAs	212820	2163855	42.99	128159	817004	32.18	139640	1346851	54.00
Transcripts Related	38007	65062	1.29	6526	21822	0.86	33286	43240	1.73
Unknown	133363	1748624	34.74	76355	1147519	45.20	77806	601105	24.10

[a] Passed miRcheck

[b] Sanger Rfam database release 9.0 ,except microRNA

Fig.1

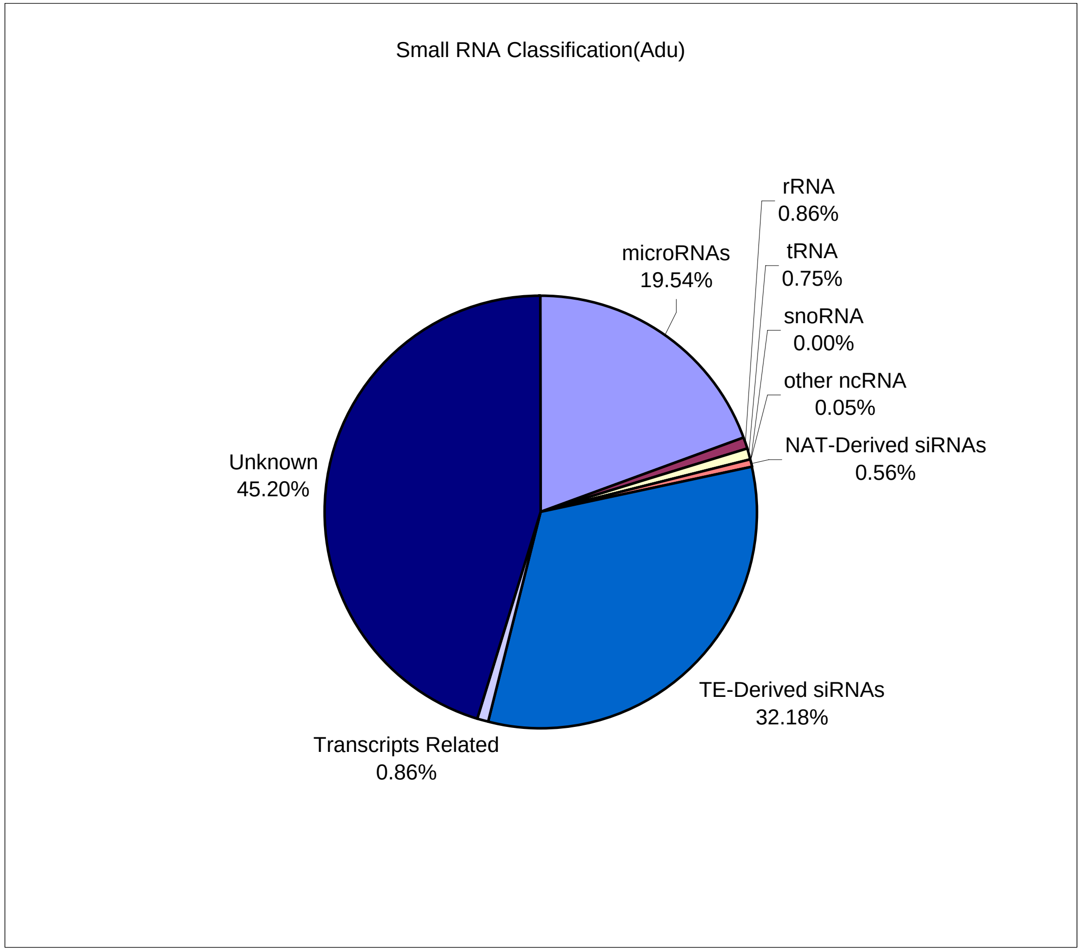

Small RNA Classification(Adu)
rRNA 0.86%
tRNA 0.75%
snoRNA 0.00%
other ncRNA 0.05%
NAT-Derived siRNAs 0.56%
microRNAs 19.54%
Unknown 45.20%
TE-Derived siRNAs 32.18%
Transcripts Related 0.86%

Fig.2

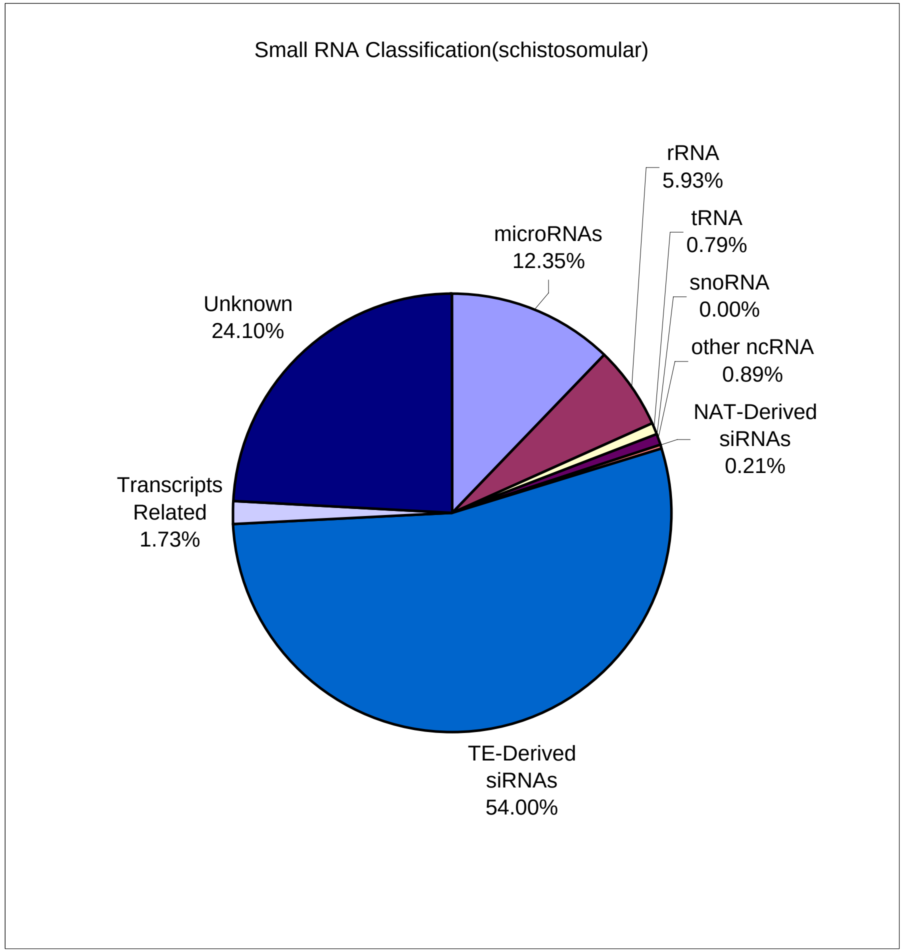

Small RNA Classification(schistosomular)
rRNA 5.93%
tRNA 0.79%
snoRNA 0.00%
other ncRNA 0.89%
NAT-Derived siRNAs 0.21%
microRNAs 12.35%
Unknown 24.10%
Transcripts Related 1.73%
TE-Derived siRNAs 54.00%
