# Supplementary material for: Identification and characterization of microRNAs and endogenous siRNAs in Schistosoma japonicum
Source: BMC Genomics. 2010 Jan 21;11:55. doi: 10.1186/1471-2164-11-55 (PMC2820009; doi:10.1186/1471-2164-11-55)
Supplement: Additional file 2 — SiRNAs derived from LINE. This file contains the information of the identified transposon-LINE in the S. japonicum genome and the derived siRNAs. [file 1471-2164-11-55-S2.PDF]

siRNAs derived from LINE

Name	Type	Annotation	TE Length	siRNAs (Adult)		siRNAs (schistosomula)	
				# Sense siRNAs	AntiSense siRNAs	# Sense siRNAs	# AntiSense siRNAs
Sj_Blaster_Grouper_30934_MAP_3	LINE	Novel	1974	1	0	0	0
Sj_Blaster_Recon_10253_MAP_9	LINE	PERERE-4_2p	3621	0	0	0	1
Sj_Blaster_Grouper_34316_MAP_4	LINE	PERERE-4_2p	1504	1	0	0	0
Sj_Blaster_Recon_15314_MAP_3	LINE	Novel	756	0	0	0	1
Sj_Blaster_Recon_13481_MAP_3	LINE	Novel	859	0	0	0	1
Sj_Blaster_Recon_4685_MAP_3	LINE	Novel	1307	0	0	0	1
Sj_Blaster_Recon_9234_MAP_8	LINE	BN000793_1p	2154	0	1	0	0
Sj_Blaster_Recon_15691_MAP_3	LINE	Novel	524	0	1	0	0
Sj_Blaster_Recon_7342_MAP_3	LINE	Novel	2336	0	1	0	0
Sj_Blaster_Recon_18434_MAP_4	LINE	Novel	391	0	0	0	1
Sj_Blaster_Recon_5966_MAP_7	LINE	Novel	1461	0	2	0	0
Sj_Blaster_Grouper_6395_MAP_3	LINE	Novel	541	1	0	0	0
Sj_Blaster_Recon_11438_MAP_3	LINE	Novel	892	0	0	1	0
Sj_Blaster_Recon_10126_MAP_3	LINE	PERERE-4_2p	1152	0	1	0	0
Sj_Blaster_Recon_4022_MAP_7	LINE	Novel	3644	0	0	1	0
Sj_Blaster_Recon_12516_MAP_3	LINE	Novel	490	1	0	0	0
Sj_Blaster_Recon_320_MAP_3	LINE	Novel	1232	0	1	0	0
Sj_Blaster_Recon_16901_MAP_3	LINE	Novel	1930	0	1	0	0
Sj_Blaster_Grouper_16132_MAP_3	LINE	Novel	1043	0	0	1	0
Sj_Blaster_Recon_6459_MAP_3	LINE	Novel	1303	0	1	0	0
Sj_Blaster_Grouper_13332_MAP_3	LINE	Novel	827	1	0	0	0
Sj_Blaster_Recon_8311_MAP_3	LINE	Novel	1759	0	0	1	0
Sj_Blaster_Grouper_1794_MAP_3	LINE	Novel	404	0	0	0	1
Sj_Blaster_Grouper_15964_MAP_4	LINE	Perere_Smed_1p	1027	0	0	1	0
Sj_Blaster_Grouper_25282_MAP_3	LINE	Novel	2574	0	0	2	0
Sj_Blaster_Recon_5097_MAP_6	LINE	Novel	1034	0	1	0	0
Sj_Blaster_Grouper_18575_MAP_3	LINE	Novel	1316	1	0	0	0
Sj_Blaster_Grouper_32318_MAP_3	LINE	RT	1968	0	0	0	1
Sj_Blaster_Recon_8562_MAP_3	LINE	Novel	597	1	0	0	0

Sj_Blaster_Recon_9902_MAP_5	LINE	Novel	2237	0	0	0	1
Sj_Blaster_Recon_8140_MAP_3	LINE	Novel	648	0	0	2	0
Sj_Blaster_Grouper_30942_MAP_3	LINE	Novel	1554	2	0	0	0
Sj_Blaster_Recon_11145_MAP_6	LINE	BN000793_2p	2924	0	0	1	1
Sj_Blaster_Grouper_4766_MAP_3	LINE	Novel	493	2	0	0	0
Sj_Blaster_Recon_6545_MAP_4	LINE	RT	2759	0	0	1	1
Sj_Blaster_Recon_9159_MAP_4	LINE	Novel	1383	1	1	0	0
Sj_Blaster_Recon_5767_MAP_11	LINE	Novel	6474	0	0	0	2
Sj_Blaster_Grouper_16282_MAP_3	LINE	BN000793_2p	1054	1	1	0	0
Sj_Blaster_Recon_8226_MAP_3	LINE	Novel	1474	1	1	0	0
Sj_Blaster_Recon_8016_MAP_4	LINE	PERERE-4_1p	1765	0	0	1	1
Sj_Blaster_Grouper_25764_MAP_4	LINE	Novel	2701	0	1	1	0
Sj_Blaster_Recon_8106_MAP_3	LINE	Novel	1193	1	0	1	0
Sj_Blaster_Recon_8710_MAP_3	LINE	Novel	2022	0	0	2	0
Sj_Blaster_Recon_6479_MAP_3	LINE	Novel	1547	0	1	1	0
Sj_Blaster_Grouper_27931_MAP_3	LINE	Novel	2507	1	0	0	1
Sj_Blaster_Grouper_20138_MAP_3	LINE	Novel	1547	1	0	2	0
Sj_Blaster_Grouper_6075_MAP_3	LINE	Novel	531	3	0	0	0
Sj_Blaster_Grouper_22354_MAP_3	LINE	Novel	1708	1	0	2	0
Sj_Blaster_Recon_960_MAP_3	LINE	Novel	952	1	0	2	0
Sj_Blaster_Recon_10239_MAP_3	LINE	Novel	650	2	0	0	1
Sj_Blaster_Recon_18106_MAP_3	LINE	SjR2	483	1	1	1	0
Sj_Blaster_Recon_5829_MAP_3	LINE	Novel	532	1	0	4	0
Sj_Blaster_Recon_18477_MAP_4	LINE	PERERE-4_2p	777	0	1	0	0
Sj_Blaster_Grouper_22681_MAP_3	LINE	Novel	1485	0	0	3	0
Sj_Blaster_Grouper_22548_MAP_3	LINE	PERERE-4_1p	1957	0	0	0	3
Sj_Blaster_Recon_13380_MAP_3	LINE	RT	2899	0	3	0	1
Sj_Blaster_Recon_10428_MAP_4	LINE	Novel	2453	1	1	1	0
Sj_Blaster_Recon_1345_MAP_10	LINE	Novel	4310	0	0	4	0
Sj_Blaster_Recon_11391_MAP_4	LINE	Novel	2186	0	1	6	0
Sj_Blaster_Grouper_20908_MAP_3	LINE	SjR2	1662	1	0	4	0
Sj_Blaster_Grouper_9084_MAP_3	LINE	Novel	628	2	0	0	1
Sj_Blaster_Recon_10847_MAP_3	LINE	Novel	1513	0	1	1	2
Sj_Blaster_Recon_9220_MAP_3	LINE	Poseidon_Ap_1p	644	0	0	2	1

Sj_Blaster_Recon_2113_MAP_3	LINE	Novel	606	2	0	1	0
Sj_Blaster_Grouper_23072_MAP_3	LINE	PERERE-4_2p	2036	1	4	0	0
Sj_Blaster_Recon_991_MAP_9	LINE	PERERE-4_1p	1885	0	1	3	0
Sj_Blaster_Recon_3198_MAP_6	LINE	Poseidon_Ap_1p	4249	1	1	0	2
Sj_Blaster_Recon_6277_MAP_3	LINE	Novel	985	0	0	0	1
Sj_Blaster_Recon_956_MAP_5	LINE	RTE-1_MD_1p	3555	1	1	1	1
Sj_Blaster_Grouper_23113_MAP_4	LINE	PERERE-4_1p	2086	1	0	0	3
Sj_Blaster_Recon_762_MAP_4	LINE	Novel	2087	0	1	1	3
Sj_Blaster_Recon_5348_MAP_8	LINE	Novel	2070	2	0	1	1
Sj_Blaster_Recon_2196_MAP_4	LINE	Novel	1220	2	2	1	0
Sj_Blaster_Recon_11312_MAP_4	LINE	PERERE-4_2p	3077	4	0	3	0
Sj_Blaster_Grouper_20684_MAP_3	LINE	Novel	1639	0	0	0	5
Sj_Blaster_Grouper_16543_MAP_9	LINE	Perere_Smed_1p	1133	0	1	1	3
Sj_Blaster_Recon_5150_MAP_4	LINE	SjR2	1385	2	1	1	1
Sj_Blaster_Recon_16463_MAP_5	LINE	Perere_Smed_1p	1711	0	1	1	2
Sj_Blaster_Recon_13673_MAP_3	LINE	Novel	1053	4	1	1	0
Sj_Blaster_Recon_3100_MAP_10	LINE	Novel	3805	0	2	2	0
Sj_Blaster_Grouper_20913_MAP_4	LINE	PERERE-4_1p	1677	0	1	3	1
Sj_Blaster_Recon_5509_MAP_4	LINE	RTE-1_MD_1p	1013	0	1	0	5
Sj_Blaster_Recon_15853_MAP_7	LINE	Novel	4952	3	3	0	0
Sj_Blaster_Grouper_21654_MAP_6	LINE	PERERE-4_1p	1893	0	1	3	1
Sj_Blaster_Grouper_25498_MAP_3	LINE	PERERE-4_1p	2393	1	0	1	3
Sj_Blaster_Grouper_18600_MAP_4	LINE	PERERE-4_1p	1322	2	0	0	3
Sj_Blaster_Recon_6878_MAP_5	LINE	Novel	1133	1	1	4	0
Sj_Blaster_Grouper_31045_MAP_5	LINE	RTE-1_AGp	1475	2	1	1	1
Sj_Blaster_Recon_6407_MAP_3	LINE	Novel	1029	5	0	1	0
Sj_Blaster_Recon_422_MAP_4	LINE	Novel	703	0	1	2	3
Sj_Blaster_Recon_11629_MAP_3	LINE	Novel	1192	0	3	0	2
Sj_Blaster_Recon_535_MAP_4	LINE	PERERE-4_2p	1311	1	1	0	11
Sj_Blaster_Recon_11313_MAP_3	LINE	PERERE-4_2p	1812	1	2	0	1
Sj_Blaster_Grouper_9173_MAP_3	LINE	Novel	631	0	3	1	2
Sj_Blaster_Recon_5740_MAP_3	LINE	SjR2	2841	2	2	1	0
Sj_Blaster_Grouper_19777_MAP_3	LINE	Novel	1493	0	1	2	3
Sj_Blaster_Recon_7175_MAP_11	LINE	Novel	3290	4	2	0	1

Sj_Blaster_Grouper_22240_MAP_4	LINE	Novel	1909	0	0	5	2
Sj_Blaster_Recon_13506_MAP_3	LINE	Novel	723	3	2	1	1
Sj_Blaster_Grouper_25575_MAP_3	LINE	PERERE-4_2p	2054	5	0	2	0
Sj_Blaster_Recon_4043_MAP_6	LINE	PERERE-4_2p	2153	0	5	1	1
Sj_Blaster_Recon_16992_MAP_3	LINE	Novel	1878	1	5	2	0
Sj_Blaster_Grouper_30882_MAP_16	LINE	RTE-1_AGp	1699	3	1	1	1
Sj_Blaster_Grouper_31800_MAP_3	LINE	RTE-1_AGp	2212	1	2	1	2
Sj_Blaster_Recon_8608_MAP_12	LINE	Novel	5065	2	2	0	3
Sj_Blaster_Recon_12099_MAP_3	LINE	RTE-1_MD_1p	1083	0	6	2	1
Sj_Blaster_Recon_8584_MAP_3	LINE	RTE-1_AGp	1253	2	2	1	1
Sj_Blaster_Grouper_30471_MAP_5	LINE	CR1-2_AG-ORF2p	1734	1	3	1	1
Sj_Blaster_Recon_296_MAP_3	LINE	PERERE-4_2p	1735	6	0	1	0
Sj_Blaster_Grouper_17926_MAP_12	LINE	SjR2	1332	3	2	0	0
Sj_Blaster_Recon_11914_MAP_5	LINE	Novel	847	1	2	0	6
Sj_Blaster_Recon_8354_MAP_4	LINE	Poseidon_Ap_1p	3209	4	3	0	0
Sj_Blaster_Recon_10499_MAP_4	LINE	PERERE-4_1p	1043	1	3	5	0
Sj_Blaster_Recon_14266_MAP_3	LINE	SjR2	917	2	1	4	3
Sj_Blaster_Recon_13567_MAP_4	LINE	RTE-1_MD_1p	805	19	0	0	0
Sj_Blaster_Grouper_31150_MAP_4	LINE	RTE-1_AGp	1980	1	2	2	2
Sj_Blaster_Grouper_23955_MAP_5	LINE	Novel	2034	3	0	3	2
Sj_Blaster_Recon_15221_MAP_3	LINE	RTE-1_AGp	1635	4	4	0	1
Sj_Blaster_Grouper_31437_MAP_3	LINE	Novel	2517	3	1	2	1
Sj_Blaster_Grouper_30891_MAP_3	LINE	Novel	1427	0	3	12	1
Sj_Blaster_Recon_18402_MAP_3	LINE	SjR2	133	0	2	3	1
Sj_Blaster_Grouper_19496_MAP_3	LINE	RTE-1_AGp	1445	1	8	1	1
Sj_Blaster_Recon_2077_MAP_20	LINE	Novel	5848	2	0	6	0
Sj_Blaster_Recon_2155_MAP_4	LINE	Novel	1369	2	2	1	1
Sj_Blaster_Grouper_4495_MAP_4	LINE	Novel	486	3	1	6	0
Sj_Blaster_Recon_3346_MAP_3	LINE	SjR2	1321	1	5	2	0
Sj_Blaster_Recon_987_MAP_3	LINE	Novel	669	1	5	2	2
Sj_Blaster_Recon_11362_MAP_3	LINE	Novel	1043	4	2	3	1
Sj_Blaster_Grouper_14062_MAP_7	LINE	PERERE-4_2p	870	2	2	1	5
Sj_Blaster_Recon_605_MAP_3	LINE	Novel	1382	4	5	2	1
Sj_Blaster_Grouper_25895_MAP_3	LINE	RTE-1_AGp	2233	1	1	4	2

Sj_Blaster_Recon_3510_MAP_4	LINE	RTE-1_AGp	3184	3	3	0	5
Sj_Blaster_Recon_2997_MAP_3	LINE	Novel	1495	3	1	3	2
Sj_Blaster_Grouper_31158_MAP_3	LINE	RTE-1_AGp	2174	1	3	2	2
Sj_Blaster_Recon_5166_MAP_5	LINE	Novel	2645	0	0	10	0
Sj_Blaster_Recon_15334_MAP_5	LINE	PENELOPE_1p	4400	2	1	6	2
Sj_Blaster_Recon_6817_MAP_5	LINE	Novel	963	5	3	2	0
Sj_Blaster_Recon_9859_MAP_3	LINE	Novel	1232	1	9	1	0
Sj_Blaster_Recon_2898_MAP_4	LINE	Novel	1451	1	3	2	4
Sj_Blaster_Grouper_30731_MAP_6	LINE	SjR2	1416	3	4	0	1
Sj_Blaster_Recon_28_MAP_4	LINE	SjR2	606	5	5	1	1
Sj_Blaster_Recon_13218_MAP_3	LINE	Novel	730	3	2	3	2
Sj_Blaster_Recon_3333_MAP_9	LINE	Novel	5517	0	6	1	1
Sj_Blaster_Piler_281.315_MAP_3	LINE	Novel	640	0	1	4	4
Sj_Blaster_Grouper_14482_MAP_3	LINE	Novel	906	2	4	3	2
Sj_Blaster_Recon_11314_MAP_4	LINE	PERERE-4_2p	1450	4	0	10	0
Sj_Blaster_Grouper_27591_MAP_3	LINE	PERERE-4_2p	2962	3	5	1	1
Sj_Blaster_Recon_13078_MAP_3	LINE	RTE-1_MD_1p	734	3	1	4	3
Sj_Blaster_Recon_5828_MAP_3	LINE	Novel	929	8	3	5	1
Sj_Blaster_Grouper_30359_MAP_3	LINE	RTE-1_AGp	2653	5	1	3	2
Sj_Blaster_Recon_4402_MAP_3	LINE	Novel	879	9	4	4	2
Sj_Blaster_Grouper_23750_MAP_3	LINE	Novel	2132	0	0	2	10
Sj_Blaster_Recon_3491_MAP_6	LINE	Novel	3992	3	4	5	2
Sj_Blaster_Recon_16223_MAP_3	LINE	Novel	679	0	2	0	8
Sj_Blaster_Recon_3111_MAP_3	LINE	SjR2	2538	1	5	5	1
Sj_Blaster_Recon_3725_MAP_6	LINE	L1PREC2_2p	1515	5	8	0	0
Sj_Blaster_Recon_12673_MAP_4	LINE	PERERE-4_2p	952	0	1	0	12
Sj_Blaster_Recon_13073_MAP_3	LINE	Novel	1215	1	6	3	2
Sj_Blaster_Recon_14762_MAP_3	LINE	SjR2	843	5	0	4	3
Sj_Blaster_Grouper_16023_MAP_3	LINE	Novel	1032	5	3	4	6
Sj_Blaster_Grouper_30941_MAP_3	LINE	Novel	1237	8	1	1	4
Sj_Blaster_Grouper_9446_MAP_3	LINE	Novel	636	1	16	1	1
Sj_Blaster_Recon_8769_MAP_6	LINE	Novel	1783	6	4	1	3
Sj_Blaster_Grouper_30990_MAP_3	LINE	RTE-1_AGp	2331	1	4	2	6
Sj_Blaster_Recon_10818_MAP_3	LINE	RTE-1_AGp	1450	1	8	5	2

Sj_Blaster_Grouper_27684_MAP_3	LINE	RTE-1_MD_1p	1404	4	3	9	1
Sj_Blaster_Recon_12610_MAP_3	LINE	Novel	1731	17	1	1	1
Sj_Blaster_Recon_11302_MAP_3	LINE	SjR2	1084	2	3	4	2
Sj_Blaster_Grouper_29450_MAP_3	LINE	RTE-1_AGp	1257	1	5	1	9
Sj_Blaster_Recon_12294_MAP_3	LINE	RTE-1_MD_1p	572	6	2	4	2
Sj_Blaster_Grouper_25464_MAP_6	LINE	Novel	2613	5	1	4	4
Sj_Blaster_Recon_8700_MAP_6	LINE	Novel	2583	6	0	8	1
Sj_Blaster_Grouper_13998_MAP_8	LINE	PERERE-4_2p	872	2	2	8	9
Sj_Blaster_Grouper_26669_MAP_6	LINE	RTE-1_MD_1p	1562	6	6	0	8
Sj_Blaster_Recon_3839_MAP_4	LINE	Novel	1212	9	5	2	3
Sj_Blaster_Recon_12437_MAP_4	LINE	Novel	1470	6	3	4	6
Sj_Blaster_Recon_10577_MAP_4	LINE	Novel	1097	4	0	1	12
Sj_Blaster_Recon_14473_MAP_3	LINE	Novel	1072	6	1	9	2
Sj_Blaster_Recon_2603_MAP_9	LINE	Novel	2277	0	7	2	3
Sj_Blaster_Recon_12981_MAP_4	LINE	Novel	2797	4	0	4	9
Sj_Blaster_Grouper_25598_MAP_3	LINE	Novel	2636	6	1	3	2
Sj_Blaster_Recon_1400_MAP_20	LINE	Novel	7430	6	1	2	2
Sj_Blaster_Recon_9641_MAP_3	LINE	Novel	1358	3	3	6	8
Sj_Blaster_Grouper_3070_MAP_3	LINE	SjR2	445	1	10	1	3
Sj_Blaster_Recon_3201_MAP_3	LINE	Novel	807	4	4	9	3
Sj_Blaster_Grouper_18907_MAP_3	LINE	SjR2	1264	7	1	5	0
Sj_Blaster_Recon_12702_MAP_3	LINE	Novel	612	3	10	1	4
Sj_Blaster_Recon_10644_MAP_7	LINE	PERERE-4_2p	2228	6	4	6	1
Sj_Blaster_Recon_5711_MAP_8	LINE	PERERE-4_2p	2272	8	5	3	5
Sj_Blaster_Grouper_3699_MAP_3	LINE	Novel	464	5	4	2	6
Sj_Blaster_Recon_4461_MAP_3	LINE	Novel	1447	1	9	3	4
Sj_Blaster_Recon_7702_MAP_3	LINE	SjR2	489	4	6	2	9
Sj_Blaster_Grouper_33154_MAP_3	LINE	PERERE-4_2p	3811	6	4	3	5
Sj_Blaster_Grouper_31012_MAP_3	LINE	RTE-1_AGp	3243	1	6	2	11
Sj_Blaster_Recon_10244_MAP_3	LINE	RT	1326	20	6	0	1
Sj_Blaster_Grouper_18936_MAP_3	LINE	RTE-1_MD_1p	1327	18	5	2	1
Sj_Blaster_Recon_9025_MAP_3	LINE	SjR2	539	3	0	16	3
Sj_Blaster_Grouper_33709_MAP_3	LINE	SjR2	1359	12	6	2	4
Sj_Blaster_Recon_10322_MAP_6	LINE	PERERE-4_2p	1457	1	7	7	2

Sj_Blaster_Grouper_8581_MAP_3	LINE	Novel	605	9	2	7	4
Sj_Blaster_Recon_17063_MAP_3	LINE	SjR2	508	13	6	4	2
Sj_Blaster_Recon_8271_MAP_3	LINE	Novel	1012	4	4	10	0
Sj_Blaster_Recon_15456_MAP_3	LINE	Novel	2099	8	9	1	6
Sj_Blaster_Recon_551_MAP_14	LINE	SjR2	1927	3	3	10	5
Sj_Blaster_Recon_12963_MAP_3	LINE	Novel	1517	2	7	5	3
Sj_Blaster_Recon_1689_MAP_20	LINE	Novel	8402	5	10	11	0
Sj_Blaster_Recon_7680_MAP_4	LINE	RTE-1_MD_1p	1005	2	12	4	5
Sj_Blaster_Recon_2819_MAP_3	LINE	Novel	1289	3	8	4	6
Sj_Blaster_Recon_11174_MAP_3	LINE	Novel	1133	7	5	10	4
Sj_Blaster_Recon_12862_MAP_3	LINE	Novel	466	1	8	10	4
Sj_Blaster_Recon_11449_MAP_7	LINE	SjR2	2569	6	2	1	14
Sj_Blaster_Recon_5699_MAP_14	LINE	Novel	3907	13	2	5	5
Sj_Blaster_Recon_7539_MAP_3	LINE	RTE-1_MD_1p	1550	4	15	2	3
Sj_Blaster_Recon_420_MAP_6	LINE	Novel	1587	5	5	1	12
Sj_Blaster_Grouper_27545_MAP_3	LINE	RTE-1_AGp	3313	3	5	11	6
Sj_Blaster_Recon_18155_MAP_3	LINE	RTE-1_MD_1p	395	25	3	5	1
Sj_Blaster_Recon_13580_MAP_3	LINE	RTE-1_MD_1p	812	10	6	2	7
Sj_Blaster_Recon_13803_MAP_3	LINE	Novel	1301	22	7	4	5
Sj_Blaster_Grouper_4717_MAP_3	LINE	SjR2	492	4	11	2	4
Sj_Blaster_Grouper_16391_MAP_6	LINE	PERERE-4_2p	1066	4	7	9	9
Sj_Blaster_Recon_3150_MAP_6	LINE	SjR2	2960	2	7	11	4
Sj_Blaster_Recon_8470_MAP_3	LINE	SjR2	631	5	13	5	6
Sj_Blaster_Grouper_5827_MAP_3	LINE	RTE-1_MD_1p	524	4	27	4	3
Sj_Blaster_Recon_18117_MAP_4	LINE	SjR2	485	3	29	3	8
Sj_Blaster_Recon_11345_MAP_4	LINE	RTE-1_MD_1p	1237	9	10	6	3
Sj_Blaster_Recon_10392_MAP_7	LINE	RT	3369	0	0	0	26
Sj_Blaster_Recon_17411_MAP_3	LINE	SjR2	224	18	3	2	3
Sj_Blaster_Recon_11446_MAP_3	LINE	Novel	1120	5	5	11	9
Sj_Blaster_Grouper_24334_MAP_5	LINE	SjR2	2190	10	7	14	1
Sj_Blaster_Recon_336_MAP_3	LINE	Novel	2405	6	12	14	1
Sj_Blaster_Grouper_17372_MAP_3	LINE	PERERE-4_2p	1173	6	6	10	10
Sj_Blaster_Grouper_15666_MAP_4	LINE	SjR2	1003	8	5	12	3
Sj_Blaster_Recon_18531_MAP_3	LINE	SjR2	903	6	11	2	13

Sj_Blaster_Recon_2294_MAP_3	LINE	SjR2	1190	12	9	2	6
Sj_Blaster_Recon_1258_MAP_3	LINE	SjR2	1205	20	5	15	8
Sj_Blaster_Recon_6693_MAP_3	LINE	Novel	1575	12	8	4	4
Sj_Blaster_Recon_9020_MAP_4	LINE	SjR2	824	5	22	3	9
Sj_Blaster_Recon_16900_MAP_4	LINE	PERERE-4_2p	1693	4	15	2	5
Sj_Blaster_Grouper_17017_MAP_3	LINE	RTE-1_MD_1p	1131	8	8	12	3
Sj_Blaster_Recon_10471_MAP_6	LINE	RTE-1_AGp	1899	8	22	5	3
Sj_Blaster_Recon_965_MAP_3	LINE	BN000793_1p	839	5	13	4	8
Sj_Blaster_Grouper_15158_MAP_7	LINE	Novel	1014	6	6	12	12
Sj_Blaster_Grouper_18368_MAP_3	LINE	Novel	1288	13	10	5	6
Sj_Blaster_Grouper_18192_MAP_5	LINE	PERERE-4_2p	1272	5	9	10	13
Sj_Blaster_Recon_2142_MAP_4	LINE	Novel	2949	12	9	1	10
Sj_Blaster_Grouper_6704_MAP_3	LINE	SjR2	550	7	18	2	3
Sj_Blaster_Recon_3903_MAP_3	LINE	Novel	1165	7	9	11	5
Sj_Blaster_Recon_16031_MAP_3	LINE	PERERE-4_2p	1216	8	12	6	4
Sj_Blaster_Recon_13829_MAP_4	LINE	Novel	2083	8	8	4	6
Sj_Blaster_Recon_11717_MAP_4	LINE	Novel	2636	11	9	7	7
Sj_Blaster_Recon_4181_MAP_3	LINE	Novel	651	2	25	8	11
Sj_Blaster_Grouper_19068_MAP_3	LINE	PERERE-4_2p	1386	6	9	9	14
Sj_Blaster_Grouper_12519_MAP_3	LINE	PERERE-4_2p	779	13	15	7	4
Sj_Blaster_Grouper_17481_MAP_5	LINE	PERERE-4_2p	1185	6	10	10	13
Sj_Blaster_Recon_700_MAP_3	LINE	SjR2	632	1	21	6	4
Sj_Blaster_Grouper_3246_MAP_3	LINE	SjR2	450	12	13	4	3
Sj_Blaster_Recon_12713_MAP_3	LINE	SjR2	912	5	15	9	10
Sj_Blaster_Recon_12670_MAP_4	LINE	PERERE-4_2p	1690	8	8	3	9
Sj_Blaster_Recon_2293_MAP_3	LINE	Novel	3075	9	9	7	13
Sj_Blaster_Grouper_18889_MAP_8	LINE	PERERE-4_2p	1466	6	11	10	15
Sj_Blaster_Grouper_19040_MAP_7	LINE	PERERE-4_2p	1428	10	6	15	10
Sj_Blaster_Recon_3456_MAP_3	LINE	Novel	1239	9	11	5	7
Sj_Blaster_Grouper_19576_MAP_3	LINE	Novel	1415	40	3	5	8
Sj_Blaster_Recon_4741_MAP_4	LINE	RTE-1_MD_1p	2677	10	14	31	2
Sj_Blaster_Grouper_21342_MAP_3	LINE	SjR2	1528	68	1	5	4
Sj_Blaster_Grouper_26988_MAP_3	LINE	PERERE-4_2p	1151	10	8	4	18
Sj_Blaster_Grouper_23628_MAP_6	LINE	PERERE-4_2p	1150	13	15	10	3

Sj_Blaster_Recon_5408_MAP_3	LINE	RT	1491	5	6	4	27
Sj_Blaster_Recon_7100_MAP_3	LINE	SjR2	1801	7	5	8	12
Sj_Blaster_Recon_8876_MAP_3	LINE	Novel	562	52	10	10	7
Sj_Blaster_Recon_6668_MAP_4	LINE	RTE-1_AGp	2037	4	22	13	17
Sj_Blaster_Recon_14148_MAP_4	LINE	SjR2	676	24	4	5	1
Sj_Blaster_Grouper_28587_MAP_3	LINE	RTE-1_AGp	3526	11	30	9	9
Sj_Blaster_Grouper_31612_MAP_3	LINE	PERERE-4_2p	3212	21	22	5	7
Sj_Blaster_Recon_13695_MAP_3	LINE	RTE-1_MD_1p	694	11	1	8	14
Sj_Blaster_Recon_2611_MAP_3	LINE	Novel	1086	25	5	8	4
Sj_Blaster_Recon_4683_MAP_4	LINE	Novel	3267	20	4	8	16
Sj_Blaster_Recon_4211_MAP_3	LINE	Novel	1218	8	27	12	1
Sj_Blaster_Recon_3556_MAP_7	LINE	Novel	1767	6	14	5	11
Sj_Blaster_Grouper_7525_MAP_3	LINE	SjR2	576	29	4	11	12
Sj_Blaster_Recon_15458_MAP_3	LINE	RTE-1_MD_1p	679	16	11	13	3
Sj_Blaster_Grouper_12695_MAP_3	LINE	Novel	790	10	38	9	1
Sj_Blaster_Recon_12136_MAP_7	LINE	Novel	1900	13	10	12	15
Sj_Blaster_Grouper_28668_MAP_3	LINE	Novel	2058	8	18	11	6
Sj_Blaster_Recon_15353_MAP_3	LINE	RTE-1_MD_1p	1332	20	16	7	4
Sj_Blaster_Grouper_8333_MAP_3	LINE	SjR2	602	8	22	8	3
Sj_Blaster_Recon_7798_MAP_3	LINE	RTE-1_MD_1p	1684	29	17	7	6
Sj_Blaster_Recon_668_MAP_3	LINE	RTE-1_MD_1p	626	9	17	5	9
Sj_Blaster_Recon_1347_MAP_20	LINE	PERERE-4_2p	3911	13	18	4	15
Sj_Blaster_Grouper_7618_MAP_3	LINE	SjR2	580	12	9	3	12
Sj_Blaster_Grouper_11049_MAP_3	LINE	RTE-1_MD_1p	703	15	23	6	7
Sj_Blaster_Grouper_27167_MAP_8	LINE	Novel	2737	15	4	11	15
Sj_Blaster_Recon_8585_MAP_3	LINE	Novel	1020	21	12	6	13
Sj_Blaster_Recon_10283_MAP_3	LINE	SjR2	616	20	24	7	6
Sj_Blaster_Grouper_34212_MAP_3	LINE	PERERE-4_2p	2500	1	21	7	17
Sj_Blaster_Recon_16299_MAP_3	LINE	Novel	2301	20	10	5	25
Sj_Blaster_Grouper_27166_MAP_4	LINE	RTE-1_AGp	2437	2	16	0	34
Sj_Blaster_Recon_1193_MAP_7	LINE	Novel	1416	23	9	18	9
Sj_Blaster_Recon_15833_MAP_3	LINE	RTE-1_MD_1p	1049	14	25	8	7
Sj_Blaster_Grouper_13845_MAP_3	LINE	RTE-1_MD_1p	860	33	35	5	4
Sj_Blaster_Recon_9307_MAP_7	LINE	SjR2	2256	32	7	4	9

Sj_Blaster_Recon_307_MAP_3	LINE	RTE-1_MD_1p	979	30	43	4	15
Sj_Blaster_Grouper_24144_MAP_3	LINE	PERERE-4_2p	2166	12	14	13	14
Sj_Blaster_Recon_5272_MAP_10	LINE	SjR2	4525	23	12	4	18
Sj_Blaster_Grouper_28814_MAP_3	LINE	RTE-1_AGp	1545	19	14	11	18
Sj_Blaster_Recon_12245_MAP_5	LINE	SjR2	2174	9	21	7	24
Sj_Blaster_Recon_15074_MAP_3	LINE	SjR2	713	21	25	8	11
Sj_Blaster_Recon_4697_MAP_4	LINE	PERERE-4_2p	5714	11	10	38	5
Sj_Blaster_Recon_594_MAP_3	LINE	SjR2	1145	18	24	8	5
Sj_Blaster_Recon_11394_MAP_4	LINE	SjR2	1702	24	15	8	9
Sj_Blaster_Recon_9286_MAP_3	LINE	Novel	927	20	14	18	7
Sj_Blaster_Grouper_3759_MAP_3	LINE	SjR2	465	19	11	27	10
Sj_Blaster_Recon_7500_MAP_4	LINE	Novel	1524	37	15	9	5
Sj_Blaster_Recon_9633_MAP_3	LINE	SjR2	1367	9	44	8	5
Sj_Blaster_Recon_5954_MAP_3	LINE	SjR2	865	29	21	13	7
Sj_Blaster_Recon_12628_MAP_3	LINE	SjR2	925	31	30	5	20
Sj_Blaster_Grouper_23871_MAP_3	LINE	PERERE-4_2p	2234	26	21	15	6
Sj_Blaster_Recon_7575_MAP_3	LINE	RTE-1_MD_1p	658	26	23	11	11
Sj_Blaster_Recon_10466_MAP_4	LINE	SjR2	877	33	10	25	2
Sj_Blaster_Grouper_18316_MAP_5	LINE	Novel	1242	25	8	17	18
Sj_Blaster_Recon_7903_MAP_3	LINE	SjR2	805	6	45	4	8
Sj_Blaster_Recon_2033_MAP_3	LINE	SjR2	614	17	18	12	19
Sj_Blaster_Grouper_26216_MAP_3	LINE	PERERE-4_2p	2829	14	24	8	20
Sj_Blaster_Recon_8635_MAP_4	LINE	Novel	1457	23	17	15	14
Sj_Blaster_Grouper_31184_MAP_3	LINE	SjR2	1357	23	31	2	15
Sj_Blaster_Grouper_28842_MAP_3	LINE	Novel	1799	34	23	14	5
Sj_Blaster_Grouper_23616_MAP_6	LINE	PERERE-4_2p	2175	14	24	8	20
Sj_Blaster_Grouper_11699_MAP_4	LINE	Novel	735	13	21	19	14
Sj_Blaster_Recon_12806_MAP_4	LINE	RTE-1_MD_1p	1148	50	15	10	17
Sj_Blaster_Recon_10386_MAP_4	LINE	SjR2	1350	25	18	6	35
Sj_Blaster_Grouper_31172_MAP_4	LINE	SjR2	1318	24	33	2	15
Sj_Blaster_Recon_721_MAP_3	LINE	RTE-1_MD_1p	903	32	4	15	19
Sj_Blaster_Recon_961_MAP_5	LINE	SjR2	2898	26	28	22	8
Sj_Blaster_Recon_2989_MAP_12	LINE	SjR2	4699	28	21	17	5
Sj_Blaster_Recon_2978_MAP_3	LINE	Novel	1843	19	64	9	21

Sj_Blaster_Grouper_32042_MAP_3	LINE	Novel	1276	9	26	21	20
Sj_Blaster_Grouper_13926_MAP_3	LINE	Novel	867	67	15	10	21
Sj_Blaster_Recon_13476_MAP_3	LINE	SjR2	1203	10	29	12	22
Sj_Blaster_Recon_8691_MAP_3	LINE	SjR2	1424	25	21	11	16
Sj_Blaster_Recon_5121_MAP_3	LINE	SjR2	716	33	27	10	7
Sj_Blaster_Grouper_28854_MAP_4	LINE	RTE-1_AGp	3992	34	8	24	14
Sj_Blaster_Recon_7551_MAP_6	LINE	Poseidon_Ap_1p	4895	15	31	19	10
Sj_Blaster_Piler_278.317_MAP_3	LINE	SjR2	387	29	22	13	7
Sj_Blaster_Grouper_27180_MAP_3	LINE	RTE-1_AGp	2195	9	29	20	13
Sj_Blaster_Recon_13864_MAP_3	LINE	RTE-1_AGp	893	28	6	29	16
Sj_Blaster_Recon_11275_MAP_3	LINE	SjR2	1116	31	17	22	24
Sj_Blaster_Recon_10101_MAP_6	LINE	RTE-1_AGp	2988	15	21	11	25
Sj_Blaster_Recon_1194_MAP_7	LINE	Novel	1443	34	18	12	11
Sj_Blaster_Recon_6093_MAP_3	LINE	RTE-1_AGp	1735	13	41	25	19
Sj_Blaster_Recon_2576_MAP_3	LINE	SjR2	666	23	17	27	34
Sj_Blaster_Grouper_28881_MAP_3	LINE	SjR2	1608	26	48	9	20
Sj_Blaster_Recon_14981_MAP_3	LINE	SjR2	1073	18	28	13	31
Sj_Blaster_Grouper_23638_MAP_3	LINE	SjR2	1531	14	34	12	23
Sj_Blaster_Recon_17200_MAP_3	LINE	SjR2	217	34	31	19	5
Sj_Blaster_Recon_456_MAP_20	LINE	Novel	9580	6	27	5	47
Sj_Blaster_Grouper_27290_MAP_3	LINE	RTE-1_MD_1p	2227	12	44	10	35
Sj_Blaster_Grouper_18038_MAP_4	LINE	SjR2	1247	37	18	22	13
Sj_Blaster_Recon_11301_MAP_5	LINE	RTE-1_MD_1p	734	66	15	11	19
Sj_Blaster_Recon_3686_MAP_3	LINE	SjR2	747	43	15	18	28
Sj_Blaster_Recon_12852_MAP_4	LINE	PERERE-4_2p	1616	22	27	13	31
Sj_Blaster_Grouper_24400_MAP_4	LINE	Novel	1197	14	46	18	23
Sj_Blaster_Grouper_24496_MAP_3	LINE	RTE-1_MD_1p	1227	15	45	18	23
Sj_Blaster_Grouper_22802_MAP_3	LINE	SjR2	1559	36	19	28	14
Sj_Blaster_Recon_10733_MAP_3	LINE	SjR2	988	58	40	27	2
Sj_Blaster_Recon_10293_MAP_3	LINE	SjR2	743	28	15	41	39
Sj_Blaster_Grouper_17588_MAP_3	LINE	RTE-1_MD_1p	1196	60	56	11	12
Sj_Blaster_Grouper_27255_MAP_3	LINE	RTE-1_AGp	2048	44	15	21	20
Sj_Blaster_Recon_11347_MAP_4	LINE	SjR2	690	47	42	14	21
Sj_Blaster_Grouper_31679_MAP_3	LINE	SjR2	1225	88	18	23	20

Sj_Blaster_Recon_549_MAP_4	LINE	PERERE-4_2p	1381	35	25	39	26
Sj_Blaster_Recon_2490_MAP_3	LINE	SjR2	1242	47	23	32	18
Sj_Blaster_Grouper_29485_MAP_3	LINE	RTE-1_AGp	2376	15	45	25	21
Sj_Blaster_Recon_553_MAP_4	LINE	SjR2	725	38	42	23	29
Sj_Blaster_Grouper_19687_MAP_3	LINE	SjR2	1200	45	37	25	10
Sj_Blaster_Recon_13830_MAP_7	LINE	PERERE-4_2p	1376	24	25	22	41
Sj_Blaster_Grouper_26728_MAP_6	LINE	Novel	2229	49	17	27	22
Sj_Blaster_Grouper_26699_MAP_3	LINE	Novel	2987	46	17	24	22
Sj_Blaster_Grouper_28442_MAP_3	LINE	PERERE-4_2p	2794	50	42	13	25
Sj_Blaster_Recon_5955_MAP_12	LINE	RTE-1_MD_1p	3213	39	39	34	23
Sj_Blaster_Recon_12988_MAP_3	LINE	RTE-1_MD_1p	1501	49	63	5	28
Sj_Blaster_Recon_5565_MAP_16	LINE	SjR2	5772	38	37	11	48
Sj_Blaster_Grouper_27272_MAP_4	LINE	Novel	3164	73	16	34	53
Sj_Blaster_Grouper_26142_MAP_3	LINE	RTE-1_AGp	2226	58	21	28	20
Sj_Blaster_Grouper_28342_MAP_3	LINE	RTE-1_AGp	2794	17	49	24	29
Sj_Blaster_Grouper_12560_MAP_20	LINE	PENELOPE_1p	918	26	52	23	25
Sj_Blaster_Recon_16550_MAP_3	LINE	RTE-1_MD_1p	747	27	38	23	59
Sj_Blaster_Recon_7577_MAP_5	LINE	Novel	4501	45	27	40	16
Sj_Blaster_Grouper_6059_MAP_3	LINE	SjR2	530	45	42	11	26
Sj_Blaster_Recon_7968_MAP_4	LINE	Novel	3673	47	29	30	26
Sj_Blaster_Recon_9289_MAP_3	LINE	Novel	1307	49	48	28	22
Sj_Blaster_Grouper_17290_MAP_3	LINE	SjR2	966	88	25	16	13
Sj_Blaster_Recon_17676_MAP_4	LINE	SjR2	309	60	30	44	23
Sj_Blaster_Recon_919_MAP_8	LINE	RTE-1_MD_1p	2290	60	58	15	17
Sj_Blaster_Recon_14895_MAP_4	LINE	SjR2	735	35	48	25	16
Sj_Blaster_Recon_9611_MAP_4	LINE	Novel	1435	35	67	23	23
Sj_Blaster_Recon_3236_MAP_5	LINE	Novel	1572	48	67	28	18
Sj_Blaster_Grouper_15045_MAP_3	LINE	RTE-1_AGp	949	15	64	23	49
Sj_Blaster_Grouper_6925_MAP_3	LINE	SjR2	556	56	50	20	16
Sj_Blaster_Recon_8807_MAP_6	LINE	PENELOPE_1p	1857	18	49	32	47
Sj_Blaster_Recon_4974_MAP_3	LINE	SjR2	649	29	49	26	37
Sj_Blaster_Recon_1535_MAP_3	LINE	Novel	1333	43	55	18	33
Sj_Blaster_Recon_13358_MAP_3	LINE	SjR2	1026	49	68	22	12
Sj_Blaster_Recon_3984_MAP_3	LINE	RTE-1_MD_1p	1339	65	100	14	19

Sj_Blaster_Recon_2779_MAP_3	LINE	SjR2	915	40	56	21	20
Sj_Blaster_Recon_4856_MAP_3	LINE	SjR2	944	33	66	35	17
Sj_Blaster_Recon_8160_MAP_4	LINE	PERERE-4_2p	1616	48	62	23	80
Sj_Blaster_Recon_3619_MAP_4	LINE	RTE-1_MD_1p	1645	15	110	45	30
Sj_Blaster_Recon_4526_MAP_3	LINE	RTE-1_AGp	792	17	79	90	25
Sj_Blaster_Recon_3584_MAP_13	LINE	CR1-2_AG-ORF2p	4424	29	60	27	32
Sj_Blaster_Grouper_27380_MAP_4	LINE	RTE-1_AGp	3235	71	20	40	22
Sj_Blaster_Recon_2513_MAP_3	LINE	Novel	1137	69	59	21	17
Sj_Blaster_Recon_3014_MAP_12	LINE	SjR2	4136	46	49	31	22
Sj_Blaster_Grouper_30732_MAP_7	LINE	PERERE-4_2p	1454	69	60	27	28
Sj_Blaster_Grouper_31672_MAP_3	LINE	RTE-1_MD_1p	1963	26	74	25	33
Sj_Blaster_Recon_8742_MAP_4	LINE	Novel	1364	42	53	29	40
Sj_Blaster_Grouper_29284_MAP_5	LINE	RTE-1_AGp	3927	20	73	31	32
Sj_Blaster_Grouper_25204_MAP_3	LINE	RTE-1_MD_1p	2172	30	72	26	42
Sj_Blaster_Recon_4130_MAP_3	LINE	RTE-1_MD_1p	1185	84	36	22	35
Sj_Blaster_Recon_669_MAP_5	LINE	Novel	3575	68	76	22	23
Sj_Blaster_Recon_3736_MAP_3	LINE	RTE-1_MD_1p	1094	53	54	30	35
Sj_Blaster_Recon_13357_MAP_3	LINE	Novel	1024	63	101	30	33
Sj_Blaster_Grouper_33189_MAP_3	LINE	Novel	4271	71	26	20	55
Sj_Blaster_Grouper_33021_MAP_3	LINE	Novel	7180	71	26	34	41
Sj_Blaster_Grouper_17465_MAP_4	LINE	RTE-1_MD_1p	1183	21	125	33	38
Sj_Blaster_Grouper_29057_MAP_9	LINE	TART_DV_2p	4446	64	20	49	31
Sj_Blaster_Grouper_34699_MAP_3	LINE	RTE-1_MD_1p	9857	18	126	34	37
Sj_Blaster_Recon_17142_MAP_6	LINE	SjR2	230	64	39	34	22
Sj_Blaster_Recon_1879_MAP_6	LINE	Novel	1562	66	65	36	33
Sj_Blaster_Recon_4048_MAP_3	LINE	RTE-1_MD_1p	939	25	91	39	22
Sj_Blaster_Recon_12933_MAP_3	LINE	RT	1287	92	38	36	33
Sj_Blaster_Recon_13509_MAP_3	LINE	Novel	2710	49	65	20	29
Sj_Blaster_Recon_2634_MAP_5	LINE	Novel	1460	35	38	89	38
Sj_Blaster_Recon_9423_MAP_4	LINE	Novel	4181	66	66	59	19
Sj_Blaster_Recon_165_MAP_5	LINE	Novel	1825	39	67	43	46
Sj_Blaster_Grouper_12384_MAP_3	LINE	SjR2	770	67	53	40	12
Sj_Blaster_Grouper_10460_MAP_3	LINE	SjR2	678	50	71	19	50
Sj_Blaster_Grouper_22671_MAP_6	LINE	RTE-1_MD_1p	1984	41	150	40	23

Sj_Blaster_Recon_10323_MAP_3	LINE	Novel	653	42	80	28	45
Sj_Blaster_Grouper_30587_MAP_3	LINE	SjR2	937	33	62	31	77
Sj_Blaster_Grouper_28343_MAP_4	LINE	RTE-1_AGp	3686	21	83	33	51
Sj_Blaster_Grouper_33987_MAP_4	LINE	RTE-1_AGp	3718	23	78	32	61
Sj_Blaster_Recon_9301_MAP_3	LINE	Novel	1868	62	59	29	53
Sj_Blaster_Grouper_29389_MAP_7	LINE	TART_DV_2p	4716	71	36	64	36
Sj_Blaster_Recon_3015_MAP_3	LINE	SjR2	1206	41	93	25	50
Sj_Blaster_Grouper_27268_MAP_3	LINE	PERERE-4_2p	2958	41	54	64	48
Sj_Blaster_Recon_9936_MAP_3	LINE	SjR2	766	107	66	22	40
Sj_Blaster_Grouper_14177_MAP_5	LINE	PERERE-4_2p	882	85	70	41	35
Sj_Blaster_Recon_16803_MAP_3	LINE	RTE-1_MD_1p	796	46	119	59	51
Sj_Blaster_Grouper_6686_MAP_3	LINE	SjR2	551	40	87	21	71
Sj_Blaster_Recon_2206_MAP_3	LINE	SjR2	1154	83	91	16	63
Sj_Blaster_Recon_4104_MAP_7	LINE	SjR2	2307	64	75	65	20
Sj_Blaster_Recon_4884_MAP_3	LINE	SjR2	679	63	44	69	33
Sj_Blaster_Grouper_5884_MAP_3	LINE	SjR2	526	69	104	45	48
Sj_Blaster_Recon_12281_MAP_4	LINE	PERERE-5_2p	2119	72	172	26	57
Sj_Blaster_Grouper_9345_MAP_7	LINE	Novel	637	73	76	31	61
Sj_Blaster_Recon_3552_MAP_4	LINE	RTE-1_MD_1p	1096	38	79	39	92
Sj_Blaster_Recon_1017_MAP_4	LINE	Novel	1347	66	113	23	53
Sj_Blaster_Grouper_28006_MAP_4	LINE	RTE-1_MD_1p	3496	88	48	65	40
Sj_Blaster_Recon_4394_MAP_3	LINE	Novel	1334	45	62	55	99
Sj_Blaster_Recon_9795_MAP_17	LINE	PERERE-4_2p	5535	51	60	64	62
Sj_Blaster_Recon_14308_MAP_4	LINE	Novel	2244	80	31	82	37
Sj_Blaster_Recon_13689_MAP_4	LINE	Novel	1512	61	49	78	36
Sj_Blaster_Recon_10290_MAP_3	LINE	SjR2	536	68	63	30	95
Sj_Blaster_Recon_5744_MAP_3	LINE	RTE-1_AGp	1203	56	75	41	62
Sj_Blaster_Recon_13068_MAP_3	LINE	Novel	878	79	93	74	24
Sj_Blaster_Recon_5953_MAP_4	LINE	SjR2	2081	102	65	68	47
Sj_Blaster_Recon_335_MAP_3	LINE	Novel	1021	92	50	52	64
Sj_Blaster_Recon_1370_MAP_7	LINE	Novel	2370	75	99	52	45
Sj_Blaster_Recon_752_MAP_15	LINE	Novel	3567	65	99	36	51
Sj_Blaster_Grouper_2347_MAP_3	LINE	SjR2	423	169	72	28	37
Sj_Blaster_Recon_4491_MAP_3	LINE	Novel	1452	70	85	65	41

Sj_Blaster_Recon_2228_MAP_4	LINE	Novel	1264	44	212	30	20
Sj_Blaster_Grouper_22616_MAP_3	LINE	Poseidon_Ap_1p	874	80	99	49	53
Sj_Blaster_Recon_6141_MAP_8	LINE	Novel	2510	85	71	85	25
Sj_Blaster_Grouper_10375_MAP_4	LINE	SjR2	677	111	61	35	42
Sj_Blaster_Grouper_2804_MAP_3	LINE	Novel	437	115	63	43	107
Sj_Blaster_Grouper_9889_MAP_3	LINE	SjR2	673	36	139	62	46
Sj_Blaster_Recon_12477_MAP_3	LINE	SjR2	1476	63	127	23	81
Sj_Blaster_Recon_16538_MAP_3	LINE	SjR2	907	142	135	50	42
Sj_Blaster_Recon_3502_MAP_3	LINE	Novel	1229	89	161	52	43
Sj_Blaster_Recon_17053_MAP_3	LINE	RTE-1_MD_1p	1390	81	133	57	61
Sj_Blaster_Recon_4846_MAP_4	LINE	Novel	1158	67	67	105	28
Sj_Blaster_Recon_6057_MAP_4	LINE	RTE-1_MD_1p	1797	107	81	58	53
Sj_Blaster_Recon_11571_MAP_3	LINE	Novel	2482	126	118	74	54
Sj_Blaster_Grouper_18181_MAP_9	LINE	Novel	1269	9	54	4	240
Sj_Blaster_Recon_17318_MAP_3	LINE	Novel	571	120	83	66	39
Sj_Blaster_Grouper_11174_MAP_4	LINE	SjR2	710	49	113	59	69
Sj_Blaster_Grouper_21039_MAP_5	LINE	Poseidon_Ap_1p	994	102	83	95	53
Sj_Blaster_Recon_8586_MAP_3	LINE	PERERE-5_2p	920	81	128	60	69
Sj_Blaster_Grouper_14656_MAP_3	LINE	Novel	919	53	109	55	130
Sj_Blaster_Grouper_6793_MAP_20	LINE	Novel	556	104	71	85	104
Sj_Blaster_Recon_3324_MAP_3	LINE	SjR2	1183	176	101	47	67
Sj_Blaster_Grouper_20160_MAP_3	LINE	Novel	1229	75	172	43	89
Sj_Blaster_Grouper_18184_MAP_3	LINE	SjR2	1270	78	106	83	63
Sj_Blaster_Piler_39.0_MAP_20	LINE	SjR2	646	139	84	40	73
Sj_Blaster_Grouper_34735_MAP_3	LINE	PERERE-4_2p	7873	3	172	8	168
Sj_Blaster_Recon_2572_MAP_4	LINE	RTE-1_AGp	1676	111	73	116	72
Sj_Blaster_Grouper_26670_MAP_3	LINE	Poseidon_Ap_1p	1223	92	97	104	68
Sj_Blaster_Recon_10188_MAP_3	LINE	Novel	1050	141	83	57	60
Sj_Blaster_Recon_17113_MAP_3	LINE	RTE-1_AGp	393	197	98	95	69
Sj_Blaster_Grouper_18895_MAP_3	LINE	RTE-1_MD_1p	1361	187	89	54	65
Sj_Blaster_Recon_5096_MAP_3	LINE	RTE-1_AGp	957	113	137	51	73
Sj_Blaster_Grouper_31041_MAP_3	LINE	Novel	1830	174	110	69	56
Sj_Blaster_Grouper_14488_MAP_3	LINE	SjR2	906	128	77	86	57
Sj_Blaster_Grouper_19480_MAP_4	LINE	Poseidon_Ap_1p	1453	67	166	77	78

Sj_Blaster_Recon_1297_MAP_3	LINE	Novel	1769	104	138	40	99
Sj_Blaster_Grouper_6983_MAP_3	LINE	Novel	559	77	131	58	133
Sj_Blaster_Grouper_19419_MAP_6	LINE	RTE-1_MD_1p	1228	161	166	86	71
Sj_Blaster_Recon_15966_MAP_3	LINE	Novel	1554	149	104	87	53
Sj_Blaster_Grouper_20408_MAP_3	LINE	Novel	1162	97	114	131	75
Sj_Blaster_Grouper_20673_MAP_3	LINE	RTE-1_AGp	1613	128	104	67	79
Sj_Blaster_Recon_13141_MAP_3	LINE	SjR2	572	118	168	142	54
Sj_Blaster_Grouper_6622_MAP_3	LINE	SjR2	548	196	178	51	85
Sj_Blaster_Grouper_6293_MAP_20	LINE	Novel	715	110	109	77	137
Sj_Blaster_Grouper_11659_MAP_3	LINE	Novel	736	90	168	31	136
Sj_Blaster_Grouper_29814_MAP_5	LINE	Poseidon_Ap_1p	1251	117	117	77	109
Sj_Blaster_Recon_5628_MAP_4	LINE	Novel	1406	123	127	51	104
Sj_Blaster_Recon_17393_MAP_3	LINE	SjR2	930	180	146	48	93
Sj_Blaster_Recon_355_MAP_3	LINE	PERERE-4_2p	747	59	255	74	78
Sj_Blaster_Grouper_17176_MAP_15	LINE	Poseidon_Ap_1p	1178	141	97	104	113
Sj_Blaster_Grouper_27134_MAP_3	LINE	RTE-1_MD_1p	2020	230	164	77	107
Sj_Blaster_Recon_5629_MAP_3	LINE	Novel	2018	142	137	72	89
Sj_Blaster_Grouper_25496_MAP_11	LINE	RT	1305	163	93	113	103
Sj_Blaster_Recon_12916_MAP_4	LINE	PERERE-4_2p	1031	178	184	95	57
Sj_Blaster_Grouper_8735_MAP_7	LINE	SjR2	635	126	147	109	91
Sj_Blaster_Grouper_30085_MAP_3	LINE	SjR2	1079	214	182	61	70
Sj_Blaster_Recon_12898_MAP_3	LINE	Novel	1248	172	156	87	89
Sj_Blaster_Recon_15144_MAP_3	LINE	Novel	1061	178	242	61	120
Sj_Blaster_Recon_6155_MAP_3	LINE	Novel	1459	97	152	54	145
Sj_Blaster_Recon_640_MAP_20	LINE	Novel	1300	162	109	76	88
Sj_Blaster_Recon_4050_MAP_7	LINE	Novel	1619	109	128	174	72
Sj_Blaster_Grouper_19256_MAP_16	LINE	Penelope2_Dw_1p	1423	117	113	95	135
Sj_Blaster_Recon_2973_MAP_5	LINE	Novel	1911	148	146	139	66
Sj_Blaster_Grouper_30431_MAP_3	LINE	SjR2	1248	133	261	129	46
Sj_Blaster_Grouper_8012_MAP_3	LINE	SjR2	591	246	154	61	109
Sj_Blaster_Grouper_30747_MAP_3	LINE	RTE-1_MD_1p	1361	230	211	46	100
Sj_Blaster_Recon_10424_MAP_9	LINE	RTE-1_MD_1p	845	211	230	101	46
Sj_Blaster_Recon_6059_MAP_7	LINE	RTE-1_AGp	3261	106	196	76	143
Sj_Blaster_Grouper_1218_MAP_15	LINE	Novel	387	125	179	98	103

Sj_Blaster_Recon_7663_MAP_4	LINE	Novel	2244	143	162	104	71
Sj_Blaster_Grouper_30802_MAP_3	LINE	Novel	1016	98	166	54	150
Sj_Blaster_Grouper_20319_MAP_3	LINE	Novel	1579	185	100	124	87
Sj_Blaster_Grouper_16703_MAP_4	LINE	RT	1102	188	109	108	141
Sj_Blaster_Grouper_19108_MAP_3	LINE	SjR2	1207	160	217	113	57
Sj_Blaster_Grouper_25908_MAP_6	LINE	SjR2	1508	222	238	89	142
Sj_Blaster_Grouper_25261_MAP_6	LINE	Poseidon_Ap_1p	1668	293	87	96	148
Sj_Blaster_Grouper_18734_MAP_10	LINE	Poseidon_Ap_1p	1377	169	119	111	134
Sj_Blaster_Grouper_13207_MAP_4	LINE	Novel	818	198	247	100	67
Sj_Blaster_Grouper_26547_MAP_3	LINE	SjR2	2613	243	230	142	87
Sj_Blaster_Recon_18300_MAP_3	LINE	Novel	1583	257	137	155	56
Sj_Blaster_Recon_5087_MAP_5	LINE	PERERE-4_2p	1192	211	111	89	124
Sj_Blaster_Grouper_12491_MAP_3	LINE	SjR2	779	171	138	103	139
Sj_Blaster_Grouper_20739_MAP_3	LINE	Novel	1649	264	136	160	53
Sj_Blaster_Recon_308_MAP_20	LINE	Poseidon_Ap_1p	2112	130	191	141	91
Sj_Blaster_Recon_4152_MAP_3	LINE	RTE-1_AGp	1201	120	177	66	182
Sj_Blaster_Recon_7885_MAP_3	LINE	SjR2	1925	161	232	77	143
Sj_Blaster_Grouper_24791_MAP_4	LINE	RTE-1_MD_1p	1525	284	226	95	148
Sj_Blaster_Grouper_15871_MAP_3	LINE	Novel	1021	136	182	63	161
Sj_Blaster_Grouper_24582_MAP_6	LINE	RTE-1_MD_1p	1927	247	243	99	163
Sj_Blaster_Grouper_18801_MAP_3	LINE	Poseidon_Ap_1p	1342	132	185	167	111
Sj_Blaster_Grouper_20038_MAP_3	LINE	RTE-1_MD_1p	1528	266	212	86	117
Sj_Blaster_Recon_4399_MAP_4	LINE	PERERE-4_2p	1098	312	171	70	121
Sj_Blaster_Grouper_16181_MAP_4	LINE	SjR2	1081	223	203	117	86
Sj_Blaster_Grouper_21526_MAP_4	LINE	RTE-1_MD_1p	1782	209	291	174	90
Sj_Blaster_Recon_77_MAP_20	LINE	SjR2	6070	141	236	124	81
Sj_Blaster_Grouper_29043_MAP_3	LINE	Novel	3255	190	183	176	121
Sj_Blaster_Recon_128_MAP_20	LINE	PERERE-4_1p	2843	190	208	224	143
Sj_Blaster_Recon_3224_MAP_11	LINE	Novel	3004	167	220	79	135
Sj_Blaster_Grouper_25233_MAP_3	LINE	Novel	1232	163	218	142	96
Sj_Blaster_Recon_4401_MAP_4	LINE	PERERE-4_2p	943	195	288	93	118
Sj_Blaster_Grouper_23015_MAP_5	LINE	RTE-1_MD_1p	1723	291	250	102	168
Sj_Blaster_Recon_11532_MAP_4	LINE	RTE-1_MD_1p	2585	266	394	70	67
Sj_Blaster_Grouper_31111_MAP_3	LINE	RTE-1_AGp	1234	253	193	145	100

Sj_Blaster_Grouper_18739_MAP_7	LINE	RTE-1_MD_1p	1458	293	252	102	167
Sj_Blaster_Grouper_24433_MAP_3	LINE	RTE-1_MD_1p	2320	253	298	167	103
Sj_Blaster_Grouper_30726_MAP_3	LINE	RTE-1_MD_1p	2637	298	228	102	178
Sj_Blaster_Grouper_32159_MAP_4	LINE	Novel	1160	183	211	157	72
Sj_Blaster_Recon_1024_MAP_12	LINE	PERERE-4_2p	4515	324	351	58	149
Sj_Blaster_Recon_15658_MAP_4	LINE	RTE-1_AGp	2974	257	186	157	75
Sj_Blaster_Grouper_23946_MAP_4	LINE	RTE-1_MD_1p	2256	233	291	192	107
Sj_Blaster_Grouper_23968_MAP_4	LINE	RTE-1_MD_1p	1243	166	337	143	147
Sj_Blaster_Grouper_32050_MAP_4	LINE	SjR2	858	199	300	210	88
Sj_Blaster_Grouper_31436_MAP_3	LINE	SjR2	957	204	267	211	73
Sj_Blaster_Grouper_26035_MAP_3	LINE	RT	1846	185	226	190	140
Sj_Blaster_Recon_11756_MAP_3	LINE	Novel	1227	254	375	86	49
Sj_Blaster_Grouper_17961_MAP_3	LINE	Novel	1250	165	217	127	184
Sj_Blaster_Grouper_18158_MAP_3	LINE	SjR2	1259	166	297	194	104
Sj_Blaster_Grouper_20756_MAP_3	LINE	SjR2	1653	220	287	190	79
Sj_Blaster_Grouper_21980_MAP_20	LINE	PERERE-4_1p	1912	182	299	131	141
Sj_Blaster_Grouper_30374_MAP_3	LINE	Novel	1085	278	167	168	131
Sj_Blaster_Grouper_31963_MAP_3	LINE	SjR2	1042	225	281	153	105
Sj_Blaster_Piler_307.0_MAP_20	LINE	SjR2	495	273	271	126	172
Sj_Blaster_Grouper_22526_MAP_3	LINE	RTE-1_MD_1p	1962	312	268	121	201
Sj_Blaster_Grouper_33853_MAP_3	LINE	RTE-1_MD_1p	1209	198	373	101	130
Sj_Blaster_Grouper_31262_MAP_3	LINE	SjR2	864	338	212	101	204
Sj_Blaster_Grouper_15603_MAP_3	LINE	Novel	996	205	256	88	206
Sj_Blaster_Grouper_21105_MAP_7	LINE	Novel	1741	303	204	122	141
Sj_Blaster_Grouper_33104_MAP_4	LINE	SjR2	997	226	345	221	106
Sj_Blaster_Grouper_26545_MAP_3	LINE	Novel	1415	455	324	82	104
Sj_Blaster_Grouper_30767_MAP_3	LINE	Novel	1301	316	344	92	180
Sj_Blaster_Grouper_32737_MAP_4	LINE	Novel	1122	217	266	99	174
Sj_Blaster_Grouper_9135_MAP_3	LINE	SjR2	631	356	265	141	229
Sj_Blaster_Grouper_21560_MAP_3	LINE	RT	1757	181	257	244	191
Sj_Blaster_Grouper_31411_MAP_3	LINE	SjR2	1121	313	375	160	144
Sj_Blaster_Grouper_32176_MAP_3	LINE	SjR2	1030	297	391	132	161
Sj_Blaster_Grouper_32175_MAP_3	LINE	SjR2	1065	297	398	159	113
Sj_Blaster_Piler_824.0_MAP_3	LINE	SjR2	823	173	346	147	185

Sj_Blaster_Grouper_17616_MAP_3	LINE	RTE-1_MD_1p	1202	431	248	137	163
Sj_Blaster_Grouper_25070_MAP_6	LINE	RT	2561	411	168	200	248
Sj_Blaster_Recon_5663_MAP_11	LINE	RTE-1_MD_1p	926	338	348	194	108
Sj_Blaster_Grouper_8971_MAP_8	LINE	SjR2	645	314	349	112	175
Sj_Blaster_Grouper_15010_MAP_4	LINE	RTE-1_MD_1p	949	387	276	120	255
Sj_Blaster_Grouper_31293_MAP_3	LINE	SjR2	1321	324	301	184	189
Sj_Blaster_Grouper_29456_MAP_3	LINE	SjR2	2780	181	355	185	176
Sj_Blaster_Grouper_21057_MAP_3	LINE	RTE-1_AGp	1309	341	339	154	144
Sj_Blaster_Grouper_32533_MAP_4	LINE	SjR2	1025	346	363	120	181
Sj_Blaster_Grouper_10927_MAP_4	LINE	Novel	700	266	278	252	108
Sj_Blaster_Grouper_16043_MAP_3	LINE	SjR2	1035	460	256	113	213
Sj_Blaster_Grouper_32180_MAP_4	LINE	Novel	852	386	248	138	250
Sj_Blaster_Grouper_32489_MAP_3	LINE	SjR2	852	246	363	286	119
Sj_Blaster_Grouper_23683_MAP_3	LINE	RT	2194	211	305	267	197
Sj_Blaster_Grouper_31438_MAP_3	LINE	SjR2	1355	275	419	159	132
Sj_Blaster_Grouper_23989_MAP_6	LINE	RT	2272	176	438	308	197
Sj_Blaster_Piler_4.0_MAP_20	LINE	RTE-1_MD_1p	1004	445	251	180	132
Sj_Blaster_Grouper_27333_MAP_4	LINE	RT	2447	194	326	252	208
Sj_Blaster_Grouper_24279_MAP_3	LINE	RT	2322	210	296	269	202
Sj_Blaster_Recon_3347_MAP_11	LINE	Novel	3478	365	262	169	164
Sj_Blaster_Piler_17.0_MAP_3	LINE	SjR2	1081	315	345	238	120
Sj_Blaster_Grouper_27325_MAP_3	LINE	Novel	953	347	400	241	198
Sj_Blaster_Grouper_32481_MAP_3	LINE	SjR2	989	403	361	120	182
Sj_Blaster_Grouper_33399_MAP_3	LINE	Novel	1242	218	329	152	254
Sj_Blaster_Grouper_33219_MAP_5	LINE	SjR2	1039	336	340	212	139
Sj_Blaster_Grouper_30443_MAP_3	LINE	Novel	1305	394	236	152	229
Sj_Blaster_Recon_13175_MAP_4	LINE	Novel	2597	356	434	156	204
Sj_Blaster_Grouper_30399_MAP_3	LINE	RTE-1_MD_1p	2129	371	331	177	184
Sj_Blaster_Grouper_33949_MAP_5	LINE	SjR2	1179	415	497	214	123
Sj_Blaster_Grouper_11366_MAP_3	LINE	SjR2	717	338	364	188	236
Sj_Blaster_Grouper_31298_MAP_5	LINE	SjR2	978	394	453	194	140
Sj_Blaster_Recon_2434_MAP_15	LINE	PERERE-4_2p	3002	392	276	245	328
Sj_Blaster_Grouper_30927_MAP_3	LINE	SjR2	1017	389	399	220	105
Sj_Blaster_Grouper_31400_MAP_3	LINE	Novel	1391	318	288	220	194

Sj_Blaster_Grouper_31379_MAP_5	LINE	SjR2	1107	324	478	149	204	
Sj_Blaster_Grouper_26150_MAP_3	LINE	PERERE-4_2p	1237	334	434	192	174	
Sj_Blaster_Grouper_32730_MAP_5	LINE	RTE-1_MD_1p	1261	329	595	152	142	
Sj_Blaster_Grouper_33272_MAP_3	LINE	SjR2	898	460	350	143	245	
Sj_Blaster_Grouper_31915_MAP_3	LINE	RT	1179	286	525	279	139	
Sj_Blaster_Grouper_33387_MAP_4	LINE	SjR2	1161	407	406	205	203	
Sj_Blaster_Grouper_33056_MAP_3	LINE	Novel	1379	351	256	346	164	
Sj_Blaster_Grouper_32023_MAP_3	LINE	Novel	1149	448	361	207	202	
Sj_Blaster_Grouper_34322_MAP_3	LINE	RTE-1_MD_1p	1589	528	308	210	155	
Sj_Blaster_Grouper_32919_MAP_5	LINE	SjR2	998	437	414	205	165	
Sj_Blaster_Grouper_33845_MAP_4	LINE	SjR2	1109	442	429	225	134	
Sj_Blaster_Recon_6453_MAP_4	LINE	Novel	2713	332	382	173	254	
Sj_Blaster_Grouper_29611_MAP_6	LINE	Novel	1078	334	451	185	191	
Sj_Blaster_Recon_2695_MAP_8	LINE	RTE-1_MD_1p	1216	428	398	170	262	
Sj_Blaster_Grouper_31876_MAP_4	LINE	SjR2	954	460	369	165	226	
Sj_Blaster_Recon_8128_MAP_4	LINE	Novel	1428	509	385	200	225	
Sj_Blaster_Grouper_33523_MAP_3	LINE	SjR2	1073	356	485	262	172	
Sj_Blaster_Grouper_32894_MAP_3	LINE	Novel	1377	282	525	135	269	
Sj_Blaster_Grouper_32751_MAP_3	LINE	SjR2	1084	576	333	139	241	
Sj_Blaster_Grouper_33917_MAP_4	LINE	Novel	1141	445	412	190	198	
Sj_Blaster_Grouper_22193_MAP_3	LINE	Novel	1713	334	456	212	192	
Sj_Blaster_Grouper_32025_MAP_4	LINE	Novel	1050	448	422	198	217	
Sj_Blaster_Grouper_31865_MAP_4	LINE	SjR2	1042	499	405	174	231	
Sj_Blaster_Grouper_33616_MAP_3	LINE	RTE-1_MD_1p	1462	499	342	214	235	
Sj_Blaster_Grouper_32659_MAP_4	LINE	SjR2	916	345	554	290	138	
Sj_Blaster_Grouper_32874_MAP_3	LINE	SjR2	996	485	420	244	169	
Sj_Blaster_Grouper_34500_MAP_5	LINE	RTE-1_MD_1p	1459	338	517	159	243	
Sj_Blaster_Grouper_33847_MAP_3	LINE	SjR2	1185	369	540	239	265	
Sj_Blaster_Grouper_25109_MAP_9	LINE	Novel	2616	541	328	181	304	
Sj_Blaster_Grouper_29978_MAP_3	LINE	Novel	1051	528	411	194	246	
Sj_Blaster_Grouper_19289_MAP_3	LINE	Novel	1418	413	408	277	237	
Sj_Blaster_Grouper_33705_MAP_3	LINE	SjR2	1048	498	300	151	363	
Sj_Blaster_Grouper_32617_MAP_3	LINE	SjR2	1120	546	337	177	264	
Sj_Blaster_Grouper_21170_MAP_20	LINE	Novel	1044	357	443	195	268	

Sj_Blaster_Grouper_30361_MAP_4	LINE	Novel	2590	394	324	363	144
Sj_Blaster_Grouper_33829_MAP_4	LINE	RTE-1_AGp	988	409	370	281	212
Sj_Blaster_Grouper_33875_MAP_3	LINE	SjR2	1093	355	533	205	205
Sj_Blaster_Grouper_32118_MAP_3	LINE	SjR2	952	572	382	173	268
Sj_Blaster_Grouper_32070_MAP_18	LINE	Novel	1097	451	461	170	257
Sj_Blaster_Grouper_31417_MAP_3	LINE	SjR2	969	487	459	182	243
Sj_Blaster_Grouper_28058_MAP_4	LINE	Novel	1200	249	428	201	324
Sj_Blaster_Grouper_30468_MAP_3	LINE	Novel	1389	359	518	240	214
Sj_Blaster_Grouper_32670_MAP_4	LINE	SjR2	968	554	382	163	296
Sj_Blaster_Grouper_30141_MAP_3	LINE	RTE-1_MD_1p	1308	619	376	138	271
Sj_Blaster_Grouper_32098_MAP_3	LINE	RTE-1_MD_1p	1145	501	423	178	279
Sj_Blaster_Grouper_28300_MAP_4	LINE	SjR2	1060	512	440	196	242
Sj_Blaster_Grouper_32912_MAP_9	LINE	Novel	1146	439	474	232	206
Sj_Blaster_Grouper_33450_MAP_5	LINE	Novel	1128	396	345	338	171
Sj_Blaster_Grouper_18941_MAP_20	LINE	Novel	1260	465	453	327	154
Sj_Blaster_Grouper_32237_MAP_4	LINE	SjR2	1066	450	499	282	198
Sj_Blaster_Grouper_32389_MAP_3	LINE	Novel	1517	442	286	332	185
Sj_Blaster_Grouper_32658_MAP_5	LINE	RTE-1_MD_1p	1433	704	359	217	183
Sj_Blaster_Grouper_30526_MAP_12	LINE	SjR2	978	547	410	177	335
Sj_Blaster_Grouper_31179_MAP_3	LINE	SjR2	1049	519	583	286	182
Sj_Blaster_Grouper_31074_MAP_3	LINE	SjR2	1110	329	642	290	171
Sj_Blaster_Grouper_32580_MAP_3	LINE	Novel	1224	483	502	221	232
Sj_Blaster_Grouper_33849_MAP_3	LINE	PERERE-4_2p	1104	407	559	325	187
Sj_Blaster_Grouper_33569_MAP_3	LINE	Novel	1445	409	595	243	190
Sj_Blaster_Grouper_10003_MAP_3	LINE	SjR2	665	611	322	191	277
Sj_Blaster_Grouper_33763_MAP_3	LINE	SjR2	1196	481	647	163	249
Sj_Blaster_Grouper_26619_MAP_3	LINE	Novel	1262	425	577	164	256
Sj_Blaster_Grouper_31788_MAP_3	LINE	SjR2	1005	405	663	288	185
Sj_Blaster_Grouper_33342_MAP_4	LINE	RTE-1_MD_1p	1242	650	467	253	183
Sj_Blaster_Grouper_14313_MAP_20	LINE	RTE-1_AGp	1160	427	551	190	334
Sj_Blaster_Grouper_29448_MAP_4	LINE	SjR2	1493	565	448	241	307
Sj_Blaster_Grouper_14297_MAP_20	LINE	Novel	1090	578	470	320	150
Sj_Blaster_Grouper_32612_MAP_3	LINE	Novel	1024	462	532	157	340
Sj_Blaster_Grouper_34304_MAP_3	LINE	Novel	1712	407	382	287	300

Sj_Blaster_Grouper_28871_MAP_5	LINE	SjR2	1119	571	601	289	213
Sj_Blaster_Recon_319_MAP_3	LINE	RTE-1_AGp	1216	415	741	199	294
Sj_Blaster_Grouper_29055_MAP_9	LINE	SjR2	1022	599	471	237	263
Sj_Blaster_Grouper_31615_MAP_4	LINE	RTE-1_MD_1p	1137	507	668	209	322
Sj_Blaster_Grouper_31795_MAP_3	LINE	Novel	1453	512	467	311	197
Sj_Blaster_Grouper_32403_MAP_3	LINE	Novel	1235	439	541	213	291
Sj_Blaster_Grouper_31418_MAP_3	LINE	Novel	1099	410	568	270	345
Sj_Blaster_Grouper_32731_MAP_3	LINE	RTE-1_MD_1p	1535	401	780	199	264
Sj_Blaster_Grouper_33185_MAP_5	LINE	SjR2	1031	630	410	191	384
Sj_Blaster_Grouper_34745_MAP_4	LINE	Novel	1833	468	686	307	162
Sj_Blaster_Grouper_27622_MAP_3	LINE	RTE-1_MD_1p	935	698	489	285	196
Sj_Blaster_Grouper_33499_MAP_5	LINE	RTE-1_MD_1p	1194	492	678	285	233
Sj_Blaster_Grouper_34468_MAP_5	LINE	Novel	1668	449	474	248	278
Sj_Blaster_Grouper_31869_MAP_6	LINE	RTE-1_MD_1p	1224	406	719	318	215
Sj_Blaster_Grouper_32600_MAP_3	LINE	SjR2	1230	268	829	154	394
Sj_Blaster_Grouper_34161_MAP_8	LINE	RTE-1_MD_1p	1390	635	461	209	351
Sj_Blaster_Grouper_16471_MAP_3	LINE	RTE-1_MD_1p	1077	491	707	330	250
Sj_Blaster_Grouper_28389_MAP_20	LINE	SjR2	1018	652	613	263	228
Sj_Blaster_Grouper_33187_MAP_3	LINE	SjR2	1079	647	445	217	349
Sj_Blaster_Grouper_33813_MAP_3	LINE	Novel	1070	560	501	318	271
Sj_Blaster_Grouper_31703_MAP_3	LINE	RTE-1_MD_1p	1159	675	564	234	348
Sj_Blaster_Grouper_29964_MAP_5	LINE	Novel	1529	601	427	378	260
Sj_Blaster_Grouper_31089_MAP_5	LINE	RTE-1_MD_1p	1365	638	645	311	214
Sj_Blaster_Grouper_33997_MAP_5	LINE	Novel	1562	400	673	252	303
Sj_Blaster_Grouper_32216_MAP_3	LINE	RTE-1_MD_1p	1179	371	825	341	204
Sj_Blaster_Grouper_30687_MAP_3	LINE	Novel	1805	506	545	188	425
Sj_Blaster_Piler_669.0_MAP_6	LINE	Novel	1178	521	511	290	412
Sj_Blaster_Grouper_33789_MAP_3	LINE	Novel	1425	539	575	317	253
Sj_Blaster_Grouper_32672_MAP_3	LINE	Novel	1365	668	616	191	372
Sj_Blaster_Grouper_33782_MAP_3	LINE	RTE-1_MD_1p	1505	482	637	386	271
Sj_Blaster_Grouper_33171_MAP_3	LINE	Novel	1358	740	527	258	262
Sj_Blaster_Grouper_32543_MAP_3	LINE	Novel	1435	567	528	317	319
Sj_Blaster_Grouper_33110_MAP_3	LINE	RTE-1_MD_1p	1266	804	451	218	355
Sj_Blaster_Grouper_34120_MAP_3	LINE	Novel	1665	642	669	326	261

Sj_Blaster_Grouper_15782_MAP_3	LINE	RTE-1_MD_1p	1013	361	753	346	213
Sj_Blaster_Grouper_28439_MAP_6	LINE	Novel	2245	608	500	307	301
Sj_Blaster_Grouper_29432_MAP_4	LINE	Novel	1249	659	553	251	314
Sj_Blaster_Grouper_31033_MAP_4	LINE	RTE-1_MD_1p	1337	557	712	285	331
Sj_Blaster_Grouper_32623_MAP_6	LINE	RTE-1_MD_1p	1205	733	594	228	334
Sj_Blaster_Grouper_12277_MAP_4	LINE	RTE-1_MD_1p	765	601	819	217	207
Sj_Blaster_Grouper_33814_MAP_3	LINE	RTE-1_MD_1p	1363	503	679	358	280
Sj_Blaster_Grouper_34320_MAP_3	LINE	Novel	1506	559	645	296	364
Sj_Blaster_Grouper_32691_MAP_6	LINE	RTE-1_MD_1p	1627	812	459	318	242
Sj_Blaster_Grouper_31090_MAP_3	LINE	RTE-1_MD_1p	1598	761	497	280	310
Sj_Blaster_Grouper_32826_MAP_3	LINE	SjR2	1073	492	764	480	251
Sj_Blaster_Grouper_15820_MAP_20	LINE	SjR2	1103	549	665	421	261
Sj_Blaster_Grouper_31378_MAP_3	LINE	RTE-1_MD_1p	1381	601	754	336	244
Sj_Blaster_Grouper_15071_MAP_3	LINE	Novel	951	729	617	349	263
Sj_Blaster_Grouper_33921_MAP_3	LINE	SjR2	1263	156	1260	103	511
Sj_Blaster_Grouper_17411_MAP_3	LINE	Novel	1178	560	545	415	245
Sj_Blaster_Grouper_33378_MAP_3	LINE	PERERE-4_2p	2009	521	659	506	293
Sj_Blaster_Grouper_30300_MAP_3	LINE	RTE-1_MD_1p	2190	690	472	358	252
Sj_Blaster_Grouper_33889_MAP_3	LINE	RTE-1_MD_1p	1373	788	665	270	332
Sj_Blaster_Grouper_33553_MAP_3	LINE	RTE-1_MD_1p	1735	624	587	333	326
Sj_Blaster_Grouper_30233_MAP_3	LINE	Novel	1455	537	755	347	278
Sj_Blaster_Grouper_30763_MAP_3	LINE	SjR2	1439	711	704	291	395
Sj_Blaster_Grouper_32462_MAP_3	LINE	RTE-1_MD_1p	1867	621	765	391	288
Sj_Blaster_Grouper_32330_MAP_3	LINE	Novel	1334	779	565	208	425
Sj_Blaster_Grouper_31129_MAP_3	LINE	RTE-1_MD_1p	1463	755	682	221	318
Sj_Blaster_Grouper_30831_MAP_3	LINE	Novel	1194	656	586	411	291
Sj_Blaster_Grouper_33688_MAP_3	LINE	Novel	1816	668	719	411	212
Sj_Blaster_Grouper_34404_MAP_4	LINE	Novel	1441	644	570	375	399
Sj_Blaster_Grouper_32448_MAP_3	LINE	Novel	1790	645	567	349	346
Sj_Blaster_Grouper_32704_MAP_3	LINE	Novel	1829	676	405	449	321
Sj_Blaster_Grouper_30320_MAP_4	LINE	Novel	1369	456	718	279	427
Sj_Blaster_Grouper_32756_MAP_4	LINE	SjR2	1262	777	670	246	355
Sj_Blaster_Grouper_26581_MAP_3	LINE	SjR2	1413	695	613	328	400
Sj_Blaster_Grouper_33007_MAP_3	LINE	RTE-1_MD_1p	1282	692	773	451	273

Sj_Blaster_Grouper_27560_MAP_12	LINE	SjR2	1162	447	877	407	260
Sj_Blaster_Grouper_33925_MAP_3	LINE	Novel	1524	487	719	334	381
Sj_Blaster_Grouper_32278_MAP_3	LINE	RTE-1_MD_1p	1373	884	460	266	413
Sj_Blaster_Grouper_32135_MAP_3	LINE	Novel	1475	544	879	316	360
Sj_Blaster_Grouper_30525_MAP_3	LINE	Novel	1831	712	459	500	254
Sj_Blaster_Grouper_32675_MAP_3	LINE	Novel	1661	898	564	381	293
Sj_Blaster_Grouper_17633_MAP_4	LINE	Novel	1202	589	548	474	367
Sj_Blaster_Grouper_19227_MAP_3	LINE	Novel	1451	468	693	356	387
Sj_Blaster_Grouper_32604_MAP_3	LINE	Novel	1556	675	567	435	328
Sj_Blaster_Grouper_29879_MAP_3	LINE	Novel	1307	544	881	313	378
Sj_Blaster_Grouper_29866_MAP_3	LINE	Novel	1511	755	539	453	328
Sj_Blaster_Grouper_33002_MAP_3	LINE	Novel	1497	728	833	367	333
Sj_Blaster_Grouper_33630_MAP_8	LINE	RTE-1_MD_1p	1525	923	541	307	409
Sj_Blaster_Grouper_29433_MAP_3	LINE	Novel	1417	581	706	308	411
Sj_Blaster_Grouper_31985_MAP_3	LINE	Novel	1155	798	701	247	464
Sj_Blaster_Grouper_33389_MAP_3	LINE	RTE-1_MD_1p	1513	835	524	335	429
Sj_Blaster_Grouper_23399_MAP_20	LINE	Novel	1543	477	695	370	487
Sj_Blaster_Grouper_33942_MAP_3	LINE	Novel	1516	504	844	319	420
Sj_Blaster_Grouper_32182_MAP_5	LINE	RTE-1_MD_1p	1265	331	1297	90	547
Sj_Blaster_Grouper_34111_MAP_3	LINE	Novel	1796	870	765	357	351
Sj_Blaster_Grouper_31944_MAP_3	LINE	Novel	1768	798	630	356	364
Sj_Blaster_Grouper_33319_MAP_3	LINE	RTE-1_MD_1p	993	942	573	274	314
Sj_Blaster_Grouper_34254_MAP_5	LINE	Novel	1458	716	633	390	308
Sj_Blaster_Grouper_31816_MAP_3	LINE	Novel	2423	595	614	327	473
Sj_Blaster_Grouper_33972_MAP_3	LINE	Novel	2022	661	687	480	282
Sj_Blaster_Grouper_19676_MAP_3	LINE	Novel	1474	579	823	333	379
Sj_Blaster_Grouper_19251_MAP_3	LINE	Novel	1415	702	1059	388	249
Sj_Blaster_Grouper_30692_MAP_6	LINE	RTE-1_MD_1p	1339	311	1214	159	582
Sj_Blaster_Grouper_32387_MAP_3	LINE	Novel	1689	766	646	354	453
Sj_Blaster_Grouper_34362_MAP_3	LINE	SjR2	1534	106	1422	97	626
Sj_Blaster_Grouper_31641_MAP_3	LINE	Novel	1604	797	647	253	450
Sj_Blaster_Grouper_34594_MAP_4	LINE	Novel	2136	732	779	325	504
Sj_Blaster_Grouper_32247_MAP_3	LINE	RTE-1_MD_1p	1523	891	554	298	388
Sj_Blaster_Grouper_31677_MAP_3	LINE	Novel	1123	1113	656	330	369

Sj_Blaster_Grouper_34222_MAP_3	LINE	Novel	1289	777	720	325	487
Sj_Blaster_Grouper_30519_MAP_3	LINE	Novel	1454	773	945	270	484
Sj_Blaster_Grouper_26109_MAP_6	LINE	RTE-1_MD_1p	1914	707	739	268	490
Sj_Blaster_Grouper_34817_MAP_4	LINE	Novel	1698	818	695	423	414
Sj_Blaster_Grouper_33092_MAP_4	LINE	Novel	1485	712	884	424	315
Sj_Blaster_Grouper_34338_MAP_3	LINE	Novel	2228	723	917	478	355
Sj_Blaster_Grouper_32494_MAP_3	LINE	Novel	1897	528	760	372	506
Sj_Blaster_Grouper_30909_MAP_11	LINE	Novel	2140	903	732	437	345
Sj_Blaster_Grouper_33071_MAP_3	LINE	Novel	2419	710	744	301	502
Sj_Blaster_Grouper_32092_MAP_3	LINE	Novel	2353	620	720	458	395
Sj_Blaster_Grouper_33873_MAP_4	LINE	Novel	1722	242	1345	168	614
Sj_Blaster_Grouper_32445_MAP_5	LINE	RTE-1_MD_1p	1872	946	715	366	401
Sj_Blaster_Grouper_34964_MAP_3	LINE	Novel	1978	656	658	561	358
Sj_Blaster_Grouper_31133_MAP_8	LINE	Novel	2082	904	606	449	387
Sj_Blaster_Grouper_33654_MAP_3	LINE	Novel	1673	648	871	402	476
Sj_Blaster_Grouper_30585_MAP_7	LINE	Novel	2142	839	749	373	547
Sj_Blaster_Grouper_34671_MAP_6	LINE	RTE-1_MD_1p	1858	849	779	451	344
Sj_Blaster_Grouper_33495_MAP_3	LINE	RTE-1_MD_1p	1299	716	1069	419	272
Sj_Blaster_Grouper_33545_MAP_4	LINE	RTE-1_MD_1p	1945	720	969	407	365
Sj_Blaster_Grouper_34607_MAP_4	LINE	Novel	2039	778	904	564	370
Sj_Blaster_Grouper_32596_MAP_3	LINE	Novel	1385	800	810	357	547
Sj_Blaster_Grouper_29306_MAP_3	LINE	Novel	1339	656	744	356	616
Sj_Blaster_Grouper_34056_MAP_3	LINE	Novel	2096	628	953	382	425
Sj_Blaster_Grouper_32968_MAP_3	LINE	Novel	1987	754	755	363	504
Sj_Blaster_Grouper_33341_MAP_3	LINE	RTE-1_MD_1p	1715	903	762	376	400
Sj_Blaster_Grouper_34582_MAP_3	LINE	RTE-1_MD_1p	1667	865	810	503	338
Sj_Blaster_Grouper_34751_MAP_3	LINE	Novel	1376	691	967	493	345
Sj_Blaster_Grouper_31003_MAP_3	LINE	Novel	1580	702	991	405	462
Sj_Blaster_Grouper_25450_MAP_20	LINE	Novel	1868	605	1061	394	422
Sj_Blaster_Grouper_32394_MAP_4	LINE	Novel	1766	805	692	421	509
Sj_Blaster_Grouper_32520_MAP_3	LINE	Novel	1833	863	707	282	558
Sj_Blaster_Grouper_32566_MAP_16	LINE	Novel	1800	783	778	335	550
Sj_Blaster_Grouper_30552_MAP_5	LINE	Novel	1576	831	916	481	456
Sj_Blaster_Grouper_32760_MAP_3	LINE	Novel	1813	992	696	427	519

Sj_Blaster_Grouper_33662_MAP_3	LINE	Novel	3028	794	753	512	414
Sj_Blaster_Grouper_32910_MAP_5	LINE	RTE-1_MD_1p	1540	983	988	517	360
Sj_Blaster_Grouper_31983_MAP_5	LINE	RTE-1_MD_1p	1317	927	1007	408	323
Sj_Blaster_Grouper_33910_MAP_3	LINE	Novel	1958	850	1069	352	441
Sj_Blaster_Grouper_34496_MAP_3	LINE	Novel	1811	1009	703	310	515
Sj_Blaster_Grouper_34730_MAP_4	LINE	Novel	1707	844	894	430	547
Sj_Blaster_Grouper_34391_MAP_3	LINE	Novel	1389	775	1216	426	382
Sj_Blaster_Grouper_34614_MAP_3	LINE	SjR2	1567	1004	890	306	542
Sj_Blaster_Grouper_35077_MAP_3	LINE	Novel	2937	854	894	514	443
Sj_Blaster_Grouper_31648_MAP_5	LINE	RTE-1_MD_1p	1550	510	1535	276	613
Sj_Blaster_Grouper_31385_MAP_3	LINE	Novel	1913	753	937	475	442
Sj_Blaster_Grouper_25896_MAP_7	LINE	Novel	1900	973	731	500	417
Sj_Blaster_Grouper_33418_MAP_4	LINE	Novel	1752	980	855	461	488
Sj_Blaster_Grouper_34530_MAP_7	LINE	Novel	1498	730	746	639	479
Sj_Blaster_Grouper_33864_MAP_3	LINE	Novel	1542	755	704	618	443
Sj_Blaster_Grouper_30688_MAP_3	LINE	PERERE-4_2p	5542	896	745	626	628
Sj_Blaster_Grouper_26987_MAP_6	LINE	RTE-1_MD_1p	2389	773	1093	435	517
Sj_Blaster_Grouper_21914_MAP_6	LINE	Novel	1736	921	1078	492	344
Sj_Blaster_Grouper_31127_MAP_3	LINE	RTE-1_MD_1p	2149	936	1023	372	495
Sj_Blaster_Grouper_31369_MAP_3	LINE	Novel	1849	840	1087	468	560
Sj_Blaster_Grouper_32957_MAP_3	LINE	Novel	2491	723	909	417	552
Sj_Blaster_Grouper_22528_MAP_5	LINE	SjR2	1636	956	1073	519	406
Sj_Blaster_Grouper_32813_MAP_11	LINE	Novel	2089	963	961	396	613
Sj_Blaster_Grouper_34498_MAP_3	LINE	Novel	1961	678	1401	522	306
Sj_Blaster_Grouper_34105_MAP_4	LINE	Novel	1438	861	716	583	508
Sj_Blaster_Grouper_33373_MAP_5	LINE	Novel	1916	1040	772	539	423
Sj_Blaster_Grouper_30203_MAP_4	LINE	Novel	1770	919	1068	488	552
Sj_Blaster_Grouper_28965_MAP_5	LINE	Novel	1297	956	848	653	505
Sj_Blaster_Grouper_34116_MAP_4	LINE	Novel	2472	998	970	599	387
Sj_Blaster_Grouper_32741_MAP_3	LINE	Novel	2016	1289	844	470	397
Sj_Blaster_Grouper_33461_MAP_5	LINE	Novel	1776	951	746	379	674
Sj_Blaster_Grouper_30791_MAP_13	LINE	RTE-1_MD_1p	2619	687	876	370	652
Sj_Blaster_Grouper_34562_MAP_4	LINE	RTE-1_MD_1p	1528	785	1278	456	342
Sj_Blaster_Grouper_34217_MAP_5	LINE	SjR2	1625	1087	906	415	624

Sj_Blaster_Grouper_31521_MAP_15	LINE	Novel	2706	1021	813	537	427
Sj_Blaster_Grouper_19959_MAP_20	LINE	RTE-1_MD_1p	2142	882	742	678	395
Sj_Blaster_Grouper_34108_MAP_4	LINE	Novel	1362	962	813	436	602
Sj_Blaster_Grouper_29804_MAP_5	LINE	Novel	2463	1169	733	507	433
Sj_Blaster_Grouper_22756_MAP_20	LINE	Novel	2032	931	758	683	390
Sj_Blaster_Grouper_35033_MAP_3	LINE	Novel	3195	850	850	577	589
Sj_Blaster_Grouper_30362_MAP_3	LINE	RTE-1_MD_1p	1928	992	1048	599	395
Sj_Blaster_Grouper_33670_MAP_3	LINE	Novel	2028	861	962	456	590
Sj_Blaster_Grouper_32959_MAP_3	LINE	Novel	1952	1037	1032	475	498
Sj_Blaster_Grouper_34499_MAP_3	LINE	Novel	1772	877	994	393	718
Sj_Blaster_Grouper_23150_MAP_3	LINE	Novel	2082	943	1041	477	679
Sj_Blaster_Grouper_32695_MAP_3	LINE	Novel	1981	1165	956	398	513
Sj_Blaster_Grouper_31465_MAP_6	LINE	RTE-1_MD_1p	1450	1366	844	364	529
Sj_Blaster_Grouper_17201_MAP_3	LINE	RTE-1_MD_1p	1152	1374	786	324	577
Sj_Blaster_Grouper_19247_MAP_3	LINE	Novel	1413	866	902	589	482
Sj_Blaster_Grouper_30467_MAP_4	LINE	Novel	2303	831	895	422	678
Sj_Blaster_Grouper_30543_MAP_6	LINE	RTE-1_MD_1p	2655	1279	966	517	422
Sj_Blaster_Grouper_33063_MAP_3	LINE	Novel	2426	1419	781	569	436
Sj_Blaster_Grouper_34406_MAP_3	LINE	RTE-1_MD_1p	2702	498	1473	320	679
Sj_Blaster_Grouper_33360_MAP_3	LINE	RTE-1_MD_1p	1630	853	1357	564	335
Sj_Blaster_Grouper_33349_MAP_3	LINE	Novel	1920	893	801	597	605
Sj_Blaster_Grouper_30765_MAP_3	LINE	Novel	2008	991	1162	461	522
Sj_Blaster_Grouper_33211_MAP_3	LINE	Novel	2365	927	1002	326	695
Sj_Blaster_Grouper_30280_MAP_3	LINE	Novel	1898	872	1014	558	649
Sj_Blaster_Grouper_33724_MAP_4	LINE	Novel	2123	1061	1269	539	459
Sj_Blaster_Grouper_33278_MAP_3	LINE	Novel	2493	956	1094	643	331
Sj_Blaster_Grouper_31689_MAP_6	LINE	Novel	2452	949	797	652	569
Sj_Blaster_Grouper_34673_MAP_3	LINE	Novel	2482	977	1120	441	576
Sj_Blaster_Grouper_34869_MAP_5	LINE	Novel	2387	935	1102	505	697
Sj_Blaster_Grouper_31938_MAP_12	LINE	Novel	2350	1128	980	479	607
Sj_Blaster_Grouper_34720_MAP_6	LINE	RTE-1_AGp	1681	1135	1164	443	629
Sj_Blaster_Grouper_29582_MAP_3	LINE	Novel	2311	752	991	386	792
Sj_Blaster_Grouper_34525_MAP_3	LINE	Novel	2355	1224	1007	323	712
Sj_Blaster_Grouper_27461_MAP_20	LINE	RTE-1_MD_1p	2127	1082	1089	537	532

Sj_Blaster_Grouper_27221_MAP_20	LINE	Novel	2607	820	1158	469	647
Sj_Blaster_Grouper_32853_MAP_6	LINE	SjR2	1997	1404	965	409	621
Sj_Blaster_Grouper_34793_MAP_3	LINE	RTE-1_MD_1p	1909	1058	1210	557	466
Sj_Blaster_Grouper_29698_MAP_3	LINE	RTE-1_AGp	2186	646	1617	415	797
Sj_Blaster_Grouper_34942_MAP_3	LINE	Novel	2858	966	1033	520	604
Sj_Blaster_Grouper_33700_MAP_3	LINE	Novel	2004	1279	1113	544	545
Sj_Blaster_Grouper_34003_MAP_3	LINE	Novel	2191	877	1069	503	808
Sj_Blaster_Grouper_34916_MAP_3	LINE	Novel	2097	1183	1207	487	578
Sj_Blaster_Grouper_31774_MAP_3	LINE	Novel	1559	1107	939	595	604
Sj_Blaster_Grouper_33255_MAP_3	LINE	Novel	1909	1304	1167	490	713
Sj_Blaster_Grouper_34071_MAP_3	LINE	Novel	3292	333	1556	152	991
Sj_Blaster_Grouper_32292_MAP_3	LINE	RTE-1_MD_1p	1689	993	1430	607	375
Sj_Blaster_Grouper_34825_MAP_3	LINE	RTE-1_AGp	2119	1314	901	512	626
Sj_Blaster_Grouper_34348_MAP_3	LINE	RTE-1_MD_1p	1881	828	1403	542	441
Sj_Blaster_Grouper_33971_MAP_3	LINE	RTE-1_MD_1p	2345	1039	1358	487	566
Sj_Blaster_Grouper_33784_MAP_11	LINE	Novel	2497	1266	988	612	560
Sj_Blaster_Grouper_34028_MAP_3	LINE	Novel	2610	881	1092	463	796
Sj_Blaster_Grouper_32388_MAP_4	LINE	RTE-1_MD_1p	2203	985	1406	628	494
Sj_Blaster_Grouper_31859_MAP_3	LINE	Novel	1742	908	1454	528	479
Sj_Blaster_Grouper_32710_MAP_3	LINE	RTE-1_MD_1p	2070	1280	1095	429	601
Sj_Blaster_Grouper_34848_MAP_3	LINE	RTE-1_MD_1p	2292	1304	1043	541	611
Sj_Blaster_Grouper_30330_MAP_3	LINE	Novel	2031	1180	1167	550	714
Sj_Blaster_Recon_1_MAP_20	LINE	Novel	2412	1314	1088	438	547
Sj_Blaster_Grouper_33833_MAP_3	LINE	Novel	2360	1343	1060	602	607
Sj_Blaster_Grouper_29997_MAP_3	LINE	Novel	2402	1188	1116	685	626
Sj_Blaster_Grouper_35093_MAP_3	LINE	Novel	2689	1117	1145	614	612
Sj_Blaster_Grouper_34572_MAP_4	LINE	RTE-1_MD_1p	1640	982	1452	670	443
Sj_Blaster_Grouper_33999_MAP_7	LINE	Novel	2288	972	1336	531	541
Sj_Blaster_Grouper_84_MAP_20	LINE	Novel	2598	930	1185	544	746
Sj_Blaster_Grouper_32426_MAP_3	LINE	Novel	2086	1158	1372	651	473
Sj_Blaster_Grouper_33809_MAP_3	LINE	Novel	2914	1125	995	585	659
Sj_Blaster_Grouper_34072_MAP_3	LINE	Novel	1632	1388	969	478	499
Sj_Blaster_Grouper_34489_MAP_20	LINE	Novel	2027	992	1442	679	372
Sj_Blaster_Grouper_24442_MAP_8	LINE	Novel	1950	1049	1098	767	588

Sj_Blaster_Grouper_34951_MAP_3	LINE	Novel	2430	1008	1167	797	538
Sj_Blaster_Grouper_34883_MAP_3	LINE	Novel	1847	1503	968	521	532
Sj_Blaster_Grouper_34541_MAP_3	LINE	RTE-1_AGp	2608	785	1523	589	748
Sj_Blaster_Grouper_28692_MAP_3	LINE	Novel	3156	882	1161	557	713
Sj_Blaster_Grouper_34683_MAP_3	LINE	RTE-1_MD_1p	2072	1468	1130	528	594
Sj_Blaster_Grouper_34198_MAP_3	LINE	RTE-1_MD_1p	1627	1420	1209	426	589
Sj_Blaster_Grouper_34961_MAP_3	LINE	Novel	1556	1249	1328	623	452
Sj_Blaster_Grouper_33739_MAP_3	LINE	RTE-1_MD_1p	2523	1060	1286	639	626
Sj_Blaster_Grouper_34213_MAP_4	LINE	RTE-1_MD_1p	1803	1475	1247	418	588
Sj_Blaster_Grouper_33866_MAP_4	LINE	Novel	1635	1166	1241	626	497
Sj_Blaster_Grouper_34998_MAP_3	LINE	Novel	2263	1239	1302	520	622
Sj_Blaster_Grouper_34427_MAP_4	LINE	RTE-1_MD_1p	1914	941	1226	585	692
Sj_Blaster_Grouper_27031_MAP_4	LINE	Novel	1717	1012	1161	571	880
Sj_Blaster_Grouper_31623_MAP_4	LINE	Novel	2585	1257	1066	755	536
Sj_Blaster_Grouper_35055_MAP_3	LINE	RTE-1_MD_1p	1859	778	2081	283	772
Sj_Blaster_Grouper_33621_MAP_3	LINE	Novel	2255	788	1502	682	778
Sj_Blaster_Grouper_31401_MAP_4	LINE	Novel	2257	1069	1394	726	685
Sj_Blaster_Grouper_32073_MAP_3	LINE	RTE-1_MD_1p	1865	1263	1233	750	616
Sj_Blaster_Grouper_34159_MAP_3	LINE	RTE-1_MD_1p	2038	1163	1382	638	614
Sj_Blaster_Grouper_30384_MAP_15	LINE	Novel	3310	1316	1006	719	610
Sj_Blaster_Grouper_31492_MAP_3	LINE	Novel	3126	927	1250	635	770
Sj_Blaster_Grouper_32633_MAP_3	LINE	Novel	2573	1218	1270	773	640
Sj_Blaster_Grouper_34794_MAP_3	LINE	Novel	2741	1110	1338	642	630
Sj_Blaster_Grouper_31685_MAP_3	LINE	Novel	3076	1465	979	725	544
Sj_Blaster_Grouper_33362_MAP_5	LINE	Novel	1877	1161	1100	604	858
Sj_Blaster_Grouper_34215_MAP_3	LINE	Novel	1996	1254	1285	606	730
Sj_Blaster_Grouper_23188_MAP_3	LINE	Novel	2104	1052	1656	715	472
Sj_Blaster_Grouper_34228_MAP_3	LINE	RTE-1_MD_1p	1969	1583	1143	462	727
Sj_Blaster_Grouper_35027_MAP_3	LINE	RTE-1_MD_1p	2090	1166	1675	736	451
Sj_Blaster_Recon_33_MAP_20	LINE	Novel	3681	909	1434	588	780
Sj_Blaster_Grouper_28641_MAP_20	LINE	Novel	3481	1384	1017	782	593
Sj_Blaster_Grouper_32449_MAP_3	LINE	Novel	1745	1369	1832	652	448
Sj_Blaster_Grouper_32953_MAP_5	LINE	Novel	1740	1275	1622	752	463
Sj_Blaster_Grouper_32492_MAP_3	LINE	Novel	1905	1361	1311	710	706

Sj_Blaster_Grouper_33680_MAP_4	LINE	Novel	2700	1211	1101	536	917
Sj_Blaster_Grouper_34980_MAP_4	LINE	RTE-1_MD_1p	2033	1750	1189	482	688
Sj_Blaster_Grouper_34125_MAP_11	LINE	RTE-1_MD_1p	1484	995	1877	680	408
Sj_Blaster_Grouper_34123_MAP_6	LINE	Novel	1902	1173	1821	593	479
Sj_Blaster_Grouper_32060_MAP_3	LINE	Novel	3239	1543	1144	727	564
Sj_Blaster_Grouper_22744_MAP_20	LINE	RTE-1_AGp	1749	1468	1154	733	778
Sj_Blaster_Recon_5630_MAP_8	LINE	Novel	2345	1117	1491	780	604
Sj_Blaster_Grouper_35063_MAP_3	LINE	RTE-1_MD_1p	2240	1143	1270	688	873
Sj_Blaster_Grouper_34973_MAP_3	LINE	RTE-1_AGp	2059	1643	1096	670	686
Sj_Blaster_Grouper_33909_MAP_3	LINE	Novel	3032	1380	1365	578	863
Sj_Blaster_Grouper_32186_MAP_3	LINE	Novel	3558	1580	1208	757	549
Sj_Blaster_Grouper_34402_MAP_3	LINE	Novel	2008	1423	1167	803	775
Sj_Blaster_Grouper_35094_MAP_4	LINE	RTE-1_AGp	2210	900	1874	417	1135
Sj_Blaster_Grouper_34907_MAP_7	LINE	Novel	1924	1229	1438	885	655
Sj_Blaster_Grouper_29726_MAP_3	LINE	Novel	2637	865	1860	676	952
Sj_Blaster_Grouper_33955_MAP_3	LINE	Novel	3183	1713	1021	731	651
Sj_Blaster_Grouper_30486_MAP_3	LINE	Novel	2598	1219	1455	727	807
Sj_Blaster_Grouper_35022_MAP_3	LINE	Novel	2652	1244	1529	701	766
Sj_Blaster_Grouper_34832_MAP_5	LINE	Novel	2221	1301	1362	710	742
Sj_Blaster_Grouper_31522_MAP_12	LINE	RTE-1_MD_1p	3380	1387	1138	845	613
Sj_Blaster_Grouper_32655_MAP_3	LINE	Novel	2513	1574	1500	511	807
Sj_Blaster_Grouper_33901_MAP_5	LINE	Novel	3506	1244	1484	621	847
Sj_Blaster_Grouper_33182_MAP_3	LINE	RTE-1_MD_1p	1594	1611	1478	592	701
Sj_Blaster_Grouper_28801_MAP_3	LINE	Novel	3068	1564	1189	862	562
Sj_Blaster_Grouper_34012_MAP_3	LINE	Novel	2942	1102	1628	689	830
Sj_Blaster_Grouper_31463_MAP_3	LINE	Novel	2262	1256	1373	679	901
Sj_Blaster_Grouper_34908_MAP_3	LINE	Novel	1964	1202	1543	773	713
Sj_Blaster_Grouper_32933_MAP_3	LINE	Novel	2930	1041	1462	748	813
Sj_Blaster_Grouper_32628_MAP_5	LINE	RTE-1_MD_1p	2697	1384	1411	593	876
Sj_Blaster_Grouper_33915_MAP_4	LINE	Novel	3056	1776	1245	639	759
Sj_Blaster_Grouper_34216_MAP_3	LINE	Novel	3046	1492	1206	867	745
Sj_Blaster_Grouper_31719_MAP_3	LINE	Novel	3432	1186	1548	650	879
Sj_Blaster_Grouper_28140_MAP_7	LINE	Novel	3187	1262	1667	613	860
Sj_Blaster_Grouper_35114_MAP_3	LINE	Novel	4260	1313	1224	831	681

Sj_Blaster_Grouper_35025_MAP_3	LINE	Novel	2533	1776	1272	645	728
Sj_Blaster_Grouper_34557_MAP_4	LINE	Novel	2633	1313	1512	603	943
Sj_Blaster_Grouper_29506_MAP_10	LINE	Novel	2347	1665	1248	743	911
Sj_Blaster_Piler_516.0_MAP_6	LINE	RTE-1_MD_1p	3961	1109	1556	633	937
Sj_Blaster_Grouper_34731_MAP_3	LINE	Novel	2831	1378	1628	758	786
Sj_Blaster_Grouper_34914_MAP_4	LINE	Novel	2580	1225	1835	764	679
Sj_Blaster_Grouper_34379_MAP_3	LINE	Novel	2220	1757	1269	715	782
Sj_Blaster_Grouper_21530_MAP_3	LINE	Novel	1662	1295	1664	826	649
Sj_Blaster_Grouper_34576_MAP_3	LINE	Novel	2475	1424	1443	891	874
Sj_Blaster_Grouper_35035_MAP_3	LINE	Novel	2478	1373	1454	969	701
Sj_Blaster_Grouper_34248_MAP_3	LINE	Novel	2230	1404	1389	723	996
Sj_Blaster_Grouper_34434_MAP_3	LINE	Novel	3579	1344	1846	573	837
Sj_Blaster_Grouper_34815_MAP_5	LINE	Novel	2181	1321	1686	912	592
Sj_Blaster_Grouper_35002_MAP_3	LINE	Novel	3072	1593	1327	921	701
Sj_Blaster_Grouper_34327_MAP_3	LINE	Novel	2256	1464	1682	942	527
Sj_Blaster_Grouper_33897_MAP_3	LINE	Novel	1902	1451	1586	638	933
Sj_Blaster_Grouper_35031_MAP_3	LINE	Novel	2448	1805	1351	683	832
Sj_Blaster_Grouper_34903_MAP_3	LINE	RTE-1_MD_1p	2204	1950	1427	610	799
Sj_Blaster_Grouper_34725_MAP_3	LINE	Novel	2432	1585	1558	641	882
Sj_Blaster_Grouper_34963_MAP_4	LINE	Novel	2436	1325	1816	710	770
Sj_Blaster_Grouper_31325_MAP_20	LINE	Novel	2284	1370	1453	768	1040
Sj_Blaster_Grouper_34791_MAP_3	LINE	Novel	2506	1879	1360	798	781
Sj_Blaster_Grouper_33791_MAP_3	LINE	Novel	3169	1431	1700	618	1024
Sj_Blaster_Grouper_29645_MAP_3	LINE	Novel	4657	1689	1342	712	830
Sj_Blaster_Grouper_32213_MAP_4	LINE	Novel	4122	1808	1369	855	688
Sj_Blaster_Grouper_33867_MAP_3	LINE	RTE-1_MD_1p	1896	1480	1810	822	612
Sj_Blaster_Grouper_32929_MAP_6	LINE	Novel	2860	1369	1623	726	934
Sj_Blaster_Grouper_30482_MAP_3	LINE	Novel	3188	1337	1641	770	952
Sj_Blaster_Grouper_34930_MAP_3	LINE	Novel	2350	1313	2049	796	560
Sj_Blaster_Grouper_33974_MAP_5	LINE	Novel	2753	1608	1492	839	895
Sj_Blaster_Grouper_34999_MAP_5	LINE	Novel	2886	1510	1284	1117	741
Sj_Blaster_Grouper_35006_MAP_3	LINE	Novel	2054	1494	1775	884	627
Sj_Blaster_Grouper_34038_MAP_3	LINE	Novel	2535	1497	1359	845	987
Sj_Blaster_Grouper_33308_MAP_3	LINE	Novel	3229	1728	1448	946	822

Sj_Blaster_Grouper_34618_MAP_4	LINE	Novel	2596	554	2651	346	1457
Sj_Blaster_Grouper_34661_MAP_3	LINE	RTE-1_MD_1p	2008	1890	1530	629	937
Sj_Blaster_Grouper_34831_MAP_3	LINE	Novel	2752	1337	1235	1167	821
Sj_Blaster_Grouper_31634_MAP_3	LINE	Novel	3284	1661	1242	1074	747
Sj_Blaster_Grouper_33451_MAP_4	LINE	Novel	3543	1399	1485	744	1096
Sj_Blaster_Grouper_35117_MAP_3	LINE	Novel	2870	2027	1498	745	753
Sj_Blaster_Grouper_33571_MAP_5	LINE	Novel	2165	1854	1551	541	1022
Sj_Blaster_Grouper_35007_MAP_7	LINE	RTE-1_MD_1p	3491	1649	1301	1164	707
Sj_Blaster_Grouper_28371_MAP_20	LINE	Novel	3504	1643	1344	1032	834
Sj_Blaster_Grouper_34668_MAP_4	LINE	Novel	2143	1410	2038	855	771
Sj_Blaster_Grouper_33765_MAP_4	LINE	Novel	2042	1538	2002	980	770
Sj_Blaster_Grouper_32440_MAP_3	LINE	Novel	3739	1462	1324	1235	759
Sj_Blaster_Grouper_30707_MAP_3	LINE	Novel	2479	1685	1645	802	908
Sj_Blaster_Grouper_34659_MAP_3	LINE	Novel	3086	1553	1793	879	1068
Sj_Blaster_Grouper_34020_MAP_3	LINE	Novel	2430	1520	1758	910	845
Sj_Blaster_Grouper_34529_MAP_3	LINE	Novel	2441	1980	1710	615	1034
Sj_Blaster_Grouper_27329_MAP_20	LINE	Novel	3977	2008	1501	986	733
Sj_Blaster_Grouper_34894_MAP_3	LINE	Novel	3099	1432	1469	1234	801
Sj_Blaster_Grouper_32410_MAP_3	LINE	Novel	2967	1758	1763	1065	800
Sj_Blaster_Grouper_31065_MAP_3	LINE	Novel	3611	1452	1742	870	1071
Sj_Blaster_Grouper_34545_MAP_5	LINE	Novel	2681	1664	1534	889	1203
Sj_Blaster_Grouper_31939_MAP_4	LINE	Novel	2826	1882	1772	966	896
Sj_Blaster_Grouper_33425_MAP_15	LINE	Novel	2097	1616	2046	1036	705
Sj_Blaster_Grouper_34989_MAP_3	LINE	Novel	2403	2181	1632	656	978
Sj_Blaster_Grouper_34896_MAP_5	LINE	Novel	2454	1695	1678	961	1127
Sj_Blaster_Grouper_31548_MAP_3	LINE	Novel	3436	1812	1577	1112	834
Sj_Blaster_Grouper_33843_MAP_5	LINE	Novel	3219	1382	1888	751	948
Sj_Blaster_Grouper_21415_MAP_15	LINE	RTE-1_MD_1p	1882	1936	1513	934	938
Sj_Blaster_Grouper_28765_MAP_5	LINE	Novel	2155	1993	1693	951	1001
Sj_Blaster_Grouper_33014_MAP_3	LINE	Novel	3296	1664	1478	945	1050
Sj_Blaster_Grouper_33872_MAP_3	LINE	RTE-1_MD_1p	2615	1625	2154	485	1242
Sj_Blaster_Grouper_34738_MAP_5	LINE	Novel	2957	1967	1544	782	1085
Sj_Blaster_Grouper_34867_MAP_3	LINE	Novel	2872	1927	1719	712	951
Sj_Blaster_Grouper_32804_MAP_6	LINE	Novel	2472	1815	2092	965	736

Sj_Blaster_Grouper_34849_MAP_4	LINE	Novel	2864	1682	2054	1081	696
Sj_Blaster_Grouper_28722_MAP_3	LINE	Novel	2161	992	2424	628	1369
Sj_Blaster_Grouper_31697_MAP_3	LINE	Novel	2645	1677	1802	1186	860
Sj_Blaster_Grouper_30779_MAP_7	LINE	Novel	2257	1677	1987	1093	809
Sj_Blaster_Grouper_33948_MAP_4	LINE	Novel	2900	1861	1991	875	1133
Sj_Blaster_Grouper_32166_MAP_3	LINE	Novel	2018	1540	2162	1126	837
Sj_Blaster_Grouper_34542_MAP_4	LINE	Novel	2527	2114	1719	948	968
Sj_Blaster_Grouper_35064_MAP_3	LINE	Novel	4669	1411	2434	673	1315
Sj_Blaster_Grouper_33528_MAP_9	LINE	Novel	1862	439	3509	390	1519
Sj_Blaster_Grouper_35016_MAP_3	LINE	Novel	3976	1049	2388	795	1325
Sj_Blaster_Grouper_34698_MAP_5	LINE	Novel	2373	1519	2029	946	1014
Sj_Blaster_Grouper_29584_MAP_3	LINE	Novel	2517	1634	1692	1023	1251
Sj_Blaster_Grouper_33478_MAP_3	LINE	Novel	2294	1638	2042	1167	866
Sj_Blaster_Grouper_33919_MAP_3	LINE	Novel	2565	1936	2166	1003	863
Sj_Blaster_Grouper_33286_MAP_3	LINE	Novel	4049	1769	1660	1197	895
Sj_Blaster_Grouper_33835_MAP_4	LINE	Novel	4127	1957	1644	1141	949
Sj_Blaster_Grouper_34787_MAP_5	LINE	Novel	3148	2048	1607	984	999
Sj_Blaster_Grouper_26538_MAP_3	LINE	Novel	2932	1953	1757	1007	1021
Sj_Blaster_Grouper_28056_MAP_3	LINE	Novel	2479	1191	2124	778	1445
Sj_Blaster_Grouper_34295_MAP_3	LINE	Novel	2496	1741	1829	966	1084
Sj_Blaster_Grouper_34168_MAP_4	LINE	Novel	2473	1771	2052	1061	853
Sj_Blaster_Grouper_35067_MAP_3	LINE	Novel	4109	2341	1763	1063	912
Sj_Blaster_Grouper_34765_MAP_7	LINE	RTE-1_MD_1p	3026	2029	1894	846	1217
Sj_Blaster_Grouper_34692_MAP_3	LINE	Novel	3267	1871	1811	970	1082
Sj_Blaster_Grouper_30814_MAP_6	LINE	Novel	2334	1737	2121	1059	872
Sj_Blaster_Grouper_34767_MAP_6	LINE	Novel	2196	1698	2180	949	1010
Sj_Blaster_Grouper_35079_MAP_3	LINE	Novel	3607	1236	2495	924	1275
Sj_Blaster_Grouper_32539_MAP_3	LINE	Novel	2364	1768	2067	1135	916
Sj_Blaster_Grouper_30193_MAP_4	LINE	Novel	2279	1750	2165	951	964
Sj_Blaster_Grouper_31011_MAP_5	LINE	Novel	2874	1972	1745	1128	879
Sj_Blaster_Grouper_34371_MAP_8	LINE	RTE-1_MD_1p	2403	1975	2260	1014	850
Sj_Blaster_Grouper_34911_MAP_3	LINE	Novel	3221	2097	2066	1231	920
Sj_Blaster_Grouper_34653_MAP_5	LINE	Novel	2909	1618	2175	726	1323
Sj_Blaster_Grouper_35086_MAP_5	LINE	RTE-1_MD_1p	4725	2040	1525	1305	889

Sj_Blaster_Grouper_33573_MAP_5	LINE	Novel	4278	1722	1940	934	1269
Sj_Blaster_Grouper_35095_MAP_3	LINE	Novel	3385	2016	1804	1095	1081
Sj_Blaster_Grouper_28608_MAP_3	LINE	Novel	3308	2212	1990	1168	1063
Sj_Blaster_Grouper_26592_MAP_3	LINE	RTE-1_MD_1p	2559	2016	2592	1020	834
Sj_Blaster_Grouper_33034_MAP_3	LINE	Novel	4061	2366	1712	1018	967
Sj_Blaster_Grouper_34474_MAP_3	LINE	RTE-1_MD_1p	2830	942	3482	452	1287
Sj_Blaster_Grouper_33979_MAP_3	LINE	Novel	2906	2225	2093	839	1265
Sj_Blaster_Grouper_34207_MAP_3	LINE	Novel	3102	1756	2338	1085	1035
Sj_Blaster_Grouper_32626_MAP_4	LINE	Novel	2947	2263	1981	879	1266
Sj_Blaster_Grouper_33669_MAP_3	LINE	Novel	3066	2118	1889	811	1339
Sj_Blaster_Grouper_34384_MAP_3	LINE	Novel	3210	1891	2307	1117	1064
Sj_Blaster_Grouper_34158_MAP_3	LINE	Novel	2937	2088	2270	1240	873
Sj_Blaster_Grouper_34913_MAP_3	LINE	Novel	3182	1938	2297	1064	1107
Sj_Blaster_Grouper_31933_MAP_3	LINE	Novel	2836	1907	2430	1100	1029
Sj_Blaster_Grouper_34195_MAP_4	LINE	Novel	2495	2429	1919	903	1240
Sj_Blaster_Grouper_34870_MAP_3	LINE	Novel	2867	2454	1860	1066	1063
Sj_Blaster_Grouper_33668_MAP_3	LINE	Novel	3821	2346	1930	978	1187
Sj_Blaster_Grouper_32879_MAP_3	LINE	Novel	3595	1731	2060	1093	1256
Sj_Blaster_Grouper_33538_MAP_3	LINE	Novel	3126	2597	1968	995	1121
Sj_Blaster_Grouper_34658_MAP_3	LINE	Novel	3962	2390	1984	1126	1174
Sj_Blaster_Grouper_28703_MAP_3	LINE	Novel	3458	2272	2010	1228	1106
Sj_Blaster_Grouper_25627_MAP_7	LINE	Novel	2638	1872	2006	1519	1171
Sj_Blaster_Grouper_34640_MAP_5	LINE	Novel	2446	2027	2325	1167	1103
Sj_Blaster_Grouper_28823_MAP_3	LINE	Novel	3077	1751	2433	912	1533
Sj_Blaster_Grouper_35071_MAP_3	LINE	RTE-1_MD_1p	3349	1979	2358	1175	1116
Sj_Blaster_Grouper_34681_MAP_4	LINE	Novel	2241	669	4032	322	1696
Sj_Blaster_Grouper_34351_MAP_3	LINE	Novel	2294	1602	3070	682	1523
Sj_Blaster_Grouper_35026_MAP_3	LINE	Novel	3405	2378	1928	1220	1182
Sj_Blaster_Grouper_35018_MAP_5	LINE	Novel	3088	2021	2287	1322	1021
Sj_Blaster_Grouper_34337_MAP_3	LINE	Novel	2991	2138	2468	1016	1156
Sj_Blaster_Grouper_32155_MAP_3	LINE	RTE-1_MD_1p	3505	1678	2759	860	1461
Sj_Blaster_Grouper_35096_MAP_3	LINE	Novel	3711	1889	2491	1271	1070
Sj_Blaster_Grouper_34680_MAP_3	LINE	RTE-1_MD_1p	4141	2181	2228	1112	1294
Sj_Blaster_Grouper_34965_MAP_4	LINE	Novel	4643	932	3516	560	1968

Sj_Blaster_Grouper_34761_MAP_4	LINE	Novel	2829	2405	2055	980	1495
Sj_Blaster_Grouper_33289_MAP_12	LINE	RTE-1_MD_1p	3161	2098	2408	1464	1016
Sj_Blaster_Grouper_33264_MAP_3	LINE	Novel	2430	1015	3660	609	1584
Sj_Blaster_Grouper_34744_MAP_3	LINE	Novel	3295	2205	1953	1417	1216
Sj_Blaster_Grouper_33704_MAP_3	LINE	Novel	4146	2225	2413	1230	1268
Sj_Blaster_Grouper_32878_MAP_3	LINE	Novel	4731	2070	2382	1086	1507
Sj_Blaster_Grouper_35153_MAP_3	LINE	Novel	4202	2409	2525	1195	1265
Sj_Blaster_Grouper_34289_MAP_7	LINE	Novel	2511	542	4433	381	1926
Sj_Blaster_Grouper_30294_MAP_20	LINE	Novel	2818	2202	2503	1241	1266
Sj_Blaster_Grouper_33770_MAP_3	LINE	Novel	3506	2316	2173	1019	1527
Sj_Blaster_Grouper_32847_MAP_7	LINE	Novel	3431	2140	2614	1260	1183
Sj_Blaster_Grouper_33977_MAP_3	LINE	Novel	3687	2273	2847	1357	1123
Sj_Blaster_Grouper_34141_MAP_4	LINE	Novel	2973	1836	3124	944	1605
Sj_Blaster_Grouper_34927_MAP_5	LINE	Novel	3034	2588	2200	1251	1437
Sj_Blaster_Grouper_34256_MAP_7	LINE	Novel	3669	2197	2567	1396	1148
Sj_Blaster_Grouper_31485_MAP_3	LINE	Novel	3631	2194	2542	1317	1297
Sj_Blaster_Grouper_33576_MAP_3	LINE	Novel	5683	2102	2270	1269	1536
Sj_Blaster_Grouper_35101_MAP_3	LINE	Novel	3741	2316	2392	1378	1343
Sj_Blaster_Grouper_30045_MAP_3	LINE	Novel	4116	2596	2455	1278	1363
Sj_Blaster_Grouper_34573_MAP_3	LINE	Novel	3890	2648	2139	1333	1257
Sj_Blaster_Grouper_31328_MAP_20	LINE	Novel	4866	2271	2619	1257	1407
Sj_Blaster_Grouper_34872_MAP_3	LINE	Novel	4578	2604	2202	1050	1511
Sj_Blaster_Grouper_34928_MAP_3	LINE	Novel	3344	2720	2299	1240	1451
Sj_Blaster_Grouper_33743_MAP_3	LINE	Novel	3276	2551	2470	1279	1371
Sj_Blaster_Grouper_31373_MAP_3	LINE	Novel	5777	2596	2568	1452	1297
Sj_Blaster_Grouper_31829_MAP_4	LINE	Novel	3343	2753	2346	1224	1425
Sj_Blaster_Grouper_30440_MAP_3	LINE	Novel	3128	2629	2336	1299	1560
Sj_Blaster_Grouper_35023_MAP_3	LINE	Novel	4428	2524	2477	1489	1111
Sj_Blaster_Grouper_34414_MAP_3	LINE	Novel	3009	1361	3936	883	1656
Sj_Blaster_Grouper_35048_MAP_3	LINE	Novel	3999	2730	2168	1466	1306
Sj_Blaster_Grouper_34897_MAP_3	LINE	Novel	5577	2369	2429	1485	1329
Sj_Blaster_Grouper_35037_MAP_3	LINE	Novel	4144	2971	2211	1401	1416
Sj_Blaster_Grouper_34433_MAP_3	LINE	Novel	5241	2729	2249	1565	1357
Sj_Blaster_Grouper_34812_MAP_3	LINE	Novel	4769	2744	2222	1427	1409

Sj_Blaster_Grouper_34024_MAP_4	LINE	Novel	3949	2750	2769	1320	1601
Sj_Blaster_Grouper_34388_MAP_3	LINE	Novel	3859	2512	2846	1463	1303
Sj_Blaster_Grouper_34809_MAP_3	LINE	Novel	4276	2603	2457	1452	1774
Sj_Blaster_Grouper_34824_MAP_3	LINE	Novel	4669	2895	2265	1420	1485
Sj_Blaster_Grouper_34016_MAP_3	LINE	Novel	3455	2478	3042	1537	1411
Sj_Blaster_Grouper_31814_MAP_3	LINE	Novel	3659	2932	2387	1445	1639
Sj_Blaster_Grouper_33968_MAP_3	LINE	Novel	2790	428	5234	249	2480
Sj_Blaster_Grouper_34413_MAP_3	LINE	Novel	4039	2398	2874	1437	1625
Sj_Blaster_Grouper_33405_MAP_3	LINE	Novel	3234	1748	3666	1045	1927
Sj_Blaster_Grouper_33129_MAP_6	LINE	Novel	3778	2545	2831	1506	1518
Sj_Blaster_Grouper_35057_MAP_3	LINE	Novel	4656	2809	2691	1212	1751
Sj_Blaster_Grouper_34136_MAP_3	LINE	Novel	3550	2472	2955	1594	1532
Sj_Blaster_Grouper_34882_MAP_3	LINE	Novel	5109	2825	2652	1245	1680
Sj_Blaster_Grouper_34702_MAP_9	LINE	Novel	3571	2828	2579	1677	1672
Sj_Blaster_Grouper_34476_MAP_3	LINE	Novel	4297	3126	2757	1634	1532
Sj_Blaster_Grouper_34647_MAP_5	LINE	Novel	3421	3147	2466	1426	1625
Sj_Blaster_Grouper_34648_MAP_3	LINE	Novel	3961	2689	3063	1624	1628
Sj_Blaster_Grouper_34839_MAP_3	LINE	Novel	4691	2849	2794	1486	1824
Sj_Blaster_Grouper_30422_MAP_17	LINE	Novel	4745	3188	2952	1477	1679
Sj_Blaster_Grouper_34616_MAP_5	LINE	Novel	3957	2821	3312	1625	1677
Sj_Blaster_Piler_105.0_MAP_20	LINE	Novel	4096	2825	3359	1615	1697
Sj_Blaster_Grouper_34393_MAP_4	LINE	Novel	3335	2829	3458	1803	1486
Sj_Blaster_Grouper_31737_MAP_3	LINE	Novel	4265	2952	3372	1664	1575
Sj_Blaster_Grouper_35045_MAP_3	LINE	Novel	5287	2932	3431	1790	1560
Sj_Blaster_Grouper_35097_MAP_3	LINE	Novel	4781	3114	3100	1771	1529
Sj_Blaster_Grouper_34932_MAP_7	LINE	Novel	3841	2876	3334	1789	1781
Sj_Blaster_Grouper_35112_MAP_4	LINE	RTE-1_MD_1p	4652	3640	2951	1849	1817
Sj_Blaster_Grouper_34785_MAP_3	LINE	Novel	4027	2062	4564	1343	2153
Sj_Blaster_Grouper_34712_MAP_3	LINE	Novel	4482	3063	3579	1781	1780
Sj_Blaster_Grouper_34775_MAP_3	LINE	Novel	4086	2125	4540	1475	2117
Sj_Blaster_Grouper_35109_MAP_4	LINE	Novel	4676	3585	3280	1866	1835
Sj_Blaster_Grouper_34536_MAP_3	LINE	Novel	4566	3171	3857	1822	1812
Sj_Blaster_Grouper_35062_MAP_3	LINE	Novel	4975	3641	3085	1797	1866
Sj_Blaster_Grouper_34469_MAP_4	LINE	Novel	4514	3547	3133	1926	1767

Sj_Blaster_Grouper_34410_MAP_5	LINE	Novel	4385	3143	3581	1870	1905
Sj_Blaster_Grouper_34922_MAP_3	LINE	RTE-1_MD_1p	4285	1287	6151	788	2676
Sj_Blaster_Grouper_34426_MAP_8	LINE	RTE-1_MD_1p	4164	3052	3623	1849	1982
Sj_Blaster_Grouper_35164_MAP_3	LINE	Novel	5359	2969	3724	1740	1918
Sj_Blaster_Grouper_33855_MAP_4	LINE	Novel	3863	3158	3910	2008	1553
Sj_Blaster_Grouper_34898_MAP_3	LINE	Novel	5433	3457	3641	2003	1830
Sj_Blaster_Grouper_34950_MAP_3	LINE	Novel	5229	3271	3684	1912	1960
Sj_Blaster_Grouper_33636_MAP_13	LINE	Novel	4992	3835	3232	2211	1983
Sj_Blaster_Grouper_35163_MAP_3	LINE	Novel	4773	4286	3468	1971	1994
Sj_Blaster_Grouper_34686_MAP_9	LINE	Novel	4491	3976	3587	2033	2103
Sj_Blaster_Grouper_34993_MAP_3	LINE	Novel	5206	3463	4335	2012	1981
Sj_Blaster_Grouper_34747_MAP_6	LINE	Novel	4544	4292	3642	2002	2228
Sj_Blaster_Grouper_34981_MAP_6	LINE	Novel	4994	3474	4038	2166	2232
Sj_Blaster_Grouper_34279_MAP_3	LINE	Novel	5676	3756	3715	2059	2523
Sj_Blaster_Grouper_34784_MAP_11	LINE	Novel	5087	4637	3834	2025	2138
Sj_Blaster_Grouper_34996_MAP_7	LINE	Novel	5812	4017	4503	2185	2422
Sj_Blaster_Grouper_34983_MAP_3	LINE	Novel	5749	3956	4275	2186	2503
Sj_Blaster_Grouper_34994_MAP_3	LINE	Novel	5347	4115	4623	2264	2285
Sj_Blaster_Grouper_34997_MAP_3	LINE	Novel	6241	4249	4520	2365	2742
Sj_Blaster_Grouper_34995_MAP_4	LINE	Novel	5826	4423	5243	2642	2477
