# Supplementary material for: Identification and characterization of microRNAs and endogenous siRNAs in Schistosoma japonicum
Source: BMC Genomics. 2010 Jan 21;11:55. doi: 10.1186/1471-2164-11-55 (PMC2820009; doi:10.1186/1471-2164-11-55)
Supplement: Additional file 3 — SiRNAs derived from LTR. This file contains the information of the identified transposon-LTR in the S. japonicum genome and the derived siRNAs. [file 1471-2164-11-55-S3.PDF]

siRNAs derived from LTR

Name	Type	Annotation	TE Length	siRNAs (Adult)		siRNAs (schistosomula)	
				# Sense siRNAs	# AntiSense siRNAs	# Sense siRNAs	# AntiSense siRNAs
Sj_Blaster_Recon_3013_MAP_4	LTR	SACI-5_1p	2910	0	0	2	0
Sj_Blaster_Recon_3486_MAP_3	LTR	SACI-7_2p	1989	0	0	1	0
Sj_Blaster_Grouper_20817_MAP_3	LTR	BEL11_AGp	1036	1	0	0	0
Sj_Blaster_Grouper_20449_MAP_3	LTR	GYPSY60_AG2p	1606	1	0	0	0
Sj_Blaster_Recon_797_MAP_4	LTR	SACI-7_2p	1899	0	0	0	1
Sj_Blaster_Recon_8745_MAP_6	LTR	NONAUT-3_1p	2425	0	1	0	0
Sj_Blaster_Recon_1724_MAP_4	LTR	BEL1-I_SM_1p	1289	0	0	0	1
Sj_Blaster_Recon_9183_MAP_5	LTR	Novel	1523	0	0	1	0
Sj_Blaster_Recon_9971_MAP_4	LTR	Gypsy9-I_AP_1p	1388	0	1	0	0
Sj_Blaster_Recon_5303_MAP_3	LTR	BN000804_1p	559	0	0	0	1
Sj_Blaster_Grouper_8151_MAP_3	LTR	Novel	595	1	0	0	0
Sj_Blaster_Grouper_12468_MAP_3	LTR	Novel	779	0	1	0	0
Sj_Blaster_Grouper_30877_MAP_3	LTR	Gypsy9-I_AP_1p	1302	0	0	0	1
Sj_Blaster_Grouper_10058_MAP_3	LTR	NONAUT-5_1p	662	0	0	0	1
Sj_Blaster_Grouper_33172_MAP_3	LTR	SACI-5_1p	6182	0	0	1	0
Sj_Blaster_Recon_13828_MAP_3	LTR	GYPSY53_AG2p	759	1	0	0	0
Sj_Blaster_Grouper_19393_MAP_4	LTR	Novel	1343	0	0	1	0
Sj_Blaster_Grouper_33044_MAP_3	LTR	Novel	7122	0	0	1	0
Sj_Blaster_Recon_17152_MAP_3	LTR	NONAUT-5_1p	460	0	1	0	0
Sj_Blaster_Grouper_18818_MAP_3	LTR	Novel	1350	0	0	0	1
Sj_Blaster_Grouper_17796_MAP_3	LTR	Novel	1221	0	0	0	1
Sj_Blaster_Recon_5191_MAP_3	LTR	SACI-7_2p	2691	0	0	1	0
Sj_Blaster_Recon_6058_MAP_5	LTR	SACI-7_2p	1820	0	0	1	0
Sj_Blaster_Recon_8863_MAP_3	LTR	GYPSY21_AGp	1419	1	0	1	0
Sj_Blaster_Grouper_18365_MAP_3	LTR	Novel	1300	0	2	0	0
Sj_Blaster_Recon_8544_MAP_3	LTR	SACI-7_2p	3446	0	1	0	1
Sj_Blaster_Grouper_16347_MAP_3	LTR	Novel	1063	0	2	0	0
Sj_Blaster_Grouper_3497_MAP_3	LTR	Novel	458	2	0	0	0
Sj_Blaster_Recon_5531_MAP_4	LTR	SACI-7_2p	2484	0	3	0	0

Sj_Blaster_Recon_12023_MAP_4	LTR	Novel	1268	0	2	0	0
Sj_Blaster_Grouper_11167_MAP_3	LTR	SACI-7_2p	709	0	0	0	0
Sj_Blaster_Grouper_19071_MAP_3	LTR	Novel	1365	0	0	2	0
Sj_Blaster_Grouper_8811_MAP_3	LTR	Novel	639	0	2	0	0
Sj_Blaster_Recon_10382_MAP_3	LTR	Novel	772	0	1	0	1
Sj_Blaster_Grouper_9718_MAP_4	LTR	Novel	659	0	2	0	0
Sj_Blaster_Grouper_15862_MAP_4	LTR	Novel	1046	2	0	0	0
Sj_Blaster_Grouper_9580_MAP_3	LTR	Novel	654	0	2	0	0
Sj_Blaster_Grouper_33937_MAP_3	LTR	BN000804_1p	2150	1	0	0	1
Sj_Blaster_Grouper_395_MAP_3	LTR	GYPSY1_AG2p	356	0	1	1	0
Sj_Blaster_Grouper_16205_MAP_3	LTR	Novel	1076	2	0	0	0
Sj_Blaster_Recon_14020_MAP_6	LTR	Novel	1411	0	2	0	0
Sj_Blaster_Grouper_19651_MAP_6	LTR	Novel	1713	4	0	0	0
Sj_Blaster_Grouper_19127_MAP_3	LTR	Novel	1388	0	0	2	0
Sj_Blaster_Grouper_19562_MAP_4	LTR	Novel	1462	2	0	0	0
Sj_Blaster_Grouper_18658_MAP_7	LTR	Novel	1332	0	0	0	2
Sj_Blaster_Recon_11695_MAP_3	LTR	Novel	1046	3	0	0	0
Sj_Blaster_Recon_18593_MAP_5	LTR	NONAUT-5_1p	983	0	0	1	2
Sj_Blaster_Recon_14906_MAP_4	LTR	Novel	1690	3	0	0	0
Sj_Blaster_Grouper_4518_MAP_3	LTR	GYPSY49_AG2p	487	0	3	1	0
Sj_Blaster_Grouper_13691_MAP_3	LTR	Novel	850	0	3	0	0
Sj_Blaster_Recon_3303_MAP_3	LTR	Novel	866	0	3	0	0
Sj_Blaster_Grouper_10868_MAP_4	LTR	Novel	701	0	3	0	0
Sj_Blaster_Grouper_18263_MAP_5	LTR	Novel	1451	0	0	3	0
Sj_Blaster_Recon_1360_MAP_3	LTR	Novel	909	0	3	0	0
Sj_Blaster_Recon_5735_MAP_3	LTR	Novel	1956	0	3	0	0
Sj_Blaster_Grouper_23012_MAP_3	LTR	Novel	1125	0	0	3	0
Sj_Blaster_Recon_1916_MAP_6	LTR	Novel	1895	0	0	0	3
Sj_Blaster_Grouper_8850_MAP_3	LTR	Novel	623	3	0	0	0
Sj_Blaster_Grouper_14722_MAP_3	LTR	Novel	923	0	3	0	0
Sj_Blaster_Piler_678.148_MAP_4	LTR	Novel	1059	0	0	3	0
Sj_Blaster_Grouper_16745_MAP_3	LTR	Novel	1104	0	3	0	0
Sj_Blaster_Recon_16799_MAP_4	LTR	BN000804_1p	1091	2	0	0	0
Sj_Blaster_Recon_1781_MAP_3	LTR	GYPSY48_AG2p	1562	3	0	0	1

Sj_Blaster_Grouper_24946_MAP_4	LTR	Novel	1438	0	0	0	3
Sj_Blaster_Recon_1536_MAP_5	LTR	Novel	3218	0	0	0	3
Sj_Blaster_Recon_9014_MAP_5	LTR	Novel	2118	0	3	0	0
Sj_Blaster_Grouper_13293_MAP_4	LTR	Novel	837	0	3	0	0
Sj_Blaster_Grouper_18209_MAP_3	LTR	Novel	1272	0	3	0	0
Sj_Blaster_Grouper_19279_MAP_3	LTR	Novel	1418	0	0	3	0
Sj_Blaster_Grouper_4678_MAP_3	LTR	SACI-7_2p	491	4	0	0	0
Sj_Blaster_Grouper_4755_MAP_3	LTR	Novel	493	1	0	9	0
Sj_Blaster_Grouper_12284_MAP_7	LTR	GYPSY70_AG2p	774	0	1	1	2
Sj_Blaster_Grouper_13596_MAP_4	LTR	Novel	845	0	4	0	0
Sj_Blaster_Grouper_27027_MAP_3	LTR	Novel	1673	0	0	0	4
Sj_Blaster_Recon_6308_MAP_7	LTR	BN000804_1p	4704	1	0	3	0
Sj_Blaster_Recon_7893_MAP_3	LTR	GYPSY1_AG2p	1250	2	1	0	1
Sj_Blaster_Grouper_31357_MAP_3	LTR	Novel	4784	0	0	0	4
Sj_Blaster_Recon_8007_MAP_7	LTR	Novel	1341	0	0	4	0
Sj_Blaster_Recon_4715_MAP_7	LTR	BN000804_1p	4610	2	1	0	1
Sj_Blaster_Recon_15270_MAP_3	LTR	GYPSY6-I_CB_ORF	1655	2	2	0	1
Sj_Blaster_Grouper_30748_MAP_3	LTR	Novel	5675	0	0	0	4
Sj_Blaster_Recon_5201_MAP_4	LTR	SACI-7_2p	1258	0	2	0	2
Sj_Blaster_Grouper_16155_MAP_4	LTR	Novel	1049	4	0	0	0
Sj_Blaster_Recon_5733_MAP_3	LTR	Novel	1731	0	4	0	0
Sj_Blaster_Recon_2210_MAP_3	LTR	Novel	1727	3	1	0	0
Sj_Blaster_Grouper_29711_MAP_4	LTR	Novel	5227	0	0	5	0
Sj_Blaster_Grouper_20136_MAP_3	LTR	Novel	1547	5	0	0	0
Sj_Blaster_Grouper_26344_MAP_3	LTR	Novel	1750	0	0	0	5
Sj_Blaster_Recon_8228_MAP_3	LTR	GYPSY49_AG2p	1885	1	2	1	1
Sj_Blaster_Grouper_22890_MAP_4	LTR	Novel	2039	3	1	0	1
Sj_Blaster_Grouper_34249_MAP_3	LTR	BN000804_1p	6109	1	6	0	0
Sj_Blaster_Recon_8732_MAP_4	LTR	SACI-7_2p	2248	1	1	0	3
Sj_Blaster_Recon_5436_MAP_5	LTR	NONAUT-5_1p	1967	4	1	0	0
Sj_Blaster_Recon_5407_MAP_7	LTR	GYPSY70_AG2p	4591	2	0	0	3
Sj_Blaster_Grouper_18701_MAP_4	LTR	Novel	1333	5	0	0	0
Sj_Blaster_Grouper_17427_MAP_3	LTR	Novel	1179	5	0	0	0
Sj_Blaster_Recon_1782_MAP_4	LTR	Novel	1300	0	5	0	0

Sj_Blaster_Grouper_18735_MAP_4	LTR	Novel	1354	0	5	0	0
Sj_Blaster_Grouper_24813_MAP_3	LTR	Novel	2007	6	0	0	0
Sj_Blaster_Grouper_22915_MAP_5	LTR	BN000804_1p	2038	0	2	3	1
Sj_Blaster_Recon_1663_MAP_3	LTR	Novel	711	0	3	0	3
Sj_Blaster_Recon_5997_MAP_7	LTR	NONAUT-3_1p	2713	1	2	1	2
Sj_Blaster_Recon_5289_MAP_4	LTR	NONAUT-3_1p	1408	2	0	3	0
Sj_Blaster_Grouper_18676_MAP_3	LTR	GYPSY53_AG2p	1146	1	3	5	0
Sj_Blaster_Grouper_34538_MAP_3	LTR	Novel	2921	0	0	6	0
Sj_Blaster_Grouper_31344_MAP_3	LTR	Novel	1171	6	0	0	0
Sj_Blaster_Recon_5438_MAP_3	LTR	GYPSY48_AG2p	2297	0	2	1	0
Sj_Blaster_Grouper_18040_MAP_3	LTR	Novel	1242	6	0	0	0
Sj_Blaster_Grouper_30529_MAP_3	LTR	Novel	3632	0	0	6	0
Sj_Blaster_Recon_6273_MAP_3	LTR	Novel	1979	6	0	0	0
Sj_Blaster_Grouper_26795_MAP_3	LTR	PRIMA4_I	2981	4	0	3	0
Sj_Blaster_Recon_394_MAP_9	LTR	Novel	2443	0	7	0	0
Sj_Blaster_Recon_5451_MAP_4	LTR	SACI-7_2p	791	1	2	4	1
Sj_Blaster_Recon_548_MAP_4	LTR	BN000804_1p	1805	0	1	1	5
Sj_Blaster_Grouper_32434_MAP_3	LTR	Novel	4196	0	0	7	0
Sj_Blaster_Recon_1362_MAP_4	LTR	Novel	1954	0	7	0	0
Sj_Blaster_Grouper_21167_MAP_4	LTR	Novel	1775	0	7	0	0
Sj_Blaster_Recon_3657_MAP_5	LTR	Novel	2462	0	7	0	0
Sj_Blaster_Recon_6354_MAP_8	LTR	Novel	6018	0	2	0	5
Sj_Blaster_Recon_7274_MAP_20	LTR	Novel	1491	0	0	0	8
Sj_Blaster_Grouper_12290_MAP_3	LTR	Novel	765	1	2	2	3
Sj_Blaster_Grouper_23876_MAP_3	LTR	GYPSY70_AG2p	1986	0	2	3	3
Sj_Blaster_Recon_14382_MAP_3	LTR	NONAUT-3_1p	2068	4	0	4	0
Sj_Blaster_Recon_3703_MAP_5	LTR	Novel	2130	0	8	0	0
Sj_Blaster_Grouper_22366_MAP_3	LTR	Novel	1939	8	0	0	0
Sj_Blaster_Grouper_20876_MAP_4	LTR	Novel	1670	0	0	8	0
Sj_Blaster_Grouper_22464_MAP_3	LTR	Novel	1953	3	2	3	1
Sj_Blaster_Recon_13455_MAP_3	LTR	NONAUT-3_1p	1678	2	3	3	1
Sj_Blaster_Grouper_28760_MAP_3	LTR	Novel	3930	0	0	0	9
Sj_Blaster_Grouper_14144_MAP_4	LTR	Novel	883	1	3	2	3
Sj_Blaster_Grouper_8698_MAP_3	LTR	SACI-7_2p	615	3	1	5	1

Sj_Blaster_Recon_11972_MAP_4	LTR	Novel	3999	0	0	0	9
Sj_Blaster_Grouper_1828_MAP_3	LTR	SACI-7_2p	407	3	2	3	2
Sj_Blaster_Grouper_30029_MAP_15	LTR	Novel	6040	0	0	9	0
Sj_Blaster_Grouper_34494_MAP_3	LTR	Novel	9833	0	0	10	0
Sj_Blaster_Recon_11976_MAP_6	LTR	Novel	5355	0	0	10	0
Sj_Blaster_Grouper_23613_MAP_3	LTR	GYPSY70_AG2p	2183	0	3	4	3
Sj_Blaster_Grouper_24108_MAP_3	LTR	NONAUT-5_1p	1794	1	1	1	7
Sj_Blaster_Recon_6621_MAP_3	LTR	SACI-7_2p	1633	6	2	1	1
Sj_Blaster_Grouper_31361_MAP_3	LTR	Novel	2295	0	10	0	0
Sj_Blaster_Recon_15390_MAP_3	LTR	SACI-7_2p	1102	1	5	0	2
Sj_Blaster_Recon_1500_MAP_11	LTR	Novel	2922	0	11	0	0
Sj_Blaster_Recon_8857_MAP_8	LTR	Novel	3011	0	11	0	0
Sj_Blaster_Recon_6681_MAP_10	LTR	Novel	3563	0	0	9	2
Sj_Blaster_Grouper_12186_MAP_3	LTR	NONAUT-3_1p	756	1	3	4	4
Sj_Blaster_Recon_1025_MAP_15	LTR	SACI-5_1p	5794	2	6	3	2
Sj_Blaster_Grouper_24947_MAP_3	LTR	Gypsy1-I_AP_1p	1824	5	7	1	1
Sj_Blaster_Grouper_19375_MAP_6	LTR	Gypsy1-I_AP_1p	1470	7	5	1	1
Sj_Blaster_Recon_5619_MAP_3	LTR	Gypsy9-I_AP_1p	1536	3	5	5	1
Sj_Blaster_Recon_14505_MAP_3	LTR	NONAUT-3_1p	1026	4	3	7	0
Sj_Blaster_Recon_5734_MAP_12	LTR	Novel	4291	0	14	0	0
Sj_Blaster_Grouper_5878_MAP_6	LTR	Gypsy1-SM_LTR	533	0	0	14	0
Sj_Blaster_Recon_8449_MAP_4	LTR	NONAUT-5_1p	4561	7	6	2	4
Sj_Blaster_Grouper_30504_MAP_3	LTR	Novel	3457	15	0	0	0
Sj_Blaster_Recon_1161_MAP_20	LTR	NONAUT-5_1p	2515	10	2	5	2
Sj_Blaster_Recon_6322_MAP_4	LTR	GYPSY58_AG2p	1462	8	5	1	2
Sj_Blaster_Grouper_2869_MAP_4	LTR	GYPSY49_AG2p	439	0	10	4	4
Sj_Blaster_Recon_2509_MAP_3	LTR	Novel	1560	6	6	0	3
Sj_Blaster_Grouper_32620_MAP_3	LTR	Novel	4531	0	16	0	0
Sj_Blaster_Grouper_3483_MAP_20	LTR	NONAUT-3_1p	522	7	1	7	2
Sj_Blaster_Grouper_18578_MAP_3	LTR	SACI-7_2p	1314	0	3	9	8
Sj_Blaster_Recon_2441_MAP_7	LTR	Novel	4473	0	18	0	0
Sj_Blaster_Recon_5293_MAP_4	LTR	NONAUT-3_1p	2259	3	6	5	4
Sj_Blaster_Recon_10338_MAP_3	LTR	GYPSY1_AG2p	932	2	2	13	4
Sj_Blaster_Recon_5466_MAP_3	LTR	SACI-7_2p	3389	4	6	7	6

Sj_Blaster_Recon_9391_MAP_4	LTR	SACI-7_2p	1323	4	3	3	8
Sj_Blaster_Grouper_27092_MAP_4	LTR	Gypsy1-I_AP_1p	2506	9	7	3	2
Sj_Blaster_Grouper_8214_MAP_3	LTR	NONAUT-5_1p	598	3	6	1	6
Sj_Blaster_Recon_7086_MAP_3	LTR	SACI-7_2p	1197	6	10	1	2
Sj_Blaster_Grouper_22436_MAP_4	LTR	Novel	1780	0	0	0	20
Sj_Blaster_Recon_15810_MAP_3	LTR	SACI-7_2p	2034	5	5	0	7
Sj_Blaster_Grouper_32561_MAP_3	LTR	GYPSY53_AG2p	2959	8	13	4	4
Sj_Blaster_Grouper_32211_MAP_18	LTR	Novel	8969	21	0	0	0
Sj_Blaster_Grouper_23674_MAP_7	LTR	Novel	1058	0	10	8	5
Sj_Blaster_Grouper_26119_MAP_3	LTR	BEL1-I_AG-ORF1p	2803	0	4	1	17
Sj_Blaster_Grouper_11299_MAP_3	LTR	SACI-7_2p	715	6	23	4	4
Sj_Blaster_Recon_9241_MAP_5	LTR	SACI-7_2p	2869	3	6	2	20
Sj_Blaster_Grouper_16874_MAP_3	LTR	Novel	1115	0	7	1	14
Sj_Blaster_Grouper_14799_MAP_20	LTR	GYPSY2_AGp	1000	2	10	5	7
Sj_Blaster_Grouper_30699_MAP_5	LTR	GYPSY70_AG2p	1240	2	12	3	7
Sj_Blaster_Grouper_15204_MAP_3	LTR	GYPSY1_AG2p	961	2	10	5	7
Sj_Blaster_Grouper_27798_MAP_5	LTR	Novel	3470	2	0	0	20
Sj_Blaster_Grouper_28918_MAP_4	LTR	BEL17_AGp	3963	0	4	0	19
Sj_Blaster_Recon_6418_MAP_3	LTR	SACI-7_2p	547	2	6	10	1
Sj_Blaster_Grouper_22956_MAP_3	LTR	GYPSY70_AG2p	1152	14	2	8	3
Sj_Blaster_Recon_5452_MAP_5	LTR	SACI-7_2p	1765	4	4	3	12
Sj_Blaster_Grouper_6350_MAP_3	LTR	SACI-7_2p	539	4	33	7	1
Sj_Blaster_Grouper_31297_MAP_6	LTR	GYPSY1_AG2p	1366	3	0	6	11
Sj_Blaster_Grouper_13402_MAP_20	LTR	NONAUT-3_1p	1040	11	4	7	3
Sj_Blaster_Recon_1023_MAP_3	LTR	SACI-5_1p	2529	1	8	3	8
Sj_Blaster_Grouper_14522_MAP_3	LTR	SACI-7_2p	909	4	3	13	8
Sj_Blaster_Grouper_33448_MAP_3	LTR	Novel	12094	24	1	0	0
Sj_Blaster_Recon_2201_MAP_3	LTR	GYPSY49_AG2p	912	7	2	5	13
Sj_Blaster_Recon_15359_MAP_3	LTR	GYPSY6-I_CB_ORF	2411	5	9	0	142
Sj_Blaster_Grouper_30111_MAP_3	LTR	SACI-7_2p	1413	11	4	12	2
Sj_Blaster_Grouper_23900_MAP_6	LTR	GYPSY63_AGp	2236	14	3	4	8
Sj_Blaster_Recon_5092_MAP_5	LTR	GYPSY21_AGp	2714	19	4	5	4
Sj_Blaster_Grouper_23248_MAP_3	LTR	GYPSY53_AG2p	1154	1	7	14	8
Sj_Blaster_Grouper_28734_MAP_4	LTR	GYPSY48_AG2p	1467	15	1	11	2

Sj_Blaster_Grouper_21882_MAP_4	LTR	GYPSY64_AGP	1801	0	0	0	28
Sj_Blaster_Grouper_14673_MAP_3	LTR	NONAUT-5_1p	920	14	7	6	6
Sj_Blaster_Grouper_17378_MAP_5	LTR	NONAUT-5_1p	1175	0	0	0	28
Sj_Blaster_Grouper_28607_MAP_3	LTR	GYPSY1_AG2p	1918	4	7	12	8
Sj_Blaster_Grouper_17689_MAP_8	LTR	SACI-7_2p	1227	7	9	5	8
Sj_Blaster_Grouper_33291_MAP_3	LTR	Novel	1031	0	15	8	9
Sj_Blaster_Recon_6303_MAP_7	LTR	NONAUT-3_1p	3733	8	8	4	13
Sj_Blaster_Recon_1139_MAP_3	LTR	NONAUT-5_1p	478	5	31	3	7
Sj_Blaster_Recon_2659_MAP_11	LTR	GYPSY49_AG2p	4404	8	5	17	4
Sj_Blaster_Grouper_1177_MAP_3	LTR	GYPSY1_AG2p	385	5	12	12	5
Sj_Blaster_Grouper_25703_MAP_3	LTR	Novel	1202	0	18	9	8
Sj_Blaster_Recon_10515_MAP_4	LTR	SACI-5_1p	1666	19	6	5	35
Sj_Blaster_Grouper_26585_MAP_3	LTR	Novel	1388	0	18	8	9
Sj_Blaster_Grouper_25517_MAP_3	LTR	Novel	1465	0	20	11	6
Sj_Blaster_Grouper_6787_MAP_4	LTR	Novel	552	0	0	34	0
Sj_Blaster_Recon_284_MAP_7	LTR	GYPSY1_AG2p	2462	3	2	15	10
Sj_Blaster_Recon_5717_MAP_4	LTR	Gypsy9-I_AP_1p	1439	45	3	4	7
Sj_Blaster_Grouper_25845_MAP_7	LTR	SACI-7_2p	2009	7	17	6	9
Sj_Blaster_Grouper_21598_MAP_3	LTR	NONAUT-3_1p	1210	19	10	5	6
Sj_Blaster_Recon_5458_MAP_3	LTR	GYPSY2_AGp	2861	6	12	9	8
Sj_Blaster_Grouper_31599_MAP_4	LTR	Gypsy1-SM_LTR	1978	20	3	7	11
Sj_Blaster_Recon_568_MAP_8	LTR	GYPSY21_AGp	2951	16	10	5	8
Sj_Blaster_Grouper_29541_MAP_3	LTR	Novel	1527	0	21	15	6
Sj_Blaster_Grouper_25356_MAP_3	LTR	Novel	1528	0	19	13	9
Sj_Blaster_Grouper_25619_MAP_3	LTR	GYPSY63_AGp	2657	28	5	7	5
Sj_Blaster_Grouper_31006_MAP_3	LTR	SACI-7_2p	2659	16	10	8	11
Sj_Blaster_Grouper_22731_MAP_4	LTR	Novel	1499	0	18	14	10
Sj_Blaster_Grouper_27284_MAP_3	LTR	Novel	1230	0	23	15	6
Sj_Blaster_Grouper_33336_MAP_3	LTR	Novel	1332	0	21	16	7
Sj_Blaster_Grouper_9448_MAP_20	LTR	GYPSY60_AG2p	742	1	0	1	39
Sj_Blaster_Grouper_30491_MAP_3	LTR	Novel	1534	0	21	16	8
Sj_Blaster_Recon_2286_MAP_4	LTR	GYPSY19_AGp	3465	6	5	18	18
Sj_Blaster_Grouper_18481_MAP_5	LTR	Novel	1307	0	0	12	30
Sj_Blaster_Recon_8177_MAP_9	LTR	Novel	1906	15	0	26	2

Sj_Blaster_Grouper_25156_MAP_3	LTR	NONAUT-3_1p	1360	23	14	7	4
Sj_Blaster_Recon_616_MAP_3	LTR	GYPSY2_AGp	1031	46	4	8	5
Sj_Blaster_Recon_7194_MAP_14	LTR	Gypsy1-I_AP_1p	3993	13	23	7	5
Sj_Blaster_Grouper_31549_MAP_3	LTR	GYPSY1_AG2p	1573	12	12	16	14
Sj_Blaster_Grouper_11107_MAP_3	LTR	SACI-7_2p	706	11	18	2	21
Sj_Blaster_Grouper_25528_MAP_3	LTR	Novel	1401	0	27	19	4
Sj_Blaster_Recon_10405_MAP_3	LTR	SACI-7_2p	1833	5	9	8	28
Sj_Blaster_Grouper_33263_MAP_3	LTR	Novel	1672	0	29	15	7
Sj_Blaster_Recon_10339_MAP_4	LTR	GYPSY1_AG1p	1012	12	41	4	11
Sj_Blaster_Recon_7928_MAP_7	LTR	GYPSY1_AG2p	3727	17	30	6	8
Sj_Blaster_Recon_4199_MAP_4	LTR	BN000804_1p	1257	30	27	14	3
Sj_Blaster_Grouper_25734_MAP_3	LTR	Novel	1731	0	22	19	11
Sj_Blaster_Recon_9243_MAP_3	LTR	GYPSY1_AG2p	2279	18	21	22	4
Sj_Blaster_Grouper_24451_MAP_3	LTR	Novel	1653	0	29	21	7
Sj_Blaster_Grouper_11303_MAP_3	LTR	SACI-7_2p	716	21	23	15	9
Sj_Blaster_Grouper_6358_MAP_20	LTR	GYPSY1_AG2p	854	15	12	23	11
Sj_Blaster_Grouper_11760_MAP_3	LTR	Novel	738	22	34	2	7
Sj_Blaster_Grouper_29207_MAP_3	LTR	NONAUT-5_1p	1488	10	23	13	20
Sj_Blaster_Grouper_27314_MAP_3	LTR	Novel	1679	0	32	19	9
Sj_Blaster_Grouper_23834_MAP_3	LTR	Novel	2212	4	27	24	5
Sj_Blaster_Grouper_24421_MAP_3	LTR	NONAUT-3_1p	1190	24	21	8	8
Sj_Blaster_Grouper_27032_MAP_3	LTR	Novel	1882	0	29	22	11
Sj_Blaster_Grouper_29084_MAP_3	LTR	Novel	1979	1	30	20	11
Sj_Blaster_Grouper_5087_MAP_3	LTR	SACI-5_1p	503	68	14	6	10
Sj_Blaster_Recon_6068_MAP_16	LTR	Novel	3056	6	33	19	6
Sj_Blaster_Recon_7262_MAP_7	LTR	Novel	3059	6	0	16	42
Sj_Blaster_Grouper_28749_MAP_3	LTR	NONAUT-5_1p	2757	10	23	15	23
Sj_Blaster_Grouper_23240_MAP_3	LTR	Novel	1925	0	31	25	9
Sj_Blaster_Grouper_20079_MAP_3	LTR	SACI-7_2p	1539	9	16	33	18
Sj_Blaster_Grouper_29247_MAP_3	LTR	Novel	1399	0	38	23	8
Sj_Blaster_Recon_2310_MAP_3	LTR	GYPSY1_AG2p	906	19	29	7	25
Sj_Blaster_Recon_11477_MAP_3	LTR	NONAUT-5_1p	1575	22	25	15	17
Sj_Blaster_Grouper_29600_MAP_7	LTR	NONAUT-5_1p	1293	14	22	14	19
Sj_Blaster_Grouper_31351_MAP_3	LTR	GYPSY1_AG1p	1178	32	21	17	4

Sj_Blaster_Grouper_24250_MAP_5	LTR	SACI-7_2p	2326	19	15	13	24
Sj_Blaster_Recon_7685_MAP_3	LTR	Novel	894	17	39	8	8
Sj_Blaster_Recon_10993_MAP_5	LTR	Novel	2660	18	43	15	9
Sj_Blaster_Recon_5406_MAP_14	LTR	SACI-7_2p	4281	35	21	11	8
Sj_Blaster_Recon_89_MAP_5	LTR	GYPSY49_AG2p	6698	10	20	19	19
Sj_Blaster_Grouper_24285_MAP_3	LTR	Novel	2014	0	41	25	9
Sj_Blaster_Grouper_27206_MAP_4	LTR	Novel	1680	2	35	23	14
Sj_Blaster_Recon_8834_MAP_4	LTR	NONAUT-5_1p	1000	6	30	10	27
Sj_Blaster_Grouper_29631_MAP_4	LTR	BN000804_1p	1782	27	41	9	3
Sj_Blaster_Grouper_22931_MAP_4	LTR	Novel	2008	0	38	22	14
Sj_Blaster_Recon_9418_MAP_7	LTR	GYPSY52_AG2p	2207	27	14	20	19
Sj_Blaster_Recon_9229_MAP_6	LTR	Novel	2149	58	2	16	11
Sj_Blaster_Grouper_23972_MAP_3	LTR	Novel	2220	40	0	15	25
Sj_Blaster_Recon_10934_MAP_4	LTR	SACI-7_2p	827	23	32	25	12
Sj_Blaster_Recon_9958_MAP_3	LTR	NONAUT-3_1p	3018	10	82	6	15
Sj_Blaster_Grouper_34280_MAP_4	LTR	SACI-7_2p	2895	19	23	11	31
Sj_Blaster_Grouper_12166_MAP_3	LTR	NONAUT-5_1p	758	25	24	5	42
Sj_Blaster_Recon_86_MAP_20	LTR	BN000804_1p	7711	14	23	16	9
Sj_Blaster_Recon_5620_MAP_3	LTR	Gypsy9-I_AP_1p	1977	42	12	49	14
Sj_Blaster_Recon_6655_MAP_4	LTR	ATCOPIA24I	1500	0	38	0	38
Sj_Blaster_Grouper_34264_MAP_3	LTR	SACI-7_2p	2978	20	23	13	30
Sj_Blaster_Grouper_32757_MAP_3	LTR	Novel	2713	26	35	5	14
Sj_Blaster_Recon_15701_MAP_5	LTR	NONAUT-5_1p	2338	10	24	5	26
Sj_Blaster_Recon_7330_MAP_6	LTR	Novel	1551	8	51	0	46
Sj_Blaster_Grouper_23694_MAP_3	LTR	Novel	2085	47	0	16	29
Sj_Blaster_Grouper_31806_MAP_5	LTR	Novel	1634	0	50	25	19
Sj_Blaster_Grouper_17365_MAP_20	LTR	GYPSY64_AGP	1210	24	29	25	19
Sj_Blaster_Grouper_18030_MAP_3	LTR	NONAUT-5_1p	1248	17	41	6	34
Sj_Blaster_Recon_8163_MAP_4	LTR	GYPSY1_AG2p	1948	49	44	16	9
Sj_Blaster_Grouper_29565_MAP_3	LTR	Novel	2755	0	43	35	15
Sj_Blaster_Recon_6907_MAP_4	LTR	NONAUT-5_1p	2662	26	31	15	17
Sj_Blaster_Grouper_34480_MAP_4	LTR	Novel	3478	0	49	27	18
Sj_Blaster_Recon_5288_MAP_4	LTR	NONAUT-3_1p	1835	9	21	15	48
Sj_Blaster_Grouper_32314_MAP_3	LTR	Novel	3285	39	36	3	10

Sj_Blaster_Grouper_28268_MAP_3	LTR	BN000804_1p	2229	12	23	13	32
Sj_Blaster_Grouper_22929_MAP_3	LTR	NONAUT-3_1p	2049	11	35	30	20
Sj_Blaster_Grouper_27939_MAP_3	LTR	BN000804_1p	2035	12	23	13	32
Sj_Blaster_Grouper_14886_MAP_3	LTR	;YPSY5-I_CB_ORF	937	19	36	16	29
Sj_Blaster_Grouper_28163_MAP_3	LTR	SACI-7_2p	2890	24	12	37	20
Sj_Blaster_Grouper_31607_MAP_16	LTR	Novel	2316	0	50	36	13
Sj_Blaster_Recon_9125_MAP_3	LTR	Novel	2211	0	67	1	26
Sj_Blaster_Recon_8433_MAP_5	LTR	NONAUT-5_1p	3240	24	25	8	26
Sj_Blaster_Recon_9253_MAP_6	LTR	Novel	1511	0	11	0	92
Sj_Blaster_Recon_7488_MAP_3	LTR	GYPSY2_AGp	1429	41	26	29	22
Sj_Blaster_Grouper_29083_MAP_3	LTR	SACI-7_2p	4337	26	25	20	32
Sj_Blaster_Grouper_31154_MAP_3	LTR	GYPSY70_AG2p	1359	18	44	23	16
Sj_Blaster_Grouper_29053_MAP_8	LTR	Novel	2479	0	58	37	14
Sj_Blaster_Grouper_30632_MAP_3	LTR	Novel	2727	0	63	39	11
Sj_Blaster_Grouper_31929_MAP_3	LTR	Novel	3101	0	58	36	17
Sj_Blaster_Recon_5292_MAP_4	LTR	NONAUT-5_1p	1564	26	38	23	23
Sj_Blaster_Grouper_33922_MAP_3	LTR	Novel	3180	0	55	39	18
Sj_Blaster_Recon_2515_MAP_5	LTR	NONAUT-5_1p	2979	14	21	15	41
Sj_Blaster_Recon_673_MAP_3	LTR	SACI-7_2p	1035	36	65	7	27
Sj_Blaster_Grouper_12264_MAP_3	LTR	GYPSY52_AG2p	762	25	68	14	23
Sj_Blaster_Grouper_30829_MAP_3	LTR	Gypsy1-I_AP_1p	1379	21	48	19	16
Sj_Blaster_Grouper_30846_MAP_3	LTR	Novel	2696	0	57	41	18
Sj_Blaster_Recon_6636_MAP_14	LTR	Novel	5536	41	41	14	35
Sj_Blaster_Grouper_21343_MAP_4	LTR	Novel	1542	11	3	134	9
Sj_Blaster_Grouper_30907_MAP_3	LTR	Novel	1139	0	65	21	46
Sj_Blaster_Recon_2309_MAP_4	LTR	GYPSY1_AG2p	1471	62	25	7	24
Sj_Blaster_Grouper_30728_MAP_3	LTR	GYPSY70_AG2p	2539	28	39	34	30
Sj_Blaster_Grouper_32908_MAP_3	LTR	Novel	2806	0	67	40	19
Sj_Blaster_Grouper_8325_MAP_4	LTR	SACI-7_2p	604	93	29	12	28
Sj_Blaster_Recon_1351_MAP_5	LTR	SACI-5_1p	1361	133	76	17	3
Sj_Blaster_Grouper_31573_MAP_3	LTR	SACI-5_1p	1091	133	76	17	3
Sj_Blaster_Grouper_16815_MAP_20	LTR	NONAUT-3_1p	1237	28	36	11	69
Sj_Blaster_Recon_480_MAP_4	LTR	GYPSY49_AG1p	1893	40	29	18	38
Sj_Blaster_Grouper_30595_MAP_3	LTR	Novel	3256	14	85	9	16

Sj_Blaster_Grouper_21034_MAP_10	LTR	Novel	1942	73	24	17	70
Sj_Blaster_Grouper_32921_MAP_3	LTR	Novel	5968	16	21	26	41
Sj_Blaster_Grouper_29166_MAP_4	LTR	Novel	2328	4	81	23	33
Sj_Blaster_Recon_3046_MAP_3	LTR	SACI-7_2p	879	69	37	18	28
Sj_Blaster_Recon_1160_MAP_14	LTR	NONAUT-5_1p	4830	5	1	4	115
Sj_Blaster_Grouper_29561_MAP_3	LTR	Novel	3106	15	88	8	16
Sj_Blaster_Recon_5287_MAP_3	LTR	NONAUT-3_1p	1906	56	30	20	20
Sj_Blaster_Grouper_30629_MAP_14	LTR	GYPSY1_AG2p	1249	41	35	36	29
Sj_Blaster_Grouper_34461_MAP_3	LTR	Novel	3821	50	63	5	15
Sj_Blaster_Recon_16464_MAP_4	LTR	SACI-7_2p	668	54	24	21	25
Sj_Blaster_Grouper_34646_MAP_11	LTR	Novel	3796	0	73	51	17
Sj_Blaster_Grouper_34511_MAP_5	LTR	Novel	2587	0	77	48	17
Sj_Blaster_Grouper_21480_MAP_3	LTR	NONAUT-5_1p	1773	49	44	22	24
Sj_Blaster_Recon_7809_MAP_3	LTR	Novel	792	0	36	0	108
Sj_Blaster_Grouper_20889_MAP_8	LTR	Novel	1684	57	57	17	21
Sj_Blaster_Grouper_3463_MAP_20	LTR	Novel	475	44	58	16	38
Sj_Blaster_Grouper_30718_MAP_3	LTR	GYPSY70_AG2p	1555	52	35	39	30
Sj_Blaster_Grouper_30716_MAP_3	LTR	GYPSY70_AG2p	1572	52	35	39	31
Sj_Blaster_Grouper_20656_MAP_5	LTR	NONAUT-5_1p	1314	58	22	31	31
Sj_Blaster_Recon_5246_MAP_3	LTR	Gypsy1-I_AP_1p	710	35	75	32	27
Sj_Blaster_Grouper_32524_MAP_4	LTR	Novel	3779	52	65	6	21
Sj_Blaster_Grouper_18839_MAP_20	LTR	Novel	2157	25	117	72	11
Sj_Blaster_Recon_334_MAP_20	LTR	NONAUT-5_1p	2553	77	62	16	20
Sj_Blaster_Grouper_30651_MAP_3	LTR	SACI-7_2p	1893	52	38	39	31
Sj_Blaster_Recon_1247_MAP_16	LTR	GYPSY48_AG2p	4393	37	27	66	31
Sj_Blaster_Recon_9294_MAP_5	LTR	Novel	2228	0	1139	0	27
Sj_Blaster_Recon_16534_MAP_3	LTR	SACI-7_1p	843	44	25	63	37
Sj_Blaster_Grouper_29192_MAP_3	LTR	Novel	4267	53	71	6	21
Sj_Blaster_Grouper_34643_MAP_3	LTR	SACI-7_2p	2090	29	54	29	33
Sj_Blaster_Recon_87_MAP_20	LTR	SACI-7_2p	3402	40	19	49	48
Sj_Blaster_Grouper_28565_MAP_6	LTR	SACI-7_2p	2693	16	34	51	52
Sj_Blaster_Grouper_17399_MAP_10	LTR	NONAUT-5_1p	1203	59	70	25	19
Sj_Blaster_Grouper_22550_MAP_3	LTR	BN000804_1p	1350	56	71	21	19
Sj_Blaster_Grouper_28462_MAP_3	LTR	GYPSY49_AG1p	2550	77	35	31	60

Sj_Blaster_Grouper_30545_MAP_5	LTR	GYPSY70_AG2p	1600	53	42	41	36
Sj_Blaster_Grouper_26014_MAP_12	LTR	Novel	2903	0	93	57	19
Sj_Blaster_Grouper_31988_MAP_3	LTR	SACI-7_2p	1729	69	30	27	32
Sj_Blaster_Grouper_35029_MAP_3	LTR	Novel	5253	57	77	6	21
Sj_Blaster_Grouper_30806_MAP_3	LTR	GYPSY70_AG2p	1755	42	54	36	45
Sj_Blaster_Grouper_30613_MAP_3	LTR	GYPSY48_AG1p	1183	11	132	9	25
Sj_Blaster_Grouper_29296_MAP_3	LTR	GYPSY49_AG1p	3130	78	35	37	59
Sj_Blaster_Grouper_26807_MAP_3	LTR	NONAUT-3_1p	2353	47	44	28	64
Sj_Blaster_Grouper_18797_MAP_3	LTR	GYPSY49_AG1p	1348	101	35	19	20
Sj_Blaster_Grouper_32200_MAP_3	LTR	Novel	3058	1	97	64	21
Sj_Blaster_Grouper_12214_MAP_4	LTR	NONAUT-3_1p	761	95	61	33	19
Sj_Blaster_Recon_2837_MAP_20	LTR	NONAUT-5_1p	8281	62	59	65	24
Sj_Blaster_Grouper_10558_MAP_8	LTR	NONAUT-3_1p	686	92	64	33	23
Sj_Blaster_Grouper_31611_MAP_3	LTR	Novel	3192	0	113	41	49
Sj_Blaster_Grouper_26198_MAP_3	LTR	NONAUT-5_1p	1246	48	94	38	47
Sj_Blaster_Grouper_21866_MAP_3	LTR	Gypsy9-I_AP_1p	1617	53	75	29	43
Sj_Blaster_Grouper_25936_MAP_3	LTR	BN000804_1p	2747	45	26	60	26
Sj_Blaster_Grouper_29930_MAP_4	LTR	Novel	2018	2	100	38	66
Sj_Blaster_Grouper_21046_MAP_3	LTR	BN000804_1p	1692	67	106	12	26
Sj_Blaster_Grouper_34287_MAP_8	LTR	Novel	3727	0	108	72	23
Sj_Blaster_Recon_11924_MAP_3	LTR	NONAUT-5_1p	2200	53	70	80	36
Sj_Blaster_Recon_4870_MAP_3	LTR	GYPSY1_AG2p	1114	49	129	36	26
Sj_Blaster_Grouper_17381_MAP_3	LTR	NONAUT-3_1p	1174	38	35	37	59
Sj_Blaster_Grouper_30620_MAP_3	LTR	Novel	4930	71	94	8	25
Sj_Blaster_Recon_6959_MAP_4	LTR	Novel	1931	0	42	0	318
Sj_Blaster_Grouper_22191_MAP_3	LTR	Novel	1894	44	37	17	126
Sj_Blaster_Recon_9665_MAP_4	LTR	NONAUT-5_1p	1650	69	46	73	58
Sj_Blaster_Grouper_21234_MAP_3	LTR	SACI-7_2p	1734	113	53	77	49
Sj_Blaster_Grouper_17213_MAP_4	LTR	Gypsy1-I_AP_1p	1153	74	112	26	41
Sj_Blaster_Grouper_16334_MAP_5	LTR	GYPSY2_AGp	1063	67	123	36	34
Sj_Blaster_Grouper_17181_MAP_4	LTR	GYPSY2_AGp	1172	122	65	34	39
Sj_Blaster_Grouper_24932_MAP_3	LTR	NONAUT-3_1p	2476	89	117	38	27
Sj_Blaster_Grouper_21675_MAP_14	LTR	NONAUT-3_1p	1857	50	47	94	40
Sj_Blaster_Grouper_34509_MAP_3	LTR	NONAUT-5_1p	3801	101	54	26	42

Sj_Blaster_Recon_2661_MAP_3	LTR	Novel	1577	143	59	49	27
Sj_Blaster_Recon_4052_MAP_3	LTR	GYPSY49_AG2p	3025	87	121	48	29
Sj_Blaster_Grouper_31936_MAP_3	LTR	Novel	7627	77	102	12	32
Sj_Blaster_Recon_6635_MAP_3	LTR	SACI-7_1p	804	77	85	40	34
Sj_Blaster_Recon_6980_MAP_20	LTR	NONAUT-5_1p	3527	64	61	75	49
Sj_Blaster_Recon_5036_MAP_6	LTR	Novel	1664	34	100	0	74
Sj_Blaster_Grouper_5858_MAP_5	LTR	Novel	522	56	150	17	50
Sj_Blaster_Grouper_17153_MAP_10	LTR	Novel	1206	69	73	52	53
Sj_Blaster_Grouper_19476_MAP_3	LTR	SACI-7_2p	1373	103	109	52	24
Sj_Blaster_Recon_1352_MAP_13	LTR	GYPSY2_AGp	4721	71	46	68	54
Sj_Blaster_Grouper_12796_MAP_4	LTR	GYPSY5-I_CB_ORF	806	31	52	82	113
Sj_Blaster_Grouper_20595_MAP_3	LTR	Novel	1637	4	16	8	266
Sj_Blaster_Grouper_30094_MAP_3	LTR	SACI-7_2p	1095	51	121	23	73
Sj_Blaster_Recon_2014_MAP_3	LTR	SACI-7_1p	1864	78	76	29	85
Sj_Blaster_Grouper_7986_MAP_4	LTR	NONAUT-5_1p	590	85	78	52	39
Sj_Blaster_Grouper_18071_MAP_20	LTR	Novel	1844	189	42	27	114
Sj_Blaster_Recon_12968_MAP_3	LTR	SACI-7_2p	1920	109	61	47	43
Sj_Blaster_Grouper_24468_MAP_4	LTR	Novel	2221	1	147	37	102
Sj_Blaster_Recon_8982_MAP_10	LTR	Novel	6536	81	117	13	35
Sj_Blaster_Grouper_34324_MAP_3	LTR	Novel	6455	77	126	12	35
Sj_Blaster_Grouper_29466_MAP_3	LTR	GYPSY2_AGp	1362	109	63	64	46
Sj_Blaster_Grouper_19791_MAP_3	LTR	NONAUT-5_1p	1497	50	42	78	68
Sj_Blaster_Grouper_32382_MAP_12	LTR	NONAUT-3_1p	1208	105	51	70	64
Sj_Blaster_Grouper_29260_MAP_3	LTR	NONAUT-5_1p	2864	98	75	61	46
Sj_Blaster_Grouper_33486_MAP_3	LTR	Novel	8748	98	119	9	31
Sj_Blaster_Grouper_30720_MAP_3	LTR	NONAUT-3_1p	3381	69	115	44	48
Sj_Blaster_Grouper_29164_MAP_3	LTR	GYPSY49_AG1p	1787	149	75	25	33
Sj_Blaster_Recon_5355_MAP_10	LTR	Saci-2_I	2004	98	121	61	23
Sj_Blaster_Grouper_31099_MAP_3	LTR	Novel	6663	89	123	12	37
Sj_Blaster_Grouper_20373_MAP_15	LTR	Novel	2265	69	49	22	168
Sj_Blaster_Grouper_32074_MAP_3	LTR	Novel	2634	8	151	39	95
Sj_Blaster_Grouper_33009_MAP_3	LTR	Novel	2128	1	143	41	116
Sj_Blaster_Grouper_8577_MAP_12	LTR	NONAUT-3_1p	646	109	79	43	31
Sj_Blaster_Grouper_24527_MAP_15	LTR	Novel	2414	4	150	46	103

Sj_Blaster_Grouper_28395_MAP_3	LTR	Novel	3731	79	197	12	14
Sj_Blaster_Recon_18130_MAP_3	LTR	NONAUT-5_1p	1991	68	22	104	144
Sj_Blaster_Grouper_30607_MAP_3	LTR	SACI-7_2p	1219	100	81	68	44
Sj_Blaster_Grouper_31241_MAP_5	LTR	Novel	6829	131	96	37	13
Sj_Blaster_Grouper_15919_MAP_20	LTR	GYPSY60_AG2p	1131	90	102	41	71
Sj_Blaster_Grouper_26495_MAP_3	LTR	SACI-5_1p	2296	255	180	18	35
Sj_Blaster_Grouper_22869_MAP_3	LTR	NONAUT-5_1p	1616	48	115	78	66
Sj_Blaster_Grouper_20053_MAP_3	LTR	NONAUT-3_1p	1378	65	105	70	75
Sj_Blaster_Grouper_29022_MAP_6	LTR	BEL14_AGp	4517	179	141	40	23
Sj_Blaster_Grouper_30951_MAP_3	LTR	SACI-7_2p	1099	102	187	30	35
Sj_Blaster_Grouper_28861_MAP_5	LTR	SACI-7_2p	3963	49	52	80	127
Sj_Blaster_Recon_6525_MAP_7	LTR	BEL17_AGp	3618	91	104	37	64
Sj_Blaster_Recon_6634_MAP_3	LTR	GYPSY53_AG2p	1896	83	80	62	75
Sj_Blaster_Recon_5731_MAP_6	LTR	Novel	3117	0	99	3	251
Sj_Blaster_Recon_10527_MAP_3	LTR	GYPSM1_I_1p	1068	126	122	35	37
Sj_Blaster_Recon_537_MAP_4	LTR	GYPSY21_AGp	2291	75	101	65	75
Sj_Blaster_Recon_7561_MAP_6	LTR	GYPSY2_AGp	2100	110	148	47	57
Sj_Blaster_Grouper_31812_MAP_7	LTR	Novel	2432	22	195	42	79
Sj_Blaster_Grouper_18366_MAP_13	LTR	NONAUT-3_1p	1391	123	64	99	82
Sj_Blaster_Recon_1616_MAP_10	LTR	BEL1-I_AG-ORF1p	5166	173	81	40	59
Sj_Blaster_Grouper_24574_MAP_4	LTR	GYPSY53_AG2p	1210	115	93	103	61
Sj_Blaster_Recon_8589_MAP_8	LTR	Novel	2480	12	86	2	326
Sj_Blaster_Grouper_7526_MAP_3	LTR	SACI-7_2p	577	184	136	41	34
Sj_Blaster_Grouper_20550_MAP_3	LTR	Novel	1597	72	176	54	61
Sj_Blaster_Grouper_19873_MAP_11	LTR	Novel	1508	58	108	50	118
Sj_Blaster_Recon_6447_MAP_20	LTR	BN000804_1p	3372	200	123	46	17
Sj_Blaster_Grouper_17499_MAP_4	LTR	NONAUT-5_1p	1189	45	44	325	108
Sj_Blaster_Grouper_20734_MAP_20	LTR	Novel	1732	61	103	53	124
Sj_Blaster_Grouper_25289_MAP_3	LTR	Novel	2614	26	5	288	99
Sj_Blaster_Recon_8399_MAP_4	LTR	GYPSY1_AG2p	2937	146	111	72	45
Sj_Blaster_Grouper_4915_MAP_3	LTR	Novel	496	226	105	63	78
Sj_Blaster_Grouper_35054_MAP_3	LTR	Novel	10719	128	164	13	43
Sj_Blaster_Grouper_30283_MAP_5	LTR	NONAUT-3_1p	1908	93	82	90	91
Sj_Blaster_Grouper_29068_MAP_3	LTR	SACI-7_2p	1432	174	133	80	56

Sj_Blaster_Recon_9176_MAP_3	LTR	SACI-7_2p	2450	165	173	36	50
Sj_Blaster_Grouper_27593_MAP_3	LTR	NONAUT-3_1p	2135	91	109	52	107
Sj_Blaster_Recon_5295_MAP_3	LTR	SACI-7_2p	1871	170	89	69	56
Sj_Blaster_Grouper_23162_MAP_4	LTR	GYPSY21_AGp	1957	115	86	97	71
Sj_Blaster_Grouper_30126_MAP_5	LTR	NONAUT-5_1p	1721	65	57	105	116
Sj_Blaster_Grouper_21773_MAP_5	LTR	Novel	1855	97	176	61	72
Sj_Blaster_Recon_12773_MAP_11	LTR	GYPSY1_AG2p	1266	108	145	84	75
Sj_Blaster_Grouper_30929_MAP_3	LTR	SACI-7_2p	1248	120	125	60	84
Sj_Blaster_Grouper_32373_MAP_3	LTR	NONAUT-3_1p	1288	125	88	104	109
Sj_Blaster_Piler_624.114_MAP_5	LTR	NONAUT-5_1p	1922	151	169	45	77
Sj_Blaster_Recon_7010_MAP_11	LTR	Novel	1782	0	74	13	280
Sj_Blaster_Grouper_30476_MAP_3	LTR	NONAUT-3_1p	2560	96	114	137	57
Sj_Blaster_Grouper_20508_MAP_3	LTR	Novel	1599	0	66	13	291
Sj_Blaster_Grouper_21543_MAP_3	LTR	NONAUT-3_1p	1779	127	205	62	64
Sj_Blaster_Recon_9199_MAP_5	LTR	Novel	1735	0	505	3	286
Sj_Blaster_Recon_215_MAP_20	LTR	NONAUT-5_1p	2552	105	185	63	63
Sj_Blaster_Recon_928_MAP_20	LTR	GYPSM1_I_1p	3738	375	154	28	39
Sj_Blaster_Grouper_21981_MAP_8	LTR	NONAUT-5_1p	1933	167	131	68	45
Sj_Blaster_Grouper_29625_MAP_3	LTR	Novel	1887	8	221	51	172
Sj_Blaster_Grouper_22786_MAP_3	LTR	NONAUT-5_1p	2009	114	92	121	89
Sj_Blaster_Grouper_18517_MAP_3	LTR	NONAUT-5_1p	1314	151	92	60	118
Sj_Blaster_Grouper_21115_MAP_4	LTR	GYPSY49_AG1p	1707	175	115	81	88
Sj_Blaster_Recon_9292_MAP_5	LTR	Novel	1700	32	272	35	231
Sj_Blaster_Grouper_31162_MAP_6	LTR	Novel	1374	132	0	313	0
Sj_Blaster_Grouper_28312_MAP_3	LTR	SACI-7_2p	3673	196	417	100	53
Sj_Blaster_Grouper_26741_MAP_3	LTR	NONAUT-5_1p	2420	107	89	104	122
Sj_Blaster_Grouper_25063_MAP_3	LTR	NONAUT-5_1p	1191	149	124	85	105
Sj_Blaster_Grouper_16160_MAP_3	LTR	Novel	1045	160	479	43	74
Sj_Blaster_Grouper_31005_MAP_10	LTR	Novel	3875	5	258	94	161
Sj_Blaster_Recon_30_MAP_20	LTR	GYPSY1_AG2p	3389	142	194	92	87
Sj_Blaster_Grouper_27425_MAP_4	LTR	NONAUT-5_1p	3262	127	94	123	123
Sj_Blaster_Recon_969_MAP_20	LTR	NONAUT-3_1p	4469	103	149	146	95
Sj_Blaster_Recon_12790_MAP_4	LTR	NONAUT-5_1p	1741	105	147	112	123
Sj_Blaster_Grouper_23922_MAP_3	LTR	SACI-7_2p	1705	167	306	86	58

Sj_Blaster_Grouper_3094_MAP_4	LTR	Novel	446	295	378	54	51
Sj_Blaster_Grouper_22384_MAP_6	LTR	Novel	1978	133	284	91	77
Sj_Blaster_Grouper_31631_MAP_3	LTR	SACI-5_1p	1118	427	318	46	17
Sj_Blaster_Grouper_29489_MAP_3	LTR	NONAUT-5_1p	1635	172	60	145	175
Sj_Blaster_Grouper_26558_MAP_3	LTR	NONAUT-5_1p	2939	108	119	140	127
Sj_Blaster_Grouper_24120_MAP_4	LTR	BEL1-I_AG-ORF1p	2105	273	493	41	60
Sj_Blaster_Recon_8238_MAP_6	LTR	SACI-7_2p	3028	191	252	103	76
Sj_Blaster_Grouper_21239_MAP_3	LTR	Novel	1732	207	323	86	73
Sj_Blaster_Recon_10345_MAP_4	LTR	SACI-7_2p	1964	170	203	121	98
Sj_Blaster_Grouper_30298_MAP_3	LTR	SACI-7_2p	2032	311	161	84	77
Sj_Blaster_Grouper_32463_MAP_3	LTR	Novel	2647	0	314	72	253
Sj_Blaster_Grouper_27738_MAP_3	LTR	BN000804_1p	2948	410	126	70	166
Sj_Blaster_Recon_221_MAP_10	LTR	NONAUT-5_1p	3535	149	166	139	121
Sj_Blaster_Recon_16163_MAP_3	LTR	NONAUT-3_1p	4212	183	128	165	135
Sj_Blaster_Grouper_25359_MAP_3	LTR	NONAUT-5_1p	2584	121	135	141	160
Sj_Blaster_Grouper_23498_MAP_3	LTR	NONAUT-3_1p	2136	164	131	138	141
Sj_Blaster_Recon_10090_MAP_3	LTR	SACI-7_2p	1874	131	301	93	114
Sj_Blaster_Piler_49.2_MAP_14	LTR	SACI-5_1p	962	109	225	83	245
Sj_Blaster_Grouper_24715_MAP_3	LTR	BN000804_1p	2430	422	126	72	168
Sj_Blaster_Grouper_21605_MAP_3	LTR	Novel	1338	15	266	41	318
Sj_Blaster_Grouper_31746_MAP_3	LTR	Novel	1248	168	157	183	130
Sj_Blaster_Recon_3248_MAP_15	LTR	NONAUT-5_1p	5207	208	149	177	95
Sj_Blaster_Grouper_28908_MAP_3	LTR	SACI-7_2p	2068	172	344	75	86
Sj_Blaster_Grouper_26068_MAP_4	LTR	NONAUT-5_1p	2795	147	132	155	140
Sj_Blaster_Recon_452_MAP_20	LTR	BEL9_AGp	4189	189	247	61	135
Sj_Blaster_Grouper_26711_MAP_5	LTR	SACI-7_2p	1733	203	211	102	139
Sj_Blaster_Grouper_23433_MAP_20	LTR	NONAUT-5_1p	2650	160	131	158	146
Sj_Blaster_Grouper_26335_MAP_3	LTR	NONAUT-5_1p	2842	143	152	144	135
Sj_Blaster_Grouper_8607_MAP_3	LTR	Novel	611	252	299	180	97
Sj_Blaster_Recon_8685_MAP_7	LTR	Novel	1902	735	29	745	4
Sj_Blaster_Grouper_30321_MAP_3	LTR	BN000804_1p	3654	412	161	75	109
Sj_Blaster_Grouper_10201_MAP_3	LTR	Novel	666	306	439	77	94
Sj_Blaster_Grouper_29695_MAP_3	LTR	NONAUT-5_1p	3555	189	226	99	97
Sj_Blaster_Grouper_20332_MAP_4	LTR	Novel	1597	0	182	0	480

Sj_Blaster_Grouper_34334_MAP_4	LTR	GYPSY21_AGp	1398	209	177	99	181
Sj_Blaster_Grouper_32031_MAP_3	LTR	Novel	883	275	185	151	122
Sj_Blaster_Grouper_28527_MAP_3	LTR	BN000804_1p	3816	159	453	119	99
Sj_Blaster_Grouper_31754_MAP_4	LTR	GYPSY2_AGp	2698	157	337	101	127
Sj_Blaster_Grouper_26364_MAP_3	LTR	BN000804_1p	2863	461	249	91	114
Sj_Blaster_Recon_1775_MAP_20	LTR	SACI-7_2p	6271	230	202	226	143
Sj_Blaster_Grouper_24909_MAP_3	LTR	SACI-7_2p	1775	272	230	94	147
Sj_Blaster_Grouper_34340_MAP_3	LTR	SACI-7_1p	2026	283	214	116	129
Sj_Blaster_Grouper_10129_MAP_8	LTR	Novel	684	236	328	83	176
Sj_Blaster_Grouper_30184_MAP_3	LTR	NONAUT-5_1p	2168	188	382	178	132
Sj_Blaster_Grouper_28900_MAP_6	LTR	NONAUT-3_1p	2911	231	194	116	218
Sj_Blaster_Recon_3_MAP_20	LTR	NONAUT-5_1p	3736	143	267	190	116
Sj_Blaster_Recon_6262_MAP_5	LTR	Novel	1873	0	209	0	524
Sj_Blaster_Grouper_31186_MAP_3	LTR	NONAUT-3_1p	1823	153	210	104	218
Sj_Blaster_Grouper_26070_MAP_3	LTR	SACI-7_2p	1668	297	185	112	142
Sj_Blaster_Grouper_30949_MAP_3	LTR	SACI-7_2p	1215	270	341	127	101
Sj_Blaster_Grouper_35013_MAP_13	LTR	Novel	4278	6	401	115	292
Sj_Blaster_Grouper_26224_MAP_3	LTR	SACI-7_2p	2822	339	237	113	142
Sj_Blaster_Recon_7265_MAP_3	LTR	Novel	2191	0	208	0	537
Sj_Blaster_Grouper_24642_MAP_3	LTR	GYPSY2_AGp	2410	232	404	110	97
Sj_Blaster_Grouper_32012_MAP_3	LTR	NONAUT-5_1p	2043	274	237	113	100
Sj_Blaster_Grouper_29126_MAP_3	LTR	NONAUT-5_1p	2042	222	243	183	116
Sj_Blaster_Grouper_30192_MAP_3	LTR	NONAUT-3_1p	2796	208	244	206	111
Sj_Blaster_Recon_9266_MAP_5	LTR	SACI-7_2p	5487	228	353	104	122
Sj_Blaster_Recon_3553_MAP_17	LTR	Novel	3210	388	705	0	0
Sj_Blaster_Grouper_29586_MAP_5	LTR	NONAUT-5_1p	1796	197	347	118	182
Sj_Blaster_Recon_1606_MAP_20	LTR	BN000804_1p	5558	166	477	132	98
Sj_Blaster_Grouper_24166_MAP_13	LTR	GYPSY2_AGp	2061	235	295	191	202
Sj_Blaster_Recon_297_MAP_20	LTR	NONAUT-5_1p	3066	347	183	166	127
Sj_Blaster_Piler_160.190_MAP_5	LTR	Novel	828	465	213	199	94
Sj_Blaster_Recon_1204_MAP_4	LTR	Novel	1819	14	0	864	0
Sj_Blaster_Grouper_19473_MAP_9	LTR	GYPSY6-I_CB_ORF	1464	244	249	170	181
Sj_Blaster_Recon_259_MAP_20	LTR	Novel	1015	263	479	88	184
Sj_Blaster_Recon_10988_MAP_6	LTR	SACI-7_2p	2208	369	237	140	162

Sj_Blaster_Recon_5703_MAP_12	LTR	GYPSY70_AG2p	3794	394	273	182	109
Sj_Blaster_Grouper_23458_MAP_3	LTR	NONAUT-5_1p	2157	221	182	239	279
Sj_Blaster_Recon_82_MAP_20	LTR	SACI-7_1p	3400	351	216	214	183
Sj_Blaster_Grouper_31146_MAP_20	LTR	GYPSY53_AG2p	2155	486	261	118	132
Sj_Blaster_Grouper_26584_MAP_3	LTR	BEL1-I_SM_1p	2945	668	326	100	108
Sj_Blaster_Recon_91_MAP_20	LTR	GYPSY67_AGP	4600	325	252	131	213
Sj_Blaster_Grouper_25751_MAP_3	LTR	SACI-7_2p	2470	198	369	169	139
Sj_Blaster_Recon_3200_MAP_10	LTR	BN000804_1p	3925	567	299	101	135
Sj_Blaster_Grouper_21164_MAP_3	LTR	Novel	1711	79	715	15	711
Sj_Blaster_Grouper_22356_MAP_4	LTR	SACI-7_2p	1957	427	278	149	169
Sj_Blaster_Grouper_30060_MAP_5	LTR	BN000804_1p	3551	254	340	170	179
Sj_Blaster_Grouper_17826_MAP_7	LTR	SACI-7_1p	1139	283	434	132	148
Sj_Blaster_Grouper_32285_MAP_3	LTR	Novel	2542	247	0	684	0
Sj_Blaster_Recon_2380_MAP_4	LTR	SACI-7_2p	3503	360	385	164	159
Sj_Blaster_Grouper_31916_MAP_3	LTR	GYPSY2_AGp	2886	265	482	124	132
Sj_Blaster_Grouper_30214_MAP_4	LTR	Novel	5427	3	522	119	412
Sj_Blaster_Recon_674_MAP_20	LTR	BN000804_1p	5497	311	618	159	115
Sj_Blaster_Piler_268.33_MAP_20	LTR	Novel	676	213	417	253	170
Sj_Blaster_Grouper_29560_MAP_5	LTR	Gypsy9-I_AP_1p	2631	263	523	148	156
Sj_Blaster_Recon_1669_MAP_6	LTR	NONAUT-3_1p	3084	218	292	259	223
Sj_Blaster_Grouper_29536_MAP_3	LTR	NONAUT-5_1p	1301	309	269	221	159
Sj_Blaster_Grouper_26138_MAP_3	LTR	NONAUT-3_1p	1933	235	243	248	183
Sj_Blaster_Recon_399_MAP_20	LTR	Novel	1593	317	323	156	202
Sj_Blaster_Grouper_25484_MAP_6	LTR	NONAUT-5_1p	2697	399	288	158	161
Sj_Blaster_Grouper_24943_MAP_6	LTR	NONAUT-5_1p	2487	273	376	233	136
Sj_Blaster_Recon_810_MAP_6	LTR	SACI-7_2p	3666	287	563	143	156
Sj_Blaster_Grouper_27568_MAP_4	LTR	SACI-7_2p	1716	343	334	145	235
Sj_Blaster_Grouper_20184_MAP_16	LTR	SACI-7_2p	1453	432	284	160	210
Sj_Blaster_Grouper_35015_MAP_3	LTR	SACI-7_2p	2250	464	399	141	159
Sj_Blaster_Grouper_28277_MAP_6	LTR	NONAUT-3_1p	2182	315	257	246	230
Sj_Blaster_Grouper_35052_MAP_7	LTR	Novel	17771	110	0	844	109
Sj_Blaster_Grouper_31098_MAP_4	LTR	GYPSY2_AGp	4504	400	317	223	189
Sj_Blaster_Grouper_22719_MAP_3	LTR	NONAUT-5_1p	1586	341	308	251	216
Sj_Blaster_Grouper_28314_MAP_3	LTR	SACI-7_2p	1692	298	494	189	145

Sj_Blaster_Grouper_30196_MAP_3	LTR	BN000804_1p	4997	313	600	229	166
Sj_Blaster_Recon_32_MAP_20	LTR	GYPSY2_AGp	4859	404	398	140	259
Sj_Blaster_Recon_520_MAP_15	LTR	NONAUT-5_1p	2554	446	249	252	155
Sj_Blaster_Grouper_30976_MAP_5	LTR	SACI-7_2p	1400	337	384	251	223
Sj_Blaster_Grouper_26373_MAP_3	LTR	NONAUT-5_1p	2107	296	452	135	270
Sj_Blaster_Grouper_24065_MAP_7	LTR	NONAUT-5_1p	2072	359	296	212	279
Sj_Blaster_Grouper_24258_MAP_4	LTR	NONAUT-3_1p	2324	438	372	202	161
Sj_Blaster_Grouper_28850_MAP_7	LTR	GYPSM1_I_1p	3615	981	964	155	106
Sj_Blaster_Grouper_26105_MAP_4	LTR	NONAUT-5_1p	2806	337	426	162	177
Sj_Blaster_Grouper_27619_MAP_3	LTR	SACI-7_2p	2402	519	326	185	178
Sj_Blaster_Grouper_28381_MAP_8	LTR	NONAUT-5_1p	2469	328	399	282	156
Sj_Blaster_Grouper_25288_MAP_8	LTR	NONAUT-5_1p	2725	340	414	281	164
Sj_Blaster_Grouper_30832_MAP_3	LTR	BN000804_1p	4177	418	732	209	165
Sj_Blaster_Grouper_26460_MAP_3	LTR	NONAUT-3_1p	2899	484	398	209	203
Sj_Blaster_Grouper_28408_MAP_20	LTR	SACI-7_2p	2438	544	389	164	198
Sj_Blaster_Grouper_32909_MAP_5	LTR	SACI-7_2p	2679	538	324	191	212
Sj_Blaster_Grouper_29821_MAP_3	LTR	Novel	1420	467	294	373	100
Sj_Blaster_Grouper_31849_MAP_20	LTR	NONAUT-5_1p	3977	471	259	199	303
Sj_Blaster_Recon_4659_MAP_20	LTR	Novel	2764	80	969	17	995
Sj_Blaster_Grouper_28884_MAP_3	LTR	GYPSY2_AGp	2106	345	580	188	197
Sj_Blaster_Recon_473_MAP_20	LTR	BN000804_1p	2909	446	894	149	118
Sj_Blaster_Grouper_31257_MAP_4	LTR	GYPSY2_AGp	4720	452	435	282	170
Sj_Blaster_Grouper_15127_MAP_3	LTR	SACI-5_1p	955	1029	492	135	146
Sj_Blaster_Grouper_22705_MAP_4	LTR	NONAUT-5_1p	2008	410	295	240	276
Sj_Blaster_Grouper_29458_MAP_3	LTR	GYPSY54_AG2p	2333	1324	1171	74	77
Sj_Blaster_Grouper_30347_MAP_3	LTR	GYPSY48_AG2p	3398	378	624	202	157
Sj_Blaster_Grouper_31752_MAP_3	LTR	SACI-7_2p	1435	554	437	201	228
Sj_Blaster_Recon_350_MAP_20	LTR	GYPSY3_AGp	4050	333	473	292	186
Sj_Blaster_Grouper_20946_MAP_20	LTR	SACI-7_2p	1658	421	626	204	159
Sj_Blaster_Grouper_30910_MAP_5	LTR	GYPSY21_AGp	5265	469	280	221	328
Sj_Blaster_Grouper_17080_MAP_3	LTR	Novel	1137	687	674	176	145
Sj_Blaster_Grouper_27050_MAP_5	LTR	NONAUT-5_1p	3137	347	452	298	196
Sj_Blaster_Grouper_31028_MAP_3	LTR	GYPSY2_AGp	5761	444	393	296	204
Sj_Blaster_Grouper_30430_MAP_3	LTR	NONAUT-3_1p	1423	462	370	261	285

Sj_Blaster_Grouper_28427_MAP_7	LTR	NONAUT-5_1p	2645	528	352	319	159
Sj_Blaster_Grouper_22912_MAP_4	LTR	Novel	2032	326	21	846	114
Sj_Blaster_Grouper_29769_MAP_3	LTR	NONAUT-5_1p	2642	426	382	257	242
Sj_Blaster_Grouper_34601_MAP_9	LTR	Novel	2200	337	12	898	78
Sj_Blaster_Grouper_34288_MAP_4	LTR	SACI-7_2p	2142	411	590	189	172
Sj_Blaster_Grouper_25657_MAP_3	LTR	NONAUT-5_1p	2398	308	376	276	412
Sj_Blaster_Grouper_30524_MAP_3	LTR	BN000804_1p	5411	736	429	220	248
Sj_Blaster_Grouper_31125_MAP_3	LTR	GYPSY2_AGp	5315	472	438	320	202
Sj_Blaster_Grouper_31342_MAP_3	LTR	GYPSY2_AGp	3525	474	388	294	291
Sj_Blaster_Grouper_32039_MAP_13	LTR	GYPSY2_AGp	6569	491	394	361	197
Sj_Blaster_Grouper_30460_MAP_6	LTR	NONAUT-5_1p	2522	342	588	197	309
Sj_Blaster_Grouper_28068_MAP_3	LTR	BEL14_AGp	2157	468	961	144	189
Sj_Blaster_Grouper_26293_MAP_3	LTR	NONAUT-3_1p	2471	482	492	253	188
Sj_Blaster_Grouper_27856_MAP_6	LTR	NONAUT-3_1p	3442	389	347	275	356
Sj_Blaster_Grouper_31101_MAP_3	LTR	BN000804_1p	5226	443	745	254	221
Sj_Blaster_Grouper_31007_MAP_3	LTR	NONAUT-5_1p	2338	306	392	279	420
Sj_Blaster_Recon_2892_MAP_17	LTR	BEL1-I_AG-ORF1p	3359	349	839	221	172
Sj_Blaster_Grouper_27283_MAP_3	LTR	NONAUT-5_1p	3203	500	493	202	231
Sj_Blaster_Grouper_34669_MAP_3	LTR	BEL1-I_AG-ORF1p	5209	487	382	278	212
Sj_Blaster_Grouper_34592_MAP_4	LTR	BEL1-I_AG-ORF1p	5077	485	397	264	206
Sj_Blaster_Grouper_22677_MAP_3	LTR	SACI-7_2p	1989	394	613	230	191
Sj_Blaster_Grouper_27703_MAP_17	LTR	SACI-7_2p	1892	426	577	246	211
Sj_Blaster_Grouper_34172_MAP_20	LTR	Novel	2211	412	18	922	120
Sj_Blaster_Grouper_30885_MAP_20	LTR	GYPSY2_AGp	5823	489	536	188	330
Sj_Blaster_Grouper_29071_MAP_4	LTR	NONAUT-5_1p	4190	387	452	364	207
Sj_Blaster_Grouper_33716_MAP_12	LTR	NONAUT-5_1p	3617	396	370	347	263
Sj_Blaster_Grouper_30394_MAP_4	LTR	BEL10_AGp	5686	420	519	218	306
Sj_Blaster_Grouper_33899_MAP_4	LTR	SACI-7_2p	1333	556	426	385	226
Sj_Blaster_Grouper_27443_MAP_3	LTR	BN000804_1p	3118	712	519	269	358
Sj_Blaster_Grouper_29566_MAP_3	LTR	NONAUT-3_1p	2365	319	415	235	501
Sj_Blaster_Grouper_28631_MAP_3	LTR	BN000804_1p	3861	688	533	334	337
Sj_Blaster_Grouper_20113_MAP_10	LTR	Novel	2152	19	367	87	1143
Sj_Blaster_Grouper_26162_MAP_5	LTR	NONAUT-5_1p	2818	536	500	341	291
Sj_Blaster_Grouper_21000_MAP_3	LTR	SACI-5_1p	1693	964	723	324	159

Sj_Blaster_Grouper_30928_MAP_3	LTR	Novel	1522	436	752	159	342
Sj_Blaster_Grouper_29722_MAP_3	LTR	NONAUT-3_1p	2378	504	433	369	286
Sj_Blaster_Grouper_30022_MAP_4	LTR	NONAUT-5_1p	2877	486	584	278	346
Sj_Blaster_Grouper_25883_MAP_20	LTR	SACI-7_2p	2824	454	647	293	285
Sj_Blaster_Grouper_30637_MAP_3	LTR	NONAUT-5_1p	3125	294	413	423	541
Sj_Blaster_Grouper_30285_MAP_3	LTR	BN000804_1p	3508	786	491	308	359
Sj_Blaster_Grouper_30945_MAP_3	LTR	SACI-7_1p	3749	609	504	327	340
Sj_Blaster_Grouper_34294_MAP_3	LTR	GYPSY53_AG2p	3153	539	755	278	203
Sj_Blaster_Piler_512.68_MAP_3	LTR	NONAUT-5_1p	4977	622	545	249	279
Sj_Blaster_Grouper_28248_MAP_3	LTR	BN000804_1p	3639	715	541	408	329
Sj_Blaster_Grouper_34431_MAP_11	LTR	Novel	2543	489	19	1019	156
Sj_Blaster_Grouper_28689_MAP_7	LTR	NONAUT-5_1p	3756	377	418	378	482
Sj_Blaster_Grouper_28215_MAP_3	LTR	NONAUT-5_1p	3626	518	569	279	260
Sj_Blaster_Grouper_34206_MAP_3	LTR	Novel	1453	825	632	394	171
Sj_Blaster_Recon_5332_MAP_5	LTR	Novel	1405	511	6	2217	0
Sj_Blaster_Grouper_32222_MAP_4	LTR	NONAUT-5_1p	2760	546	375	415	437
Sj_Blaster_Grouper_33426_MAP_3	LTR	SACI-7_2p	1730	279	1039	133	363
Sj_Blaster_Recon_1980_MAP_20	LTR	BN000804_1p	4035	568	786	341	389
Sj_Blaster_Grouper_29761_MAP_5	LTR	NONAUT-5_1p	4849	592	485	296	429
Sj_Blaster_Grouper_29522_MAP_3	LTR	NONAUT-5_1p	2498	531	687	262	336
Sj_Blaster_Recon_301_MAP_20	LTR	BN000804_1p	6064	904	626	238	311
Sj_Blaster_Grouper_27989_MAP_3	LTR	SACI-7_2p	3492	694	501	349	396
Sj_Blaster_Grouper_32413_MAP_6	LTR	SACI-7_2p	1703	843	560	258	314
Sj_Blaster_Grouper_29655_MAP_5	LTR	GYPSM1_I_1p	4714	1345	1556	245	132
Sj_Blaster_Grouper_30839_MAP_3	LTR	SACI-7_2p	2419	542	832	304	296
Sj_Blaster_Grouper_27136_MAP_4	LTR	GYPSY2_AGp	2820	728	486	438	302
Sj_Blaster_Grouper_34150_MAP_3	LTR	SACI-7_2p	3286	497	737	384	366
Sj_Blaster_Grouper_34485_MAP_3	LTR	GYPSY2_AGp	2483	524	864	419	232
Sj_Blaster_Grouper_24148_MAP_3	LTR	NONAUT-5_1p	2301	633	428	395	453
Sj_Blaster_Grouper_21473_MAP_3	LTR	SACI-5_1p	1772	1143	631	373	221
Sj_Blaster_Grouper_27837_MAP_4	LTR	BN000804_1p	3424	595	844	391	414
Sj_Blaster_Grouper_22574_MAP_9	LTR	Novel	1958	735	521	539	157
Sj_Blaster_Grouper_31353_MAP_3	LTR	NONAUT-5_1p	1837	730	416	357	549
Sj_Blaster_Grouper_32790_MAP_20	LTR	GYPSY70_AG2p	3106	548	850	345	322

Sj_Blaster_Grouper_30423_MAP_3	LTR	NONAUT-3_1p	2517	573	476	445	407
Sj_Blaster_Grouper_34018_MAP_3	LTR	SACI-7_2p	2522	668	800	337	272
Sj_Blaster_Grouper_31926_MAP_4	LTR	NONAUT-3_1p	2301	596	446	472	457
Sj_Blaster_Grouper_32275_MAP_3	LTR	SACI-7_2p	2617	588	979	320	277
Sj_Blaster_Recon_124_MAP_20	LTR	NONAUT-3_1p	3519	548	470	503	476
Sj_Blaster_Grouper_29217_MAP_6	LTR	NONAUT-3_1p	3228	611	469	480	452
Sj_Blaster_Recon_7382_MAP_3	LTR	ATCOPIA6I_1p	587	0	93	0	3389
Sj_Blaster_Grouper_23367_MAP_20	LTR	SACI-7_2p	2245	668	942	386	227
Sj_Blaster_Grouper_34662_MAP_5	LTR	Novel	3083	598	29	1430	140
Sj_Blaster_Grouper_33447_MAP_6	LTR	SACI-7_2p	4399	1008	671	312	332
Sj_Blaster_Grouper_27839_MAP_4	LTR	SACI-7_2p	2801	600	872	444	324
Sj_Blaster_Grouper_31974_MAP_7	LTR	GYPSY2_AGp	2964	1023	627	286	364
Sj_Blaster_Grouper_29831_MAP_4	LTR	SACI-7_2p	3139	949	715	309	362
Sj_Blaster_Grouper_29795_MAP_3	LTR	NONAUT-5_1p	4881	737	700	421	288
Sj_Blaster_Grouper_29926_MAP_3	LTR	SACI-7_2p	2295	734	863	400	331
Sj_Blaster_Grouper_27785_MAP_7	LTR	Novel	3110	535	24	1483	178
Sj_Blaster_Recon_1808_MAP_11	LTR	GYPSM1_I_1p	4784	836	1090	333	275
Sj_Blaster_Grouper_30176_MAP_3	LTR	NONAUT-5_1p	3661	831	619	532	359
Sj_Blaster_Grouper_17275_MAP_3	LTR	Novel	1136	1446	1118	218	291
Sj_Blaster_Grouper_29801_MAP_3	LTR	NONAUT-5_1p	4890	529	605	505	582
Sj_Blaster_Grouper_26596_MAP_3	LTR	NONAUT-5_1p	2383	491	778	608	467
Sj_Blaster_Grouper_28135_MAP_4	LTR	Novel	1745	912	1181	221	440
Sj_Blaster_Grouper_29511_MAP_11	LTR	NONAUT-5_1p	4604	644	533	582	516
Sj_Blaster_Grouper_28358_MAP_3	LTR	BN000804_1p	3161	760	1366	267	302
Sj_Blaster_Grouper_29591_MAP_3	LTR	SACI-7_2p	3492	608	959	470	400
Sj_Blaster_Grouper_34921_MAP_3	LTR	SACI-5_1p	2549	968	1132	398	405
Sj_Blaster_Grouper_34331_MAP_3	LTR	SACI-7_2p	2408	737	1001	384	326
Sj_Blaster_Grouper_33239_MAP_3	LTR	NONAUT-3_1p	3114	699	576	568	491
Sj_Blaster_Grouper_22854_MAP_3	LTR	Novel	1463	1490	1239	248	699
Sj_Blaster_Grouper_31978_MAP_3	LTR	BN000804_1p	2791	805	1556	226	276
Sj_Blaster_Grouper_28285_MAP_5	LTR	SACI-5_1p	2359	1218	801	572	292
Sj_Blaster_Grouper_25152_MAP_9	LTR	NONAUT-3_1p	2667	575	775	502	556
Sj_Blaster_Grouper_32107_MAP_3	LTR	SACI-7_2p	4146	1103	654	388	405
Sj_Blaster_Grouper_29925_MAP_3	LTR	SACI-7_2p	3274	1153	683	349	417

Sj_Blaster_Recon_366_MAP_20	LTR	SACI-5_1p	2730	1414	1012	505	251
Sj_Blaster_Grouper_25758_MAP_3	LTR	NONAUT-3_1p	2701	543	815	575	536
Sj_Blaster_Grouper_30754_MAP_3	LTR	SACI-7_2p	4197	684	1189	407	390
Sj_Blaster_Grouper_29061_MAP_3	LTR	NONAUT-5_1p	4093	645	907	460	525
Sj_Blaster_Grouper_32253_MAP_3	LTR	Novel	1078	1551	1171	613	330
Sj_Blaster_Grouper_27662_MAP_20	LTR	GYPSY48_AG2p	3502	1140	762	363	423
Sj_Blaster_Grouper_32589_MAP_3	LTR	SACI-7_2p	3642	834	1067	442	336
Sj_Blaster_Grouper_29950_MAP_3	LTR	SACI-7_2p	3309	1088	860	332	438
Sj_Blaster_Grouper_31661_MAP_3	LTR	GYPSY2_AGp	4133	731	1075	479	424
Sj_Blaster_Grouper_31229_MAP_3	LTR	SACI-7_2p	3517	791	1125	457	395
Sj_Blaster_Grouper_27865_MAP_3	LTR	Novel	1248	1458	1160	697	344
Sj_Blaster_Grouper_32033_MAP_3	LTR	GYPSY2_AGp	5083	1164	728	412	482
Sj_Blaster_Grouper_31141_MAP_3	LTR	NONAUT-3_1p	3030	853	647	613	558
Sj_Blaster_Grouper_28074_MAP_3	LTR	SACI-7_2p	3329	1160	702	470	463
Sj_Blaster_Grouper_27434_MAP_3	LTR	NONAUT-3_1p	3016	635	839	744	617
Sj_Blaster_Grouper_26856_MAP_8	LTR	NONAUT-3_1p	2875	873	647	592	669
Sj_Blaster_Grouper_31594_MAP_6	LTR	Novel	3641	836	1122	245	708
Sj_Blaster_Grouper_34990_MAP_3	LTR	SACI-7_2p	1613	883	1267	545	335
Sj_Blaster_Grouper_27957_MAP_3	LTR	NONAUT-3_1p	3175	874	678	688	600
Sj_Blaster_Grouper_23950_MAP_20	LTR	Gypsy1-I_AP_1p	3934	914	1219	482	396
Sj_Blaster_Grouper_28899_MAP_3	LTR	SACI-7_2p	4056	813	1101	630	531
Sj_Blaster_Grouper_29969_MAP_7	LTR	NONAUT-5_1p	5111	1089	1055	456	525
Sj_Blaster_Grouper_31973_MAP_7	LTR	SACI-5_1p	3407	1320	918	754	335
Sj_Blaster_Grouper_34257_MAP_3	LTR	Novel	3247	935	1202	309	665
Sj_Blaster_Grouper_23550_MAP_20	LTR	SACI-5_1p	2173	1767	1553	516	326
Sj_Blaster_Grouper_30615_MAP_20	LTR	SACI-7_2p	3269	1396	941	399	435
Sj_Blaster_Recon_1142_MAP_20	LTR	GYPSM1_I_1p	4796	1512	1639	390	298
Sj_Blaster_Recon_1143_MAP_12	LTR	GYPSM1_I_1p	4451	1523	1219	372	486
Sj_Blaster_Grouper_29674_MAP_3	LTR	Novel	1497	1239	1964	348	567
Sj_Blaster_Grouper_21317_MAP_3	LTR	Novel	1601	2057	1453	368	388
Sj_Blaster_Grouper_28436_MAP_3	LTR	NONAUT-3_1p	3484	945	728	729	648
Sj_Blaster_Grouper_31660_MAP_3	LTR	GYPSY2_AGp	4430	910	1316	557	463
Sj_Blaster_Grouper_28752_MAP_4	LTR	NONAUT-5_1p	3974	842	1022	664	600
Sj_Blaster_Grouper_30561_MAP_3	LTR	Novel	1943	1649	1603	966	381

Sj_Blaster_Grouper_22804_MAP_20	LTR	NONAUT-3_1p	3329	993	736	668	704
Sj_Blaster_Recon_1850_MAP_20	LTR	GYPSM1_I_1p	4779	1417	1574	471	364
Sj_Blaster_Grouper_28639_MAP_3	LTR	SACI-7_2p	3751	1241	831	619	632
Sj_Blaster_Grouper_34059_MAP_6	LTR	Novel	3361	1402	1010	741	316
Sj_Blaster_Grouper_34014_MAP_3	LTR	NONAUT-3_1p	3571	993	728	720	715
Sj_Blaster_Grouper_30068_MAP_19	LTR	SACI-7_2p	4009	976	1477	569	422
Sj_Blaster_Grouper_30665_MAP_3	LTR	NONAUT-5_1p	5818	1244	1170	490	588
Sj_Blaster_Grouper_35010_MAP_5	LTR	NONAUT-3_1p	3510	797	1042	723	713
Sj_Blaster_Grouper_32252_MAP_3	LTR	SACI-7_2p	3473	1414	1070	543	481
Sj_Blaster_Grouper_28468_MAP_3	LTR	Novel	1834	1586	2139	387	598
Sj_Blaster_Grouper_33460_MAP_3	LTR	GYPSY2_AGp	3204	1166	1316	653	643
Sj_Blaster_Grouper_25762_MAP_3	LTR	Novel	2462	1550	2203	425	583
Sj_Blaster_Grouper_30138_MAP_3	LTR	Novel	1743	1649	1937	363	879
Sj_Blaster_Grouper_30898_MAP_3	LTR	BN000804_1p	6161	1211	2283	411	439
Sj_Blaster_Grouper_31624_MAP_3	LTR	SACI-7_2p	4346	1597	1065	483	635
Sj_Blaster_Piler_541.51_MAP_5	LTR	SACI-7_2p	5292	1656	1079	475	586
Sj_Blaster_Grouper_34311_MAP_3	LTR	SACI-7_2p	3625	1501	1293	573	686
Sj_Blaster_Grouper_29767_MAP_3	LTR	SACI-7_2p	4341	989	1526	760	703
Sj_Blaster_Grouper_31914_MAP_3	LTR	Novel	1900	1815	2390	389	619
Sj_Blaster_Grouper_34929_MAP_9	LTR	Novel	4096	1001	1419	352	1012
Sj_Blaster_Grouper_33508_MAP_20	LTR	Gypsy1-I_AP_1p	4879	1142	1703	639	497
Sj_Blaster_Grouper_28890_MAP_3	LTR	SACI-7_2p	4052	1591	1047	731	791
Sj_Blaster_Grouper_35021_MAP_3	LTR	NONAUT-3_1p	4413	1319	912	796	832
Sj_Blaster_Grouper_30326_MAP_20	LTR	GYPSY48_AG2p	5433	1128	1777	672	528
Sj_Blaster_Grouper_34415_MAP_3	LTR	SACI-7_2p	5037	1163	1758	711	515
Sj_Blaster_Piler_586.51_MAP_10	LTR	SACI-7_2p	5354	1138	1844	665	543
Sj_Blaster_Grouper_34638_MAP_5	LTR	SACI-7_2p	3921	1317	1789	690	504
Sj_Blaster_Grouper_31388_MAP_4	LTR	NONAUT-3_1p	4177	1343	978	931	1014
Sj_Blaster_Grouper_32887_MAP_4	LTR	Novel	2037	2586	2389	714	528
Sj_Blaster_Grouper_33708_MAP_3	LTR	Novel	1923	2505	2052	912	506
Sj_Blaster_Grouper_24860_MAP_3	LTR	Novel	2143	2450	2442	939	473
Sj_Blaster_Grouper_33199_MAP_15	LTR	SACI-5_1p	4476	1627	2120	507	915
Sj_Blaster_Grouper_26978_MAP_3	LTR	Novel	1959	2375	2221	1009	694
Sj_Blaster_Grouper_34332_MAP_4	LTR	GYPSY2_AGp	5120	1353	2012	689	647

Sj_Blaster_Grouper_32293_MAP_20	LTR	Novel	2726	1902	1215	960	564
Sj_Blaster_Grouper_31972_MAP_3	LTR	SACI-5_1p	4112	2362	1679	888	500
Sj_Blaster_Grouper_35100_MAP_3	LTR	Novel	2096	2854	1801	891	567
Sj_Blaster_Grouper_34722_MAP_3	LTR	SACI-7_2p	4260	1326	2073	835	603
Sj_Blaster_Grouper_33857_MAP_4	LTR	Novel	2244	2906	2490	599	563
Sj_Blaster_Grouper_34732_MAP_3	LTR	Novel	2219	2903	2341	758	551
Sj_Blaster_Grouper_30708_MAP_3	LTR	Novel	2826	2848	2333	556	1235
Sj_Blaster_Grouper_33477_MAP_4	LTR	Novel	2358	3329	2901	655	654
Sj_Blaster_Grouper_34540_MAP_3	LTR	Novel	2911	3181	2770	1019	652
Sj_Blaster_Grouper_22218_MAP_20	LTR	Novel	2066	3722	3029	743	757
Sj_Blaster_Recon_2785_MAP_19	LTR	Novel	7436	10	659	63	7503
Sj_Blaster_Grouper_34753_MAP_3	LTR	Novel	2678	3660	2791	1095	762
Sj_Blaster_Grouper_27873_MAP_3	LTR	Novel	2277	3449	3058	1946	702
Sj_Blaster_Grouper_27795_MAP_3	LTR	Novel	2317	3250	3544	669	1765
Sj_Blaster_Recon_7298_MAP_7	LTR	Novel	2729	3259	4246	750	1583
Sj_Blaster_Grouper_35124_MAP_3	LTR	Novel	3377	4248	3678	1497	802
Sj_Blaster_Grouper_31940_MAP_6	LTR	Novel	2960	3416	3875	737	2196
Sj_Blaster_Grouper_31427_MAP_3	LTR	Novel	3128	3919	4485	829	1561
Sj_Blaster_Grouper_26306_MAP_3	LTR	Novel	2865	3920	4722	915	1663
Sj_Blaster_Grouper_27042_MAP_3	LTR	Novel	2670	4085	4619	918	2572
Sj_Blaster_Grouper_28197_MAP_5	LTR	Novel	3519	4494	5094	851	2351
Sj_Blaster_Grouper_29327_MAP_3	LTR	Novel	3279	4779	5335	1037	2735
