# Supplementary material for: Identification and characterization of microRNAs and endogenous siRNAs in Schistosoma japonicum
Source: BMC Genomics. 2010 Jan 21;11:55. doi: 10.1186/1471-2164-11-55 (PMC2820009; doi:10.1186/1471-2164-11-55)
Supplement: Additional file 4 — SiRNAs derived from SINE. This file contains the information of the identified transposon-SINE in the S. japonicum genome and the derived siRNAs. [file 1471-2164-11-55-S4.PDF]

siRNAs derived from SINE

Name	Type	Annotation	TE Length	siRNAs (Adult)		siRNAs (schistosomula)	
				# Sense siRNAs	# AntiSense siRNAs	# Sense siRNAs	# l AntiSense siRNAs
Sj_Blaster_Recon_3571_MAP_3	SINE	Novel	399	0	1	0	0
Sj_Blaster_Recon_11619_MAP_3	SINE	Novel	1164	3	3	0	2
Sj_Blaster_Recon_17268_MAP_3	SINE	Novel	494	3	0	1	15
Sj_Blaster_Grouper_3308_MAP_3	SINE	Novel	452	8	3	19	6
Sj_Blaster_Recon_13919_MAP_3	SINE	Novel	488	12	10	12	16
Sj_Blaster_Recon_2241_MAP_3	SINE	Novel	879	13	15	9	8
Sj_Blaster_Grouper_3944_MAP_20	SINE	Novel	476	25	16	14	35
Sj_Blaster_Piler_574.198_MAP_4	SINE	T2	1884	20	17	33	40
Sj_Blaster_Recon_15535_MAP_3	SINE	Novel	389	102	16	24	25
Sj_Blaster_Grouper_3189_MAP_3	SINE	Novel	448	42	37	46	17
Sj_Blaster_Grouper_4135_MAP_5	SINE	Novel	479	91	52	19	18
Sj_Blaster_Recon_5710_MAP_3	SINE	T2	1011	44	61	32	31
Sj_Blaster_Grouper_20954_MAP_3	SINE	T2	1685	54	160	59	29
Sj_Blaster_Recon_6115_MAP_3	SINE	T2	1446	45	124	27	12
Sj_Blaster_Recon_16484_MAP_3	SINE	Novel	419	111	72	21	33
Sj_Blaster_Recon_3978_MAP_3	SINE	T2	886	165	30	54	72
Sj_Blaster_Grouper_1614_MAP_3	SINE	Novel	399	154	57	38	57
Sj_Blaster_Recon_1942_MAP_3	SINE	T2	1171	62	164	37	40
Sj_Blaster_Recon_17578_MAP_4	SINE	T2	364	152	46	65	40
Sj_Blaster_Grouper_15059_MAP_4	SINE	T2	951	68	164	41	30
Sj_Blaster_Recon_428_MAP_4	SINE	T2	2353	98	156	74	74
Sj_Blaster_Recon_5379_MAP_3	SINE	T2	679	89	192	61	53
Sj_Blaster_Grouper_3352_MAP_3	SINE	T2	454	97	124	137	76
Sj_Blaster_Grouper_3416_MAP_4	SINE	Novel	456	190	259	57	165
Sj_Blaster_Grouper_2100_MAP_4	SINE	Novel	415	239	148	77	299
Sj_Blaster_Grouper_29419_MAP_3	SINE	T2	815	378	448	163	290
Sj_Blaster_Grouper_10911_MAP_3	SINE	T2	698	295	651	360	189
Sj_Blaster_Grouper_11_MAP_20	SINE	T2	1229	493	872	490	319
Sj_Blaster_Grouper_18301_MAP_4	SINE	T2	1150	531	952	587	347
Sj_Blaster_Grouper_23062_MAP_3	SINE	T2	1779	586	1197	517	482
