# Supplementary material for: Identification and characterization of microRNAs and endogenous siRNAs in Schistosoma japonicum
Source: BMC Genomics. 2010 Jan 21;11:55. doi: 10.1186/1471-2164-11-55 (PMC2820009; doi:10.1186/1471-2164-11-55)
Supplement: Additional file 5 — SiRNAs derived from TIR. This file contains the information of the identified transposon-TIR in the S. japonicum genome and the derived siRNAs. [file 1471-2164-11-55-S5.PDF]

siRNAs derived from TIR

Name	Type	Annotation	TE Length	siRNAs (Adult)		siRNAs (schistosomula)	
				# o Sense siRNAs	# AntiSense siRNAs	# Sense siRNAs	# AntiSense siRNAs
Sj_Blaster_Grouper_8917_MAP_4	TIR	Novel	622	1	0	0	0
Sj_Blaster_Grouper_10605_MAP_3	TIR	Novel	687	1	0	0	0
Sj_Blaster_Recon_5836_MAP_3	TIR	Novel	999	0	0	1	0
Sj_Blaster_Recon_4847_MAP_3	TIR	MERLIN2_SM_1p	1986	1	0	0	0
Sj_Blaster_Recon_9722_MAP_4	TIR	MERLIN1_SM_1p	1722	0	2	0	0
Sj_Blaster_Grouper_22580_MAP_3	TIR	Novel	925	0	2	0	0
Sj_Blaster_Recon_11319_MAP_5	TIR	MERLIN2_SM_1p	2784	1	0	0	2
Sj_Blaster_Recon_3540_MAP_10	TIR	MERLIN1_SM_1p	791	3	0	0	1
Sj_Blaster_Grouper_19704_MAP_3	TIR	Novel	1432	2	0	0	4
Sj_Blaster_Grouper_28519_MAP_4	TIR	Novel	3115	3	4	0	1
Sj_Blaster_Recon_4551_MAP_12	TIR	MERLIN1_SM_1p	955	1	7	0	2
Sj_Blaster_Recon_16167_MAP_5	TIR	MERLIN2_SM_1p	1447	0	2	1	5
Sj_Blaster_Recon_2986_MAP_4	TIR	SmTRC1_1p	1524	0	0	0	14
Sj_Blaster_Recon_4500_MAP_4	TIR	MERLIN2_SM_1p	2893	15	4	1	3
Sj_Blaster_Grouper_17561_MAP_7	TIR	MERLIN2_SM_1p	1193	8	5	12	2
Sj_Blaster_Recon_1984_MAP_6	TIR	MERLIN2_SM_1p	5023	3	3	12	6
Sj_Blaster_Grouper_7740_MAP_7	TIR	Novel	610	5	2	12	11
Sj_Blaster_Recon_8695_MAP_4	TIR	Novel	1897	11	7	9	8
Sj_Blaster_Grouper_30291_MAP_3	TIR	Novel	1106	0	13	0	20
Sj_Blaster_Recon_108_MAP_13	TIR	Novel	9036	23	10	10	8
Sj_Blaster_Grouper_25721_MAP_3	TIR	Novel	2589	6	36	2	24
Sj_Blaster_Grouper_27665_MAP_3	TIR	Novel	2329	22	29	7	28
Sj_Blaster_Grouper_23930_MAP_3	TIR	Novel	857	29	57	9	35
Sj_Blaster_Grouper_21064_MAP_6	TIR	Novel	1037	7	98	1	66
Sj_Blaster_Grouper_28236_MAP_3	TIR	Novel	890	0	114	0	64
Sj_Blaster_Grouper_8234_MAP_3	TIR	Novel	599	30	5	134	2
Sj_Blaster_Grouper_31627_MAP_3	TIR	Novel	5389	21	107	27	91
Sj_Blaster_Grouper_24060_MAP_3	TIR	Transib-10_HM	2260	18	4	208	84
Sj_Blaster_Grouper_30993_MAP_3	TIR	Novel	5803	0	34	0	282
Sj_Blaster_Grouper_14226_MAP_3	TIR	SmTRC1_1p	886	107	99	53	50
Sj_Blaster_Piler_377.234_MAP_3	TIR	Chapaev-8_HM	537	92	124	51	126

Sj_Blaster_Piler_505.16_MAP_4	TIR	Novel	553	72	154	98	66
Sj_Blaster_Grouper_16258_MAP_3	TIR	Novel	767	52	280	25	162
Sj_Blaster_Grouper_11654_MAP_6	TIR	Novel	733	33	7	645	4
Sj_Blaster_Piler_265.46_MAP_9	TIR	Novel	557	189	236	129	197
Sj_Blaster_Grouper_31239_MAP_3	TIR	Novel	865	51	382	32	210
Sj_Blaster_Grouper_31265_MAP_6	TIR	Novel	899	20	469	28	248
Sj_Blaster_Grouper_21275_MAP_3	TIR	SmTRC1_1p	1263	332	458	103	73
Sj_Blaster_Grouper_10968_MAP_9	TIR	SmTRC1_1p	703	364	444	105	96
Sj_Blaster_Piler_301.285_MAP_4	TIR	Transib-2_HM	1477	324	16	673	102
Sj_Blaster_Recon_7337_MAP_14	TIR	SmTRC1_1p	3026	716	454	120	158
Sj_Blaster_Grouper_31903_MAP_20	TIR	Novel	5446	0	503	12	5562
Sj_Blaster_Grouper_23601_MAP_3	TIR	EnSpm-2_HM	2126	3912	2882	726	732
Sj_Blaster_Grouper_28274_MAP_4	TIR	Novel	3661	218	2007	91	95845
