# Supplementary material for: Identification and characterization of microRNAs and endogenous siRNAs in Schistosoma japonicum
Source: BMC Genomics. 2010 Jan 21;11:55. doi: 10.1186/1471-2164-11-55 (PMC2820009; doi:10.1186/1471-2164-11-55)
Supplement: Additional file 6 — SiRNAs derived from MITE. This file contains the information of the identified transposon-MITE in the S. japonicum genome and the derived siRNAs. [file 1471-2164-11-55-S6.PDF]

siRNAs derived from MITE

Name	Type	Annotation	TE Length	siRNAs (Adult)		siRNAs (schistosomula)	
				# Sense siRNAs	# AntiSense siRNAs	# Sense siRNAs	# AntiSense siRNAs
Sj_Blaster_Piler_269.322_MAP_8	MITE	Novel	417	0	0	0	1
Sj_Blaster_Grouper_4165_MAP_4	MITE	Novel	476	1	0	1	1
Sj_Blaster_Grouper_4899_MAP_10	MITE	Novel	498	3	4	2	8
Sj_Blaster_Grouper_223_MAP_3	MITE	Novel	347	0	25	0	2
Sj_Blaster_Piler_783.81_MAP_4	MITE	Novel	465	3	9	9	5
Sj_Blaster_Grouper_4617_MAP_3	MITE	Novel	489	5	29	9	21
Sj_Blaster_Grouper_2447_MAP_6	MITE	Novel	438	17	15	22	8
Sj_Blaster_Grouper_3496_MAP_3	MITE	Novel	457	17	29	14	17
Sj_Blaster_Grouper_4691_MAP_11	MITE	Novel	496	41	46	30	30
Sj_Blaster_Grouper_2767_MAP_6	MITE	Novel	436	179	120	35	63
Sj_Blaster_Grouper_1452_MAP_3	MITE	Novel	394	117	197	51	35
Sj_Blaster_Grouper_4406_MAP_4	MITE	Novel	485	59	132	47	138
Sj_Blaster_Grouper_2287_MAP_4	MITE	Novel	421	252	132	111	53
Sj_Blaster_Grouper_2397_MAP_3	MITE	Novel	443	729	628	330	148
Sj_Blaster_Grouper_1934_MAP_4	MITE	Novel	410	2	81	0	20700
