# Supplementary material for: Identification and characterization of microRNAs and endogenous siRNAs in Schistosoma japonicum
Source: BMC Genomics. 2010 Jan 21;11:55. doi: 10.1186/1471-2164-11-55 (PMC2820009; doi:10.1186/1471-2164-11-55)
Supplement: Additional file 7 — Data Summary of siRNAs and miRNAs. This file contains the summary data of siRNA and miRNA, from stage-associated differences in length distribution to the number of transcripts. [file 1471-2164-11-55-S7.PDF]

Statistical data for siRNAs and miRNAs in *S. japonicum*

Tab1 TE-derived small RNAs

Class	Unique		Mapped by Solexa Reads		Corresponding Reads		Adu Reads		Mir Reads	
	#	%	#	%	#	%[b]	#	%[b]	#	%[b]
TEs Unique	6307	100.0	4759	100.0	203702	100.0	120582	100.0	137439	100.0
LTR	954	15.1	833	17.5	86338	42.4	60184	49.9	47814	34.8
LINE	1299	20.6	1239	26.0	53375	26.2	36777	30.5	29576	21.5
SINE	30	0.5	30	0.6	3256	1.6	2251	1.9	1893	1.4
TIR	53	0.8	44	0.9	24608	12.1	10903	9.0	21318	15.5
MITE	16	0.3	15	0.3	5661	2.8	2554	2.1	4686	3.4
SSR	83	1.3	63	1.3	2472	1.2	2472			
Confused	968	15.3	880	18.5	107597	52.8	107597			
NoCat	2904	46.0	1655	34.8	96495	47.4	96495			

[a] S.japonicuma TEs were annoted by REPET(http://urgi.versailles.inra.fr/development/repet/)

[b] Percentages total more than 100,because some siRNAs map to more than one TE

Tab2 Distribution of Sequence Length (Total Merged)

Length	Total				Perfect-matched				miRcheck-passed				TE-derived				NAT-derived			
	Unique		Total		Unique		Total		Unique		Total		Unique		Total		Unique		Total	
	#	%	#	%	#	%	#	%	#	%	#	%	#	%	#	%	#	%	#	%
18	47415	2.3	181345	1.9	20570	5	132881	2.6	1034	5.7	27044	3.4	12229	6	89655	3.9	201	7.7	711	3.6
19	105373	5	384762	4	45469	11	257644	5.1	2574	14.3	50345	6.3	25611	12.6	159346	6.9	503	19.3	2302	11.7
20	313172	14.9	1453208	15.1	138599	33.4	959402	19.1	7714	42.8	55285	6.9	71004	34.9	575979	25	1191	45.7	13823	70.3
21	307955	14.6	1484290	15.4	70405	17	866573	17.2	3477	19.3	110523	13.7	29518	14.5	361869	15.7	428	16.4	1999	10.2
22	347957	16.5	1984754	20.6	42276	10.2	959117	19.1	1814	10.1	207966	25.9	15853	7.8	210632	9.1	158	6.1	613	3.1
23	353936	16.8	2157249	22.4	26426	6.4	1070639	21.3	799	4.4	330683	41.1	10292	5.1	213933	9.3	45	1.7	118	0.6
24	246671	11.7	813745	8.5	16931	4.1	214663	4.3	277	1.5	18804	2.3	7759	3.8	171991	7.5	23	0.9	27	0.1
25	148226	7	408691	4.2	13642	3.3	157783	3.1	134	0.7	2755	0.3	6763	3.3	141036	6.1	18	0.7	24	0.1
26	82151	3.9	259612	2.7	11651	2.8	132675	2.6	75	0.4	380	0	6215	3.1	121300	5.3	16	0.6	21	0.1
27	52182	2.5	198112	2.1	9895	2.4	116551	2.3	60	0.3	85	0	5567	2.7	107213	4.7	7	0.3	8	0
28	39456	1.9	141605	1.5	8055	1.9	79120	1.6	38	0.2	50	0	4854	2.4	71740	3.1	10	0.4	10	0.1
29	29034	1.4	82003	0.9	5568	1.3	45046	0.9	24	0.1	28	0	3744	1.8	40851	1.8	5	0.2	6	0
30	19433	0.9	51036	0.5	3541	0.9	28827	0.6	9	0	16	0	2591	1.3	26630	1.2	1	0	1	0
31	10310	0.5	21897	0.2	2198	0.5	12160	0.2	1	0	2	0	1702	0.8	11090	0.5	0	0.0	0	0.0
In all	2103271	99.9	9622309	100	415226	100.2	5033081	100.0	18030	99.8	803966	99.9	203702	100.1	2303265	100.1	2606	100	19663	99.9

Tab3 Distribution of Sequence Length (Adult)

Length	Total				Perfect-matched				miRcheck-passed				TE-derived				NAT-derived			
	Unique		Total		Unique		Total		Unique		Total		Unique		Total		Unique		Total	
	#	%	#	%	#	%	#	%	#	%	#	%	#	%	#	%	#	%	#	%
18	22839	1.9	73104	1.4	10062	4.4	49254	1.9	705	5.7	26411	5.3	6196	5.1	14767	1.8	174	8	659	4.6
19	67895	5.7	236352	4.4	29660	12.9	141763	5.6	1987	16	48274	9.7	17405	14.4	58382	7.2	448	20.6	2036	14.1
20	195824	16.4	927348	17.3	88126	38.3	547413	21.6	5210	41.9	40764	8.2	47725	39.6	297361	36.8	990	45.5	9585	66.4
21	199258	16.7	962741	18	44359	19.3	555687	21.9	2502	20.1	80796	16.3	19322	16	199819	24.7	352	16.2	1513	10.5
22	213046	17.8	1174719	22	23398	10.2	530114	20.9	1227	9.9	158028	31.9	9203	7.6	54971	6.8	122	5.6	469	3.3
23	207499	17.4	1189873	22.2	12329	5.4	530843	20.9	509	4.1	129647	26.1	5270	4.4	41402	5.1	36	1.7	99	0.7
24	133515	11.2	389112	7.3	6087	2.6	55919	2.2	163	1.3	11062	2.2	3553	2.9	33237	4.1	14	0.6	16	0.1
25	69213	5.8	147738	2.8	4322	1.9	24979	1	65	0.5	834	0.2	3008	2.5	19260	2.4	10	0.5	16	0.1
26	34586	2.9	79506	1.5	3688	1.6	25840	1	31	0.2	108	0	2713	2.2	21905	2.7	13	0.6	17	0.1
27	20543	1.7	70168	1.3	3252	1.4	34895	1.4	22	0.2	30	0	2465	2	30534	3.8	7	0.3	7	0
28	15415	1.3	57232	1.1	2730	1.2	23809	0.9	15	0.1	21	0	2063	1.7	20396	2.5	7	0.3	7	0
29	9063	0.8	25005	0.5	1529	0.7	10038	0.4	7	0.1	7	0	1210	1	8188	1	3	0.1	3	0
30	5129	0.4	16217	0.3	553	0.2	8221	0.3	0	0	0	0	449	0.4	7450	0.9	0	0	0	0
31	0	0.0	0	0.0	0	0	0	0	0	0	0	0	0	0	0	0	0	0	0	0
In all	1193825	100	5349115	100.1	230095	100.1	2538775	100.0	12443	100.1	495982	99.9	120582	99.8	807672	99.8	2176	100	14427	99.9

Tab4 Distribution of Sequence Length (Schistosomulum)

Length	Total				Perfect-matched				miRcheck-passed				TE-derived				NAT-derived			
	Unique		Total		Unique		Total		Unique		Total		Unique		Total		Unique		Total	
	#	%	#	%	#	%	#	%	#	%	#	%	#	%	#	%	#	%	#	%
18	26529	2.3	108241	2.5	12078	4.5	83627	3.4	378	4.1	633	0.2	7291	5.3	74888	5	44	3.5	52	1
19	44616	3.9	148410	3.5	21410	7.9	115881	4.6	861	9.3	2071	0.7	12221	8.9	100964	6.8	128	10.3	266	5.1
20	163390	14.4	525860	12.3	87758	32.4	411989	16.5	4437	48.1	14521	4.7	46244	33.6	278618	18.6	742	59.8	4238	80.9
21	146553	12.9	521549	12.2	41512	15.3	310886	12.5	1770	19.2	29727	9.7	18166	13.2	162050	10.8	211	17	486	9.3
22	176315	15.5	810035	19	27411	10.1	429003	17.2	951	10.3	49938	16.2	11013	8	155661	10.4	71	5.7	144	2.8
23	190431	16.8	967376	22.6	19212	7.1	539796	21.6	449	4.9	201036	65.3	8062	5.9	172531	11.5	16	1.3	19	0.4
24	138654	12.2	424633	9.9	13844	5.1	158744	6.4	160	1.7	7742	2.5	6625	4.8	138754	9.3	10	0.8	11	0.2
25	90008	7.9	260953	6.1	11710	4.3	132804	5.3	82	0.9	1921	0.6	5947	4.3	121776	8.1	8	0.6	8	0.2
26	52157	4.6	180106	4.2	10008	3.7	106835	4.3	52	0.6	272	0.1	5438	4	99395	6.6	4	0.3	4	0.1
27	34483	3	127944	3	8478	3.1	81656	3.3	40	0.4	55	0	4840	3.5	76679	5.1	1	0.1	1	0
28	26153	2.3	84373	2	6757	2.5	55311	2.2	24	0.3	29	0	4143	3	51344	3.4	3	0.2	3	0.1
29	21202	1.9	56998	1.3	4875	1.8	35008	1.4	17	0.2	21	0	3322	2.4	32663	2.2	2	0.2	3	0.1
30	14840	1.3	34819	0.8	3298	1.2	20606	0.8	9	0.1	16	0	2425	1.8	19180	1.3	1	0.1	1	0
31	10310	0.9	21897	0.5	2198	0.8	12160	0.5	1	0	2	0	1702	1.2	11090	0.7	0	0.0	0	0.0
In all	1135641	99.9	4273194	99.9	270549	99.8	2494306	100.0	9231	100.1	307984	100	137439	99.9	1495593	99.8	1241	99.9	5236	100.2

Tab5 TE

Class	#	Total Length	% of TEs	% of Genome
LTR	11375	16590913	31.812172	4.2
LINE	9758	15023638	28.807008	3.8
SINE	92	52138	0.0999718	0.0
TIR	106	152704	0.2928016	0.0
MITE	19	8322	0.015957	0.0
SSR	268	220921	0.423604	0.1
Confus	4593	6479593	12.424267	1.6
NoCat	13533	13624491	26.124219	3.4
Total TEs	39744	52152720	100	13.1
