# Supplementary material for: Identification and characterization of microRNAs and endogenous siRNAs in Schistosoma japonicum
Source: BMC Genomics. 2010 Jan 21;11:55. doi: 10.1186/1471-2164-11-55 (PMC2820009; doi:10.1186/1471-2164-11-55)
Supplement: Additional file 8 — Hairpin prediction of common miRNAs in S. japonicum. This file contains the predicted hairpin structures of common miRNAs. [file 1471-2164-11-55-S8.PDF]

Hairpin prediction of common miRNAs in *S. japonicum*

```
>sja-miR-71   CCON0000096885.1 360273 360432 +
UUGCUGUGAAAGACUUGAGUAGUGAGACGCUUAUAGCGUUGUUGUCUGCGGUUCGGCGCCUCAUACUAAGUCUUUCCCGGCUC

UU|   U         G    G    A    UAUA-   UUG
  GCUG GAAAGACUU AGUA UGAG CGCU     GCG   U
  CGGC CUUUCUGAA UCAU ACUC GCGG     CGU   U
CU^   C         -    -    C    CUUGG   CUG
```

```
>sja-miR-7   CCON0000097602.1 12747 12871 +
AUCAUGGAAGACUGGUGAUAUGUUGUUGAUUGAUGAAAGUUUUAAGUAUUUCAUAAUUUAAAGCUUCAUCAAAACAAUGAUAUUACGAACAAC
UUAUCGCAUUCUUCCAUGUA

AU         CUG      U      .-GA        A         AUU
  CAUGGAAGA   GUGAUA GUUGUU    UUGAUGAA GUUUUAAGU   U
  GUACCUUCU   CGCUAU CAACAA    AACUACUU CGAAAUUUA   C
AU         UA-      U      \ --        -         AUA
```

```
>sja-let-7   CCON0000102591.1 19497 19658 +
CAAUGCUGUGAUUCUGGGAGGUAGUUCGUUGUGUGGUUUGCUUAUUAAAAUGAUCUUAAGAAGACCAUACAACCGACUGGCUUUCCCACUGUG
CAGCAUUA

C        G  UC     --|G     C            G    UUAAA
 AAUGCUGU AU  UGGGA  G UAGUU GUUGUGUGGUUU CUUA     A
 UUACGACG UG  ACCCU  C GUCAG CAACAUACCAGA GAAU     U
A        -  UC     UU^G     C            A    UCUAG
```

```
>sja-miR-2b   CCON0000096838.1 242651 242727 -
UACCUUUUGCACCGCAGUCAAUAUUGGCUGAUGGCAUUGUUUUAUUGUCACAGCCAGUAUUGAUGAACGGGGUAAUAGGCG

UA   U    A   CA-|             A     UUGU
  CCU UUGC CCG   GUCAAUAUUGGCUG UGGCA    \
  GGA AAUG GGC   UAGUUAUGACCGAC ACUGU    U
GC   U    G   AAG^             -     UAUU
```

```
>sja-miR-124   CCON0000097036.1 113700 113785 +
UAUGCCAUUUUCCGCGAUUGCCUUGAUUUGUUAAAAGAAAAUGAUUCACAACAAAAUAUUAAGGCACGCGGUGAAUGUCAUC

U   C     U     AU         ---|    AAAA   AA
 AUG CAUUU CCGCG  UGCCUUGAU   UUGUU    GAA  \
 UAC GUAAG GGCGC  ACGGAAUUA   AACAA    CUU  U
C   U     U     --         UAA^    CA--   AG
```

```
>sja-miR-36a   CCON0000096812.1 593523 593727 -
CUUGCGAAGAAUGCCGAUCCGGUCAGCCAUUCAACAAUCUAUCAGUAAUAAACGGCCACCGGGUAGACAUUCAUUCGCAAG

        -|    CCG       CA   AUUCAACAAUCU
CUUGCGAA GAAUG   AUCCGGU  GCC            \
GAACGCUU CUUAC   UGGGCCA  CGG            A
        A^    AGA       C-   CAAAUAAUGACU
```

```
>sja-miR-10   CCON0000096836.1 310017 310097 +
AUUUUGAUUCCUCAGUAUGAACCCUGUAGACCCGAGUUUGGAUGCCGUUAGAUGCAAAUUCGAGUCUAUAAGGAAAGAUACUUUGGAAGUAGA
CG

AU    A    UC     GAA-| C       C        G  GCC
  UUUG UUCC  AGUAU    CC UGUAGAC CGAGUUUG AU   G
  AGAU AAGG  UCAUA    GG AUAUCUG GCUUAAAC UA   U
GC    G    UU     GAAA^ A       A        G  GAU
```

```
>sja-miR-219   CCON0000096831.1 351781 351921 -
ACAAUCGAUUCACUGAUUGUCCAUUCGCAUUUCUUGUUAGAAACAUUUUCAAUUAUUAUCAUUAUUCAAUGAUAAUGAUGAUAAUAUUUAAUU
CACAAGAGGUGUUAAUGGACAUCAUAUGAUCGAUUAUUAAUCAUCUGUUGAGU

AC        CAC   U        C           UAG--|  C   U   A           A
  AAUCGAUU   UGA UGUCCAUU GCAUUUCUUGU     AAA AUU UCA UUAUUAUCAUU U
  UUAGCUAG   ACU ACAGGUAA UGUGGAGAACA     UUU UAA AGU AGUAAUAGUAA U
--        UAU   -        U           CUUAA^  A   U   -           C
```

```
>sja-miR-8   CCON0000098567.1 88856 89020 +
UAAGAAUGAGUAUGGCAUCUUACUAAUAGUAUUUGAUAAAGAAUUUUCUAAUACUGUUAGGUAAAGAUGCCAGCUCACUUCUGAA

UA-    -     A         A--|          U  UAAA
   AGAA UGAGU UGGCAUCUU   CUAAUAGUAUU GA    G
   UCUU ACUCG ACCGUAGAA   GAUUGUCAUAA CU    A
AAG    C     -         AUG^          U  UUUA
```

```
>sja-miR-2a   CCON0000096838.1 242556 242635 -
CUGCAGGUGGUGCGCGUCUCAAAGGACUGUGAGCCAACGUAAUUACUGUAUCACAGCCCUGCUUGGGACACAGCCUACCUGCUU

CU        U  GC-       --|  A      GC  ACGU
  GCAGGUGG GC   GUCUCAA  AGG CUGUGA  CA    A
  CGUCCAUC CG   CAGGGUU  UCC GACACU  GU    A
UU        -  ACA       CG^  C      AU  CAUU
```

```
>sja-miR-36b   CCON0000096812.1 621764 621997 -
AGCAGCAUCUGAAGGGAAUGUCUGUUCGGUUUGUGUUAUCGUGGCUACUUAUUAAGUACUACUUAAUAGUGUAGUUAUGUUUCAGUGCACCAC
CGGGUAGACAUUCAUCCGCAGAAGUC

AGCA  A    AAGG-         UU    UU    UAU----         -|        UA
    GC UCUG     GAAUGUCUG  CGGU  GUGU       CGUGGCUAC UUAUUAAG  \
    UG AGAC     CUUACAGAU  GCCA  CACG       GUAUUGAUG GAUAAUUC  C
C---  A    GCCUA         GG    C-    UGACUUU         U^       AU
```

```
>sja-miR-1810   CCON0000102009.1 933 1126 +
GAUUGUUCACCCACUAAUAGGGAACGUGAGCUGGGUUUAGACCGUCGUGAGACAGGUUAGUUUUACCCUACUAAUGAGUACGUCAUUUUAUUC
AGUGAAGUACAAUCU

-      UCACC    -       A-     ------  CU    UUA---    -|  G
 GAUUGU     CACU AAUAGGG  ACGUG      AG  GGGU      GACC GUC U
 CUAACA     GUGA UUAUUUU  UGCAU      UC  CCCA      UUGG CAG G
```

```
U      UGAA-    C       AC     GAGUAA  AU    UUUUGA    A^  A

>sja-miR-281   CCON0000101257.1 19664 19883 +
UUCUGAUAGAGAGAGCACUUUUAUGACGGAGACGAAUAUUAUCUUCGAAUUUUAUUUUGAAGUUCCAUAAUGUCAUGGAGUUGCUCUCUAUAU
CAGGU
U|      G        C          GAGAC   UAUUAU       UU
 UCUGAUA AGAGAGCA UUUUAUGACG     GAA      CUUCGAA  U
 GGACUAU UCUCUCGU GAGGUACUGU     CUU      GAAGUUU  U
U^      A        U          AAUAC   ------       UA

>sja-miR-76   CCON0000097263.1 45083 45251 +
UAUACUACACAGUUUCUUUGACAAUGAGCUUAACUGACAUGCAGCAUAUAUAUAUUUAUAUAUGUCUGUACAAUAUGUUCGUUGUUGAUGAAA
CUGGACAGUUACAC

UAU--|  ACA       U UG         UUAAC  ACA     -        U
     ACU   CAGUUUC U  ACAAUGAGC     UG   UGCAG CAUAUAUA A
     UGA   GUCAAAG A  UGUUGCUUG     AC   AUGUC GUAUAUAU U
CACAU^  CAG       U GU         UAUA-  ---     U        U

>sja-miR-307   CCON0000100804.1 7236 7453 +
AAGAAACUCCUCAUAAAGAAGGUUGUUUGAUGUAAUAUUCAAUAAUUAAUAAUAAUUAUUAUUAUUAUUAUUAUCAUUUCGAUGAAAUAAUAA
UAUAAAGAAGAUGAAUAUAAAAUAACUUACAUCACAACCUACUUGAUUGAGGGGCACUU

AAGAAA       UA-   A       UU      ---------       .-A|     A
      CUCCUCA   AAG AGGUUGU  GAUGUA         AUAUUCA   UAAUUA U
      GGGGAGU   UUC UCCAACA  CUACAU         UAUAAGU   AUUAAU A
UUCAC-       UAG   A       --      UCAAUAAAA       \ -^     A

>sja-miR-923   CCON0000096984.1 233125 233191 -
AAGAAGCGGAGGAAAAGAAAUUGCAAAUUACAAUGAUGUUUGCUUUUCUAGAGCUUCAC

AA     GGA       -|   UG  AAU
  GAAGC   GGAAAAG AAAU  CA   U
  CUUCG   UCUUUUC UUUG  GU   A
CA     AGA       G^   UA  AAC
```
