# Supplementary material for: Identification and characterization of microRNAs and endogenous siRNAs in Schistosoma japonicum
Source: BMC Genomics. 2010 Jan 21;11:55. doi: 10.1186/1471-2164-11-55 (PMC2820009; doi:10.1186/1471-2164-11-55)
Supplement: Additional file 9 — Hairpin prediction of novel miRNAs in S. japonicum. This file contains the predicted hairpin structures of novel miRNAs. [file 1471-2164-11-55-S9.PDF]

Hairpin prediction of novel miRNAs in *S. japonicum*

```
>Sja-Novel-16   CCON0000096806.1 227364 227473 +
CGAUGUGAUAUGUAUGGGUUACUUGGUGUCAGCGGAUAACAUGUUUUAUUCGCCAUUAAGCAAUACACCAGUGACCAGACAUAUCCCUAUCG

     U-       AUG      U      CA---------|       CA
CGAUG  GAUAUGU   GGUUAC UGGUGU           GCGGAUAA  U
GCUAU  CUAUACA   CCAGUG ACCACA           CGCUUAUU  G
     CC       GA-      -      UAACGAAUUAC^       UU
```

```
>Sja-Novel-137   CCON0000097135.1 79109 79336 +
UGGAAAGAGGUAGUGAUUCAUAUGACUACUGUGUUACUCCUUGUGUGUGUGUAUGUAUAGAAAAUGUAAAAAAUAUGUUGUAACAACAUACUA
AACAUUGAAAAAAUUAAUCUUUUUCAAUGAAAUUUUCUUUUGUAUACACACAUAUAAGGUGGUUACAUUUUAAUGGAAGUCAUAUGUCUCAC
UAGCUUCAUAC

UGGAAA    -      UU         A  ------    U   -                   U-       .-GUAAAAAA|      U
      GAGG UAGUGA  CAUAUGACU CU      GUGU ACU CCUUGUGUGUGUGUAUGUA  AGAAAAU          UAUGUUG \
      CUUC AUCACU  GUAUACUGA GG      UACA UGG GGAAUAUACACACAUAUGU  UCUUUUA          AUACAAC A
CAUA--    G      CU         A  UAAUUU    U   U                   UU       \ --------^       A
```

```
>Sja-Novel-110   CCON0000097036.1 283486 283698 +
GGCAAGUCGGCUUUUAUUGCGCUCUGAGAAAAACCAAUUCAUUGCUUUUUUUAUUUAUCUGAGAUCGCGAUUAAAGCUGGUUUUCC

  C  GU       U      C   G   ---------    C   U
GG AA  CGGCUUU AUUGCG UCU AGA         AAAA CAA U
CC UU  GUCGAAA UAGCGC AGA UCU         UUUU GUU C
  U  UG       U      U   G   AUUUAUUUU    C   A
```

```
>Sja-Novel-148   CCON0000097165.1 353262 353355  -
GAAUAAAAUCCCUGAGACUGAUAAUUGCUCUAGUUAUUAUAUCAUUAAUGAGUUUACAAUAAGGGCAAUUAUUAUUCUCAGGUGUAUCAUUC

    AAA UC       C             A   AUUAUA   U
GAAU   A  CCUGAGA UGAUAAUUGCUCU GUU      UCA U
CUUA   U  GGACUCU AUUAUUAACGGGA UAA      AGU A
    CUA GU       U             A   CAUUUG   A
```

```
>Sja-Novel-70   CCON0000096911.1 157412 157649  -
AAACGUAACUUCAGCUGUGUUCAUGUCUUCGACAAAAAAUAAACCAACAAGUGAAUACGUAGGUAUUCAUGUUGAUUAAUAUCGAAGAAAUGA
GCACAACUUAGAUUGUUU

     UAA  UC  C         G       CAAAA   AAAC     AG       G
AAACG   CU  AG UGUGUUCAU UCUUCGA     AAU    CAACA  UGAAUAC U
UUUGU   GA  UC ACACGAGUA AGAAGCU     UUA    GUUGU  ACUUAUG A
     UA-  U-  A         A       AUAA-   ----     --       G
```

```
>Sja-Novel-168   CCON0000097253.1 21744 21821  -
GGGGAUGAGUGAGAUUGUUGCAUAUUUACAUUGCUGGUAUGUAUUAUGCAACGUUUCACUCUACUCC

    AU         U        UU     UGC
GGGG  GAGUGAGAU GUUGCAUA  UACAU   \
CCUC  CUCACUUUG CAACGUAU  AUGUA   U
    AU         -        U-     UGG
```

>Sja-Novel-166   CCON0000097242.1 178483 178721 +

ACGGUUGGAGUUUUUGUUUCUGCACCAGCCACAUAUCAGUCUGAUUGAUACUCCUCACUGUAGGAGAUUUUGUCAAAUGAACUCUGGUGAGAUUCAAUUACUUCAACUGU

```
|          UU   UU   G      CCACAUA   GUC    U-   A     CA
ACGGUUGGAGU  UUG  UCU CACCAG       UCA   UGAU  GAU CUCCU  C
UGUCAACUUCA  AAC  AGA GUGGUC       AGU   ACUG  UUA GAGGA  U
           UU   UU   -      UCA----   AA-    UU   -     UG
```

>Sja-Novel-37   CCON0000096839.1 380037 380168 -

UUGUGAGGUGGAUGUUAGUGCAAUUUAGUGACGUAAAUAUAUUGCACUUACCUUCGCCUUGCAAAUAU

```
----|  UG       U  U        U  GUGA
    UUG  AGGUGGA GU AGUGCAAU UA    C
    AAC  UCCGCUU CA UCACGUUA AU    G
UAUA^  GU       C  U        U  AAAU
```

>Sja-Novel-173   CCON0000097277.1 121319 121551 +

CUGUAAGCCAGACUCACAGAAAUGCUAACAUACAACGAAAAGGAUUCUUAGAAGAUAAGUAAAAAAUAAGUUUCAUCAUCUCUAGCAUUUUUUUGUGUUCUACAG

```
     A CCA   U  C          ACAUACAAC    AGG   -----    GA
CUGUA G   GAC CA AGAAAUGCUA         GAAA   AUU     CUUA   \
GACAU C   UUG GU UUUUUACGAU         CUUU   UAA     GAAU   A
     - ---   U  U          CUCUACUA-    GAA   AAAAU    AG
```

>Sja-Novel-245   CCON0000098901.1 7322 7458 +

CGUUUUCUUUGGUUAUCAAGCAAUAUGAUCGCAUGUCUUCAUAUAUGGAUUCAUUUGUUCCAAAUGUUAUCCUACUUAUCAUAAAGCUCGAUAAUCAAAGUUAAACG

```
     U-           A   AA      .-CGC|  U
CGUUU  CUUUGGUUAUC AGC  UAUGAU     AUG C
GCAAA  GAAACUAAUAG UCG  AUACUA     UAC U
     UU           C   AA      \ ---^  U
```

>Sja-Novel-239   CCON0000098567.1 54884 54965 -

CGACCGCAAGACAUCAAAUAUUUUGUCUGCUAGCUCUGCUAACUCUGAUAAGGGAGUAGAGUUUACGGAAAUGCUGAGAAUCUGUUGGAUGUUGUAUGAAAG

```
CGACCGCAA      A    UU  G  U  U--------|        AA    G
         GACAUC AAUA  UU UC GC         AGCUCUGCU  CUCU A
         UUGUAG UUGU  AA AG CG         UUGAGAUGA  GGGA U
GAAAGUAUG      G    CU  G  U  UAAAGGCAU^        --    A
```

>Sja-Novel-255   CCON0000099623.1 9776 9958 +

CAUGGGAGGAUGUGGUGACCAGGUCGGUGUCCGAGUUCGACAGUCCGGCGGAUAGGGAGUUGGCGUUGCAGCAGUUCAGGGCCGUGCUU

```
---     ----   GA--|  GG  A    G  GG      AGU   AC
   CAUGG    GAG    UGU  UG CCAG UC  UGUCCG   UCG  A
   GUGCC    CUU    ACG  GC GGUU AG  AUAGGC   GGC  G
```

```
UUC     GGGA   GACG^  UU  -    G  GG      ---   CU

>Sja-Novel-120   CCON0000097060.1 25147 25310 +
CACUUCUGACAAACAACUGGAGGCUUUUCGUCUCUGGCACUACGAUUCCAGGAUGUAACAGAGACGAGGGCGCUGCAGGGGUUUAAAUG

CACUUCUGAC    AA   G  -|    U        GC  UACGAU
          AAAC  CUG AG GCUUU CGUCUCUG  AC       U
          UUUG  GAC UC CGGGA GCAGAGAC  UG       C
GUAAAU----    GG   G  G^    -        AA  UAGGAC

>Sja-Novel-121   CCON0000097060.1 25805 25945 +
GACUUCGGAUAACUGGAGUAUCCUGGACGAAUGUUUUGGAGGUUGCCAAGACGCGUCACGACAUUUCAGGCAGUUCAUGGAUA

GA-  UC    AA-|      A  CUG    AA         AG
   CU  GGAU   CUGGAGU UC   GACG  UGUUUUGG  G
   GG  CUUG   GACUUUA AG   CUGC  GCAGAACC  U
AUA  UA    ACG^      C  CA-    --         GU

>Sja-Novel-35   CCON0000096922.1 152466 152527  -
AGUGUGACUGUCUGGACACAGUAGCCUAGUGGUUAACGCGGUGGCGUUUGACACGAAGCUGACUGAGUUCGAAUCCCAGUGU

AGUGUG    UC---     A   -|   CUA   GUU     G
      ACUG     UGGAC CAG UAGC   GUG   AACGC G
      UGAC     GCUUG GUC GUCG   CAC   UUGCG U
UG----    CCUAA     A   A^   AAG   AGU     G

>Sja-Novel-36   CCON0000096838.1 242463 242530  -
UCCCACCGCUCUUACCAACUUAGACUGAGUUAUACUGCUACUGUAAGCUUUGUAUAUCACAGUCCAAGCUUUGGUAAGUUUUGAUGGG

U    C  CU-        --   A     AGU     U--|  AC
 CCCA CG   CUUACCAA  CUU GACUG   UAUAC   GCU  U
 GGGU GU   GAAUGGUU  GAA CUGAC   AUAUG   CGA  G
-    A  UUU        UC   C     ACU     UUU^  AU

>Sja-Novel-147   CCON0000097154.1 70837 70934  -
AUGCUAUUUGUUGUUGGCAAGAUUACGGCGAAGCUGAAUCAAAUUUAAAAUGUUUCAGCUUCACUUUAUUUUGCCAAUAAAGGUAGCAU

         G             UAC  C         U  AAUU
AUGCUAUUU UUGUUGGCAAGAU   GG GAAGCUGAA CA    \
UACGAUGGA AAUAACCGUUUUA   UC CUUCGACUU GU    U
         -             UU-  A         U  AAAA

>Sja-Novel-128   CCON0000097086.1 425137 425311  -
UCGAAGCGUUCGGACGUUGGCACUUUACGCAGAUAUGCUGUAGUUCGAAGCUUC

UC|    G       GUU    CU  AC
  GAAGC UUCGGAC   GGCA  UU  \
  CUUCG AAGCUUG   UCGU  AG  G
--^    -       AUG    AU  AC
```

>Sja-Novel-203 CCON0000097678.1 158866 159035 -

ACACACAAGUUAUAUUUAAGUUGGAUUUUGACUAACAAGUGGAAUACAGGACGAGCGUUUCGUUCUAUUUGGGAUGCAGGUACAUCCAGCCAAUCAGUCCCAAAUAAGACGAAACGCGUAUUGUAGAUUCUACCUGCUGGUCACAAUCCAACUUAAACAUACUUUUCUGU

```
   CAC   U   A             U      A  A      --|    GACGA         UC          GCA--   ACA
ACA   AAG UAU UUUAAGUUGGAUU UGACUA CA GUGGAA  UACAG     GCGUUUCGU  UAUUUGGGAU     GGU   \
UGU   UUC AUA AAAUUCAACCUAA ACUGGU GU CAUCUU  AUGUU     CGCAAAGCA  AUAAACCCUG     CCG   U
   CUU   -   C             C      C  C      AG^    AUG--         GA          ACUAA   ACC
```

>Sja-Novel-21 CCON0000096809.1 725995 726085 +

CCAAGUAAACUAAGUUCUGAUAGAUGUUGCACUUAUUAUACGCACUUAUAUUGACUGUCAAUAUAGUAUUUAUUCUAUUUGUGACUGUUUAUAAGACAUUACAAACUCGAU

```
CCA-|  AAAC   GU   G      -  UG  CU  UU   CGC   U      AC
    AGU    UAA  UCU AUAGAU GU  CA  UA  AUA   ACU AUAUUG  \
    UCA    AUU  AGA UAUUUG CA  GU  AU  UAU   UGA UAUAAC  U
UAGC^  AAC-   AC   A      U  GU  UU  CU   UUA   -      UG
```
