# Supplementary material for: Identification and characterization of microRNAs and endogenous siRNAs in Schistosoma japonicum
Source: BMC Genomics. 2010 Jan 21;11:55. doi: 10.1186/1471-2164-11-55 (PMC2820009; doi:10.1186/1471-2164-11-55)
Supplement: Additional file 10 — Common miRNAs identified in S. japonicum. This file contains the information of common miRNAs, from contig identity to stage-associated variation in transcription. [file 1471-2164-11-55-S10.PDF]

Common miRNA identified in *S. japonicum*

MicroRNA Name	Hairpin Name	Genome Loci	Mature Arm	miR*[a]	Expression[c]				Most abundant sequence	Len
					Total	Adu	schistosomula	P-value		
sja-let-7	CCON0000102591.1_19497_19658_+	InterGenic	5'	Y	30328	15907	14421	0.0000	GGAGGUAGUUCGUUGUGUGGU	21
sja-MIR-10	CCON0000096836.1_310017_310097_+	InterGenic	5'	Y	3663	1431	2232	0.0000	AACCCUGUAGACCCGAGUUUGG	22
sja-MIR-124	CCON0000097036.1_113700_113785_+	InterGenic	3'	Y	11221	9259	1962	0.0000	UAAGGCACGCGGUGAAUGUCA	21
sja-MIR-1810	CCON0000102009.1_933_1126_+	InterGenic	5'	N	80	49	31	0.3083	CUAAUAGGGAACGUGAGCU	19
sja-MIR-219	CCON0000096831.1_351781_351921_-	InterGenic	5'	Y	3487	2965	522	0.0000	UGAUUGUCCAUUCGCAUUUCUUG	23
sja-MIR-281	CCON0000101257.1_19664_19883_+	InterGenic	3'	Y	64	60	4	0.0000	UGUCAUGGAGUUGCUCUCUAU	21
sja-MIR-2a	CCON0000096838.1_242556_242635_-	InterGenic	3'	Y	1029	978	51	0.0000	UAUCACAGCCCUGCUUGGGACACA	24
sja-MIR-2b	CCON0000096838.1_242651_242727_-	InterGenic	3'	Y	29644	24040	5604	0.0000	UCACAGCCAGUAUUGAUGAACG	22
sja-MIR-307	CCON0000100804.1_7236_7453_+	InterGenic	3'	Y	23	22	1	0.0001	UCACAACCUACUUGAUUGAGG	21
sja-MIR-36a	CCON0000096812.1_593523_593727_-	InterGenic	3'	Y	7550	4293	3257	0.0263	CCACCGGGUAGACAUUCAUUCGC	23
sja-MIR-36b	CCON0000096812.1_621764_621997_-	InterGenic	3'	Y	111	55	56	0.2002	CCACCGGGUAGACAUUCAU	19
sja-MIR-7	CCON0000097602.1_12747_12871_+	InterGenic	5'	Y	76171	25549	50622	0.0000	UGGAAGACUGGUGAUAUGUUGUU	23
sja-MIR-71	CCON0000096885.1_360273_360432_+	InterGenic	5'	Y	266624	100935	165689	0.0000	UGAAAGACUUGAGUAGUGAGACG	23
sja-MIR-76	CCON0000097263.1_45083_45251_+	InterGenic	3'	Y	24	24	0	0.0000	UUCGUUGUUGAUGAAACUGG	20
sja-MIR-8	CCON0000098567.1_88856_89020_+	InterGenic	3'	N	1351	953	398	0.0000	UAAUACUGUUAGGUAAAGAUGCC	23
sja-MIR-923	CCON0000096984.1_233125_233191_-	Intron	5'	N	3	3	0	0.1216	AAGCGGAGGAAAAGAAAU	18
