# Supplementary material for: Identification and characterization of microRNAs and endogenous siRNAs in Schistosoma japonicum
Source: BMC Genomics. 2010 Jan 21;11:55. doi: 10.1186/1471-2164-11-55 (PMC2820009; doi:10.1186/1471-2164-11-55)
Supplement: Additional file 11 — Hairpin prediction of novel miRNAs in S. japonicum. This file contains the predicted hairpin structures of the novel miRNAs, from contig identity to stage-associated variation in transcription. [file 1471-2164-11-55-S11.PDF]

Novel miRNAs identified in *S. japonicum*

Family	miRNA Name	Hairpin Name	Genome Loci	Mature Arm	miR*[a]	Expression[c]				Most abundant sequence	Len
						Total	Adu	Schistosomula	P-value		
16	Sja_Novel16	CCON0000096806.1_227364_227473_	Intron	5'	*	109301	100868	8433	0	UGAUAUGUAUGGGUUACUUGGU	22
20	Sja_Novel20	CCON0000096808.1_661078_661297_	InterGenic	5'	*	3	2	1	0.699421	UGUCUUUGUCAAUGUUGGGC	20
21	Sja_Novel21	CCON0000096809.1_725995_726085_	InterGenic	5'	*	121	95	26	0	AAGUUCUGAUAGAUGUUGCA	20
36	Sja_Novel36	CCON0000096838.1_242463_242530_	InterGenic	3'	*	135	59	76	0.00544	UAUCACAGUCCAAGCUUUGGUAA	23
37	Sja_Novel37	CCON0000096839.1_380037_380168_	InterGenic	3'	*	1356	1012	344	0	UAUUGCACUUACCUUCGCCUUG	22
48	Sja_Novel48	CCON0000096854.1_157002_157111_	InterGenic	5'	*	4	4	0	0.073844	UUAACGAUAACGGAAUCUC	19
53	Sja_Novel53	CCON0000096856.1_83722_83915_-	Intron	5'	*	18	13	5	0.155569	UCCCUAACGUUUUCUGUCAA	20
57	Sja_Novel57	CCON0000096873.1_480302_480532_	InterGenic	5'	*	16	15	1	0.002126	GUUAUGUAUACUGUAUGGCG	20
59	Sja_Novel59	CCON0000096874.1_238177_238320_	InterGenic	5'	*	32	18	14	0.940171	AGGUAUCAGUCGGUUCGUCA	20
62	Sja_Novel62	CCON0000096879.1_142615_142769_	InterGenic	5'	*	40	34	6	0.000181	UAUAACAGACGGUCGACCAA	20
70	Sja_Novel70	CCON0000096911.1_157412_157649_	InterGenic	5'	*	5060	5060	0	0	UCAGCUGUGUUCAUGUCUUCGA	22
82	Sja_Novel82	CCON0000096945.1_53141_53295_+	InterGenic	5'	*	100	99	1	0	GUCCCGCGUGGUCUAGCGGUUAG	23
95	Sja_Novel95	CCON0000096988.1_59152_59271_-	InterGenic	3'	*	12	4	8	0.120717	GGUGAUCUUUGUAUGGACAA	20
110	Sja_Novel110	CCON0000097036.1_283486_283698_	InterGenic	3'	*	38923	38885	38	0	UGAGAUCGCGAUUAAAGCU	19
112	Sja_Novel112	CCON0000097049.1_44816_45004_-	InterGenic	3'	*	43	23	20	0.781422	AAGACUUCGAUCUUGUGAUU	20
137	Sja_Novel137	CCON0000097135.1_79109_79336_+	InterGenic	5'	*	73746	52828	20918	0	AGAGGUAGUGAUUCAUAUGACU	22
138	Sja_Novel138	CCON0000097136.1_266862_267081_	InterGenic	5'	*	30	21	9	0.112192	GCUUGCGGUCGUUGAGGG	18
148	Sja_Novel148	CCON0000097165.1_353262_353355_	InterGenic	5'	*	34802	29343	5459	0	UCCCUGAGACUGAUAAUUGCU	21
152	Sja_Novel152	CCON0000097177.1_193443_193573_	InterGenic	5'	*	24	20	4	0.006231	CAAGUCAUCUAUCAGAGCAC	20
156	Sja_Novel156	CCON0000097189.1_208458_208637_	InterGenic	3'	*	34	30	4	0.000128	UGUGCGUAGUUUCAUUGACUAGU	23
166	Sja_Novel166	CCON0000097242.1_178483_178721_	InterGenic	3'	*	2117	2071	46	0	UGAGAUUCAAUUACUUCAACU	21
168	Sja_Novel168	CCON0000097253.1_21744_21821_-	InterGenic	3'	*	3620	983	2637	0	UAUUAUGCAACGUUUCACUCU	21
190	Sja_Novel190	CCON0000097535.1_188799_188875_	InterGenic	5'	*	44	33	11	0.009565	UAGUGAGGUGGAGGUAGGUA	20
221	Sja_Novel221	CCON0000097957.1_22468_22585_-	InterGenic	5'	*	49	10	39	0.000001	UGUAGAAUUUAGAUGCAUAU	20
245	Sja_Novel245	CCON0000098901.1_7322_7458_+	InterGenic	5'	*	333	274	59	0	UCUUUGGUUAUCAAGCAAUAUGA	23
250	Sja_Novel250	CCON0000099059.1_7170_7299_+	InterGenic	3'	*	101	97	4	0	UGACGUCGUAAGCCAGAGUA	20
259	Sja_Novel259	CCON0000100429.1_4201_4362_+	InterGenic	3'	*	39	23	16	0.670633	UGGAUUGAAUAGCAUUUGUAU	21
277	Sja_Novel277	CCON0000119810.1_241_336_-	InterGenic	5'	*	6	6	0	0.028573	CAUAAUAUCGAUGCCAACU	19
281	Sja_Novel281	CCON0000104611.1_6507_6639_+	InterGenic	3'	*	34	24	10	0.078403	GAUUCCUAUCGAUAGUCGCC	20
288	Sja_Novel288	CCON0000105615.1_2471_2676_+	InterGenic	5'	*	39	7	32	0.000002	AAACCGACUGUUGGCUAUUC	20
312	Sja_Novel312	CCON0000112973.1_947_1156_+	InterGenic	5'	*	13	12	1	0.007712	AUUGACAAACCUUUGAGGCA	20
318	Sja_Novel318	CCON0000114308.1_1039_1134_+	InterGenic	3'	*	90	78	12	0	AGUUAUAAACCUAAUCGCCG	20
323	Sja_Novel323	CCON0000116400.1_2439_2509_+	Intron	3'	*	6	4	2	0.585045	UGAAUAGCCCGUAGAUCUAUGC	22
327	Sja_Novel327	CCON0000119484.1_1335_1573_-	InterGenic	5'	*	105	39	66	0.000142	GCUGGGAUGGCCGAGUGGUUA	21
328	Sja_Novel328	CCON0000120016.1_2575_2732_-	InterGenic	5'	*	12	5	7	0.331659	AAGCCGAAGGCUAGUUGAACU	21
331	Sja_Novel331	CCON0000120343.1_1148_1271_+	InterGenic	3'	*	29	23	6	0.010146	UCGGGUUGUGGGGUUGGUUA	20
333	Sja_Novel333	CCON0000121113.1_1034_1114_-	InterGenic	5'		6	5	1	0.171412	AGUAAACUAAAUUCUGAUAG	20
334	Sja_Novel334	CCON0000121249.1_2279_2360_-	InterGenic	5'	*	8	8	0	0.011471	UCAUGCUGGCAUUGUGGCGC	20
